# Characterization of a library of 20 HBV-specific MHC class II-restricted T cell receptors

**DOI:** 10.1016/j.omtm.2021.10.012

**Published:** 2021-10-29

**Authors:** Sophia Schreiber, Melanie Honz, Weeda Mamozai, Peter Kurktschiev, Matthias Schiemann, Klaus Witter, Eugene Moore, Christina Zielinski, Alessandro Sette, Ulrike Protzer, Karin Wisskirchen

**Affiliations:** 1 Institute of Virology, Helmholtz Zentrum München, 81675 Munich, Germany; 2 Institute of Virology, School of Medicine, Technical University of Munich, 81675 Munich, Germany; 3 Medical Department II and Institute for Immunology, Hospital of the Ludwig-Maximilians-University (LMU) Munich, 81377 Munich, Germany; 4 Institute for Medical Microbiology, Immunology and Hygiene, School of Medicine, Technical University of Munich, 81675 Munich, Germany; 5 German Center for Infection Research (DZIF), Munich Partner Site, Munich, Germany; 6 Laboratory for Immunogenetics and Molecular Diagnostics, Hospital of the Ludwig-Maximilians-University (LMU) Munich, 81377 Munich, Germany; 7 Center for Infectious Disease and Vaccine Research, La Jolla Institute for Immunology (LJI), La Jolla, CA 92037, USA; 8 Department of Medicine, Division of Infectious Diseases and Global Public Health, University of California, San Diego (UCSD), La Jolla, CA 92037, USA

**Keywords:** MHC class II-restricted T cell receptors, CD4^+^ T cells, chronic hepatitis B, adoptive T cell therapy, hepatocellular carcinoma, T cell help, retroviral transduction, hepatitis B virus infection, HBV clearance, TCR expression

## Abstract

CD4^+^ T cells play an important role in the immune response against cancer and infectious diseases. However, mechanistic details of their helper function in hepatitis B virus (HBV) infection in particular, or their advantage for adoptive T cell therapy remain poorly understood as experimental and therapeutic tools are missing. Therefore, we identified, cloned, and characterized a comprehensive library of 20 MHC class II-restricted HBV-specific T cell receptors (TCRs) from donors with acute or resolved HBV infection. The TCRs were restricted by nine different MHC II molecules and specific for eight different epitopes derived from intracellularly processed HBV envelope, core, and polymerase proteins. Retroviral transduction resulted in a robust expression of all TCRs on primary T cells. A high functional avidity was measured for all TCRs specific for epitopes S17, S21, S36, and P774 (half-maximal effective concentration [EC_50_] <10 nM), or C61 and preS9 (EC_50_ <100 nM). Eight TCRs recognized peptide variants of HBV genotypes A to D. Both CD4^+^ and CD8^+^ T cells transduced with the MHC II-restricted TCRs were polyfunctional, producing interferon (IFN)-γ, tumor necrosis factor (TNF)-α, interleukin (IL)-2, and granzyme B (GrzB), and killed peptide-loaded target cells. Our set of MHC class II-restricted TCRs represents an important tool for elucidating CD4^+^ T cell help in viral infection with potential benefit for T cell therapy.

## Graphical abstract

**Figure:**
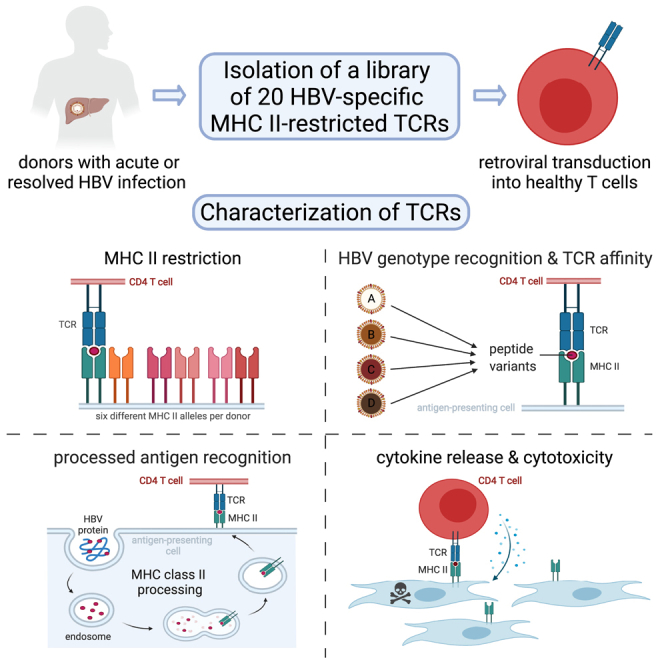

## Teaser

CD4^+^ T cells are important in clearance of hepatitis B virus (HBV) infection. We characterized the specificity and functional profile of 20 HBV-specific MHC II-restricted T cell receptors. They are a useful tool to elucidate HBV-specific T cell function and their role in T cell therapy of HBV-associated diseases.

## Introduction

Chronic hepatitis B (CHB) continuous to be a global health problem, with 296 million people affected worldwide.^1^ Current treatment options, such as nucleos(t)ide analogues and pegylated interferon (IFN)-α, are rarely able to cure the infection, and chronically infected patients remain at an elevated risk of developing liver cirrhosis and eventually hepatocellular carcinoma (HCC) during their lifetimes. Chronic hepatitis B virus (HBV) infection is marked by a progressive functional exhaustion and ultimately depletion of virus-specific CD4^+^ and CD8^+^ T cells.^2^ Naturally resolving, acute hepatitis B, on the other hand, is characterized by a strong and polyfunctional T cell response, which can be restored in CHB patients by transfer of HBV-specific T cells.^3^ The therapeutic potential of transferring HBV immunity initially emerged through clinical reports of CHB patients who cleared the infection after receiving a bone marrow transplant from HBV-immune donors, giving rise to HBV-specific CD4^+^ and CD8^+^ T cells as well as B cells.^4^^,^^5^ Hence, adoptive T cell therapy represents a promising therapeutic approach to treat CHB- and HBV-induced HCC.^6^ In order to imitate such an effective immune response needed for clearing the infection, we have previously generated both HBV-specific chimeric antigen receptors (CARs)^7^ and MHC class I (MHC I)-restricted T cell receptors (TCRs)^8^ that showed promising antiviral activity in models of CHB.^9^^,^^10^ In a humanized mouse model, in which transplanted human hepatocytes were infected with HBV, MHC I-restricted HBV-specific T cells even led to undetectable serum levels of HBV surface antigen and HBV DNA,^10^ a status that is described as functional cure.^11^ However, when T cells vanished, a viral rebound could only be contained with an HBV entry inhibitor,^10^ underlining the necessity for long-term persistence of anti-HBV immunity.

Although cytotoxic CD8^+^ T cells are key to clearing HBV infection by destroying infected cells, CD4^+^ T cells are known to play an important role.^12^^,^^13^ They are indispensable for viral clearance as, for example, chimpanzees are unable to clear HBV infection when CD4^+^ T cells are depleted early on during the course of infection.^14^ Besides their direct role in targeting infected or malignant cells, CD4^+^ T cells provide help to other immune cells. For instance, they license dendritic cells to cross-present viral antigen to CD8^+^ T cells,^15^ and CD8^+^ T cells that have received CD4^+^ T cell help during priming and second antigen encounter differentiate to memory cells more effectively, proliferate better, and increase their migratory and invasive potential.^16^ Moreover, CD4^+^ T cells can induce maturation and antibody production in B cells of the same antigen specificity. This interaction requires the engagement of an MHC class II (MHC II)-restricted TCR specific for an antigen-derived peptide with the peptide:MHC complex (pMHC) on a B cell that has taken up the same antigen via its B cell receptor.^17^ Little is known about the detailed functionality of HBV-specific CD4^+^ T cells as the few available studies have focused on describing their *ex vivo* immunophenotypes. Presumably they exert an indirect role in viral clearance by helping CD8^+^ T cells and B cells, which is especially important in fighting a poorly immunogenic virus like HBV.^18^ Recently, a higher frequency of HBV-specific CD4^+^ T cells was positively correlated with hepatitis B e antigen (HBeAg) or hepatitis B surface antigen (HBsAg) loss after flares in CHB patients.^19^ Hence, overcoming the low numbers and dysfunctional phenotype of HBV-specific CD4^+^ T cells in CHB^20^ might also promote viral clearance.

Along that line, the importance of CD4^+^ T cells in fighting viral infections and tumor diseases also implies their benefit for immunotherapy and potentially for adoptive T cell therapy,^21^^,^^22^ including treatment of CHB- and HBV-induced HCC. To date, most T cell therapeutic approaches have focused on tumor diseases using genetically engineered T cells expressing an MHC I-restricted TCR or a CAR, and those T cell products typically also include redirected CD4^+^ T cells. Co-transfer of these CD4^+^ T cells can confer superior therapeutic efficacy to some extent^23^^,^^24^ but does not provide the potential benefits of CD4^+^ T cells naturally engaging with peptides presented on MHC II.^21^ In mice, they were shown to alter the tumor microenvironment through interaction with antigen-presenting cells (APCs)^25^ and were required for the recruitment and cytolytic function of CD8^+^ T cells.^26^ In addition, IFN-γ-dependent interaction of CD4^+^ T cells with non-hematopoietic cells was shown to interfere with tumor angiogenesis.^27^ Moreover, clinical evidence is encouraging as a metastatic patient who received autologous NY-ESO-1-specific CD4^+^ T cell clones reportedly went into complete remission after developing an endogenous multi-specific T cell response.^28^ Although the application of MHC II-restricted T cells in T cell therapy has recently gained more attention,^21^^,^^22^ only a few TCRs have been isolated to date, including one TCR against human papilloma virus (HPV) E7 as a viral target.^29^ Indeed, the addition of redirected MHC II-restricted CD4^+^ to MHC I-restricted CD8^+^ T cells was shown to strongly increase tumor regression in a xenograft mouse model.^30^ These results emphasize the potential that can be attributed to the use of CD4^+^ T cells in adoptive T cell therapy, also in the context of viral infection.

In order to address the lack of knowledge on HBV-specific CD4^+^ T cells and to improve the success rate of adoptive T cell transfer, experimental and therapeutic tools are needed. In the present study, we isolated MHC II-restricted TCRs from HBV-specific CD4^+^ T cells. These TCRs target different epitopes of the HBV core, envelope, and polymerase proteins and were extensively characterized regarding MHC restriction, binding affinity, and recognition of antigen, so as to evaluate them and compare their overall applicability in adoptive T cell therapy of HBV infection.

## Results

### TCRs isolated from HBV-specific CD4^+^ T cells are expressed at high levels after retroviral transduction

**Figure 1:**
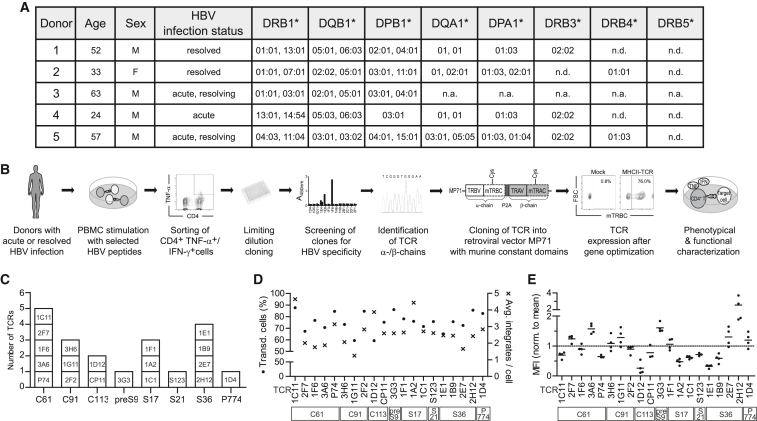
Identification and cloning of TCRs from HBV-specific CD4^+^ T cell clones (A) MHC II alleles of donors 1–5 with acute or resolved HBV infection. For donor 3, high-resolution MHC II typing was not available (n.a.) due to limited material resources. n.d., none detected. (B) Overview of the procedure: PBMCs from donors with acute or resolved HBV infection were stimulated with 1 μM of selected HBV peptides derived from core, envelope, or polymerase proteins. After two weeks, HBV-specific TNF-α or TNF-α/IFN-γ-secreting CD4^+^ T cells were sorted by fluorescence-activated cell sorting and expanded from a single-cell level by limiting dilution cloning. After two further weeks of expansion with the addition of feeder cells and IL-2, clones were screened for HBV specificity and TCR α and β chains were identified by Sanger sequencing. Codon-optimized variable α and β domains (TRAV and TRBV) were cloned into the retroviral vector MP71 in combination with murine constant domains (mTRAC and mTRBC), including an additional cysteine (Cys) residue to increase pairing. TCR transduction rates were determined via flow cytometry by staining the mTRBC, here plotted against the cell volume (forward scatter, FSC). TCRs were phenotypically and functionally characterized through co-cultures of TCR-transduced T cells with peptide-pulsed target cells; i.e. HLA-matched B-LCLs or fibroblasts. (C) Final panel of identified TCRs. Clone and TCR names are indicated in square boxes. Peptide specificities from HBV core (C), envelope (preS/S), or polymerase (P) proteins are written below with the number indicating the peptide starting residue within the respective antigen. (D) Transduction rates (•, left y-axis) and average number of integrates per cell (x, right y-axis) of a representative cell batch. The average vector copy number per bulk cell population, i.e., including both transduced and non-transduced cells, was measured in a multiplex qPCR of the viral woodchuck hepatitis virus postregulatory element relative to the genomic single-copy gene *PTBP2*. (E) MFI of TCR^+^ populations in flow cytometry from four independent transductions normalized to mean of each experiment. Square boxes below TCRs indicate peptide specificities.

First, we sought to isolate HBV-specific CD4^+^ T cells and to identify their MHC II-restricted TCR sequences. Peripheral blood mononuclear cells (PBMCs) of donors with acute or resolved HBV infection (Figure 1A) were stimulated with peptides from HBV core (C), envelope (preS/S), and polymerase (P) proteins (Figure 1B), based on a literature review of known CD4^+^ T cell epitopes, as well as *in silico* prediction for human leukocyte antigen (HLA)-DR1 and HLA-DR13 (Table S1). After two weeks, tumor necrosis factor (TNF)-α, and/or IFN-γ-secreting CD4^+^ T cells were isolated by flow cytometry cell sorting and clonally expanded through limiting dilution cloning. Specificities comprised three core-derived peptides (C61, C91, C113), four envelope-derived peptides (preS9, S17, S21, and S36), and one polymerase-derived peptide (P774). Six peptides or overlapping parts of them had been described previously, and two peptides, preS9 and C91, were newly identified to be immunogenic (Table S1). In total, 20 TCRs with functional pairs of α and β chains were identified (Figure 1C). Next, to be able to express and characterize our MHC II-restricted HBV-specific TCRs in T cells, the respective TCR sequences were cloned into a retroviral vector, using codon-optimized variable α and β chain domains combined with murine constant domains (Figure 1B). Having established stable producer cell lines and a robust transduction protocol that yielded high but non-toxic transduction rates (Figure S1), we set out to characterize our panel of HBV-specific, MHC II-restricted TCRs in depth. First, we addressed the TCR expression level, which can be described with regard to the number of transduced cells in relation to the number of integrates as determined by quantitative polymerase chain reaction (qPCR), or the mean fluorescence intensity (MFI) in flow cytometry. With transduction rates ranging from approximately 60% to 90%, our cell batches featured an average of fewer than five integrated vector copies per cell (Figure 1D). Regarding the MFI of the TCR^+^ population in flow cytometry, we found distinct levels for each TCR across four independent transductions, with, e.g., TCR 2H12_S36_ displaying a relatively high and 1D12_C113_ a consistently low MFI (Figure 1E). Overall, we were able to reproducibly generate HBV-specific T cells of good quality, i.e., high TCR expression despite low integrate number, which is an important safety aspect for the use of transduced T cells in adoptive T cell therapy.

### TCRs recognize HBV peptides presented on nine different MHC II molecules

**Figure 2:**
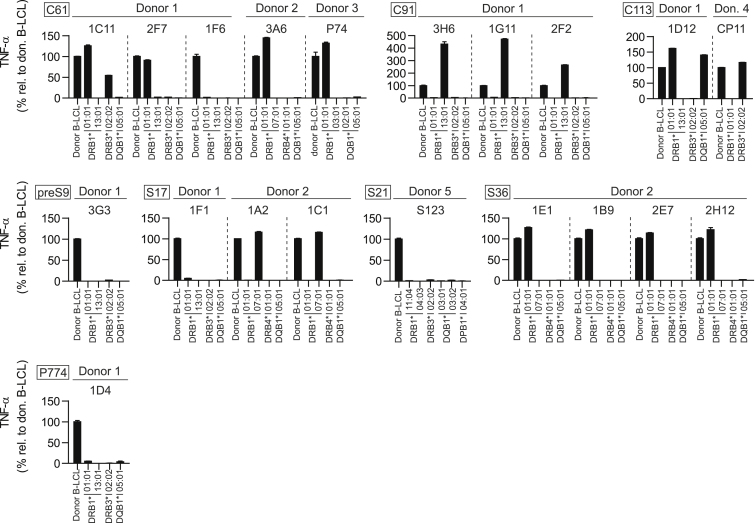
Verification of MHC II restrictions of HBV-specific TCRs TCR-transduced CD4^+^ T cells were co-cultured at an effector to target cell ratio of 2:1 with MHC II knockout (KO) fibroblasts or Raji-derived B-LCLs, stably transfected to express a single MHC II molecule and pulsed with 1 μM of target peptide. Each TCR was matched with target cells (limited to availability) co-expressing the corresponding MHC α and β chains of the respective donor; single MHC II transfectant cells are named after their respective MHC II β chain allele. TNF-α secretion was determined via ELISA and is shown relative to values from co-culture with the original B-LCLs of the respective donor. Data points represent mean values ±SD from triplicates. Controls without peptide were consistently below 5%, with the exception of TCR 2F2_C91_, which showed similarly high TNF-α secretion during co-culture with HLA-DRB1∗13:01 target cells with or without peptide (data not shown). Square boxes at the top left of each graph indicate peptide specificities.

For TCRs to be used for research or clinical applications, it is a prerequisite to know the MHC molecule by which each TCR is restricted. The restriction of our TCRs from CD4^+^ T cells was pre-defined by the MHC II alleles of the respective donors who were initially used for the isolation procedure (Figure 1A). The MHC restriction was primarily identified by co-culturing peptide-pulsed single MHC II transfectant target cells^31^ (limited to availability) with TCR-transduced T cells and measuring their TNF-α secretion (Figure 2). Based on this approach, the MHC II restriction was determined for most TCRs: all four S36-specific TCRs and C61-specific TCRs 1C11_C61_, 2F7_C61_, 3A6_C61_, and P74_C61_ were HLA-DRB1∗01:01-restricted. Interestingly, a promiscuous but specific binding behavior of TCR 1C11_C61_ toward DRB3∗02:02 in the presence of the target peptide was noticed. All three C91-specific TCRs were DRB1∗13:01-restricted. TCR 1D12_C113_ was both DRB1∗01:01- and DQA1∗01:01/DQB1∗05:01-restricted, whereas CP11_C113_ was DRB3∗02:02-restricted. TCRs 1A2_S17_ and 1C1_S17_ were DRB1∗07:01-restricted. In previous studies, peptides C61 and C113 had been associated with DRB1∗01:01^20^^,^^32^^,^^33^ and hence, we considered DRB1∗01:01 to be the main restriction of TCRs 1C11_C61_ and 1D12_C113_.

Since the available single MHCII-transfectant target cells did not cover all donor MHC II molecules, the restriction of TCRs 1F6_C61_, 3G3_preS9_, 1F1_S17_, S123_S21_, and 1D4_P774_ remained unclear. T cells transduced with these TCRs were therefore additionally co-cultured with a panel of partially HLA-matched B-LCLs (Figure S2, Table S2). A shared MHC allele between the respective donor and other cytokine-inducing B-LCLs suggested the specific MHC restriction. With this method, TCR 1F6_C61_ and TCR 3G3_preS9_ were found to be DQA1∗01:01/DQB1∗06:03-restricted; 3G3_preS9_, however, showed additional unspecific cytokine secretion (i.e., both in the presence and absence of peptide) in co-culture with a variety of B-LCLs that expressed the 01, 02, and 04 subtypes of the DRB1∗11 allele family. To confirm this unspecific cross-reactivity, further experiments, e.g., with single MHC II transfectant target cells, would be needed. The MHC II restrictions for TCR 1F1_S17_, S123_S21_, and 1D4_P774_ were identified to be DPA1∗01:03/DPB1∗02:01, DPA1∗01:03/DPB1∗15:01, and DPA1∗01:03/DPB1∗04:01, respectively.

In total, nine MHC II restrictions were confirmed (Table S3), thereby covering a broad range of MHC haplotypes worldwide. TCRs with HLA-DR1 and -DP4 restriction are particularly interesting for research purposes of T cell therapy against HBV infection, as HLA-A2/DR1 and -A2/DP4 double-transgenic mouse models are available.^34^^,^^35^

### MHC II-restricted TCR-transduced T cells recognize processed HBV antigen

**Figure 3:**
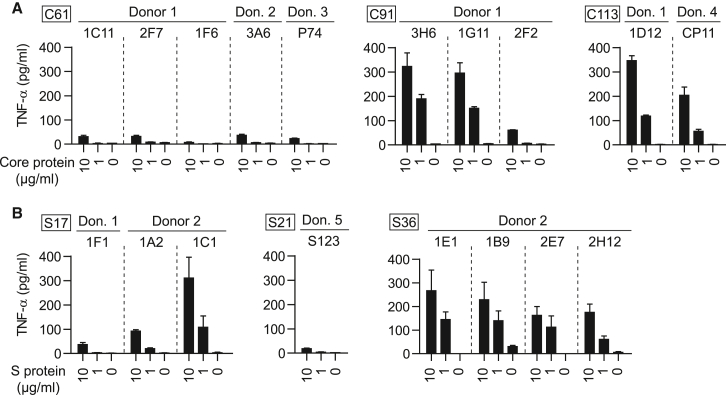
Recognition of physiologically processed HBV epitopes TCR-transduced CD4^+^ T cells were co-cultured at an effector to target cell ratio of 2:1 with HLA-matched B-LCLs that had been pre-incubated for 4 h with 10 or 1 μg/mL of core protein (A) or small (S) envelope protein (B). TNF-α secretion was determined after 16 h of co-culture via ELISA. Data points represent mean values ±SD from triplicates. Square boxes at the top left of each graph indicate peptide specificities.

Next, we asked whether the TCRs would recognize not only externally loaded peptide but also physiological epitopes, which requires antigen uptake, processing, and loading on MHC II. Accordingly, donor-derived B-LCLs were pre-incubated with native HBV core or small envelope protein, followed by co-culture with TCR-transduced T cells. Both antigens were taken up, processed intracellularly, and all corresponding HBV epitopes (C61, C91, C113, S17, S21, and S36) were presented to TCR-transduced T cells as indicated by their dose-dependent activation (Figure 3). TNF-α secretion in the absence of protein was below 5 pg/mL for all TCRs, with the exception of TCR 1B9_S36_, possibly related to a slight unspecific binding of TCR 1B9_S36_ or a minor background activation of T cells transduced with TCR 1B9_S36_. Of note, recognition of C61 was on average 10-fold lower than that of the other two core epitopes, C91 and C113, despite the fact that these two peptides had a lower binding affinity to the corresponding restricting MHC molecule (Table S3). This overall poorer response could argue for a diminished intracellular processing of the C61 peptide. Due to a lack of availability of the HBV large envelope and polymerase proteins, TCRs 3G3_preS9_ and 1D4_P774_ could not be included in this assay. Hence, one can only speculate that these TCRs similarly recognize processed antigen since they had been selected from a T cell repertoire primed by natural HBV infection.

### HBV-specific TCRs recognize peptides from different HBV genotypes

The interaction of the TCR with the pMHC complex is also influenced by variances in the peptide sequences. Therefore, we determined the recognition of different HBV genotypes by each TCR, which is considered favorable as it gives TCRs a broader range of therapeutic applicability. Out of the different HBV genotypes, genotype A-derived peptides had been used for initial T cell stimulation. The amino acid sequences of all eight epitopes are given in Table 1 for HBV genotypes A, B, C, and D, covering 79% of the worldwide HBV infections.^36^ Across these four genotypes, HBV core and envelope epitopes C61, C91, C113, preS9, S17, S21, and S36 vary in their respective amino acid sequences, whereas the polymerase epitope P774 is fully conserved (Table 1).

**Table 1:** Amino acid sequences of core, envelope, and polymerase peptides from HBV genotypes A, B, C, and D

Peptide	Genotype			
	A	B	C	D
C61	WGELMTLATWVGNNLEDP	WGELMNLATWVGSNLEDP		WGELMTLATWVGGNLEDP
C91	TNMGLKIRQLLWFHISCL	VNMGLKIRQLLWFHISCL		TNMGLKFRQLLWFHISCL
C113	ETVLEYLVSFGVWIRTPP			ETVIEYLVSFGVWIRTPP
preS9	RKGMGTNLSVPNPLGFFP		RQGMGTNLSVPNPLGFFP	- - - MGQNLSTSNPLGFFP
S17	AGFFLLTRILTIPQSLDS	AGFFLLTKILTIPQSLDS	AGFFLLTRILTIPQSLDS	
S21	LLTRILTIPQSLDSW	LLTKILTIPQSLDSW	LLTRILTIPQSLDSW	
S36	WTSLNFLGGSPVCLGQNS	WTSLNFLGGTPVCLGQNS	WTSLNFLGGAPTCPGQNS	WTSLNFLGGTTVCLGQNS
P774	LRGTSFVYVPSALNPADD			

Genotype A peptides were used for isolation of TCRs, since donor 1 had been formerly diagnosed with HBV genotype A infection. Amino acid differences in comparison with genotype A are underlined. Peptide specificities from HBV core (C), envelope (preS/S), or polymerase (P) proteins are given with a number indicating the peptide starting residue within the respective antigen.

**Figure 4:**
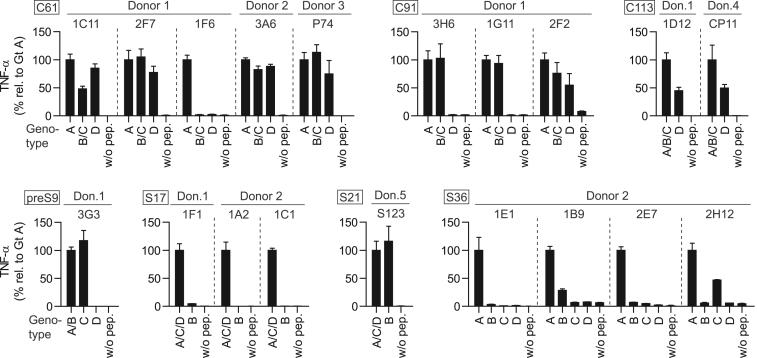
Recognition of peptide variants of major HBV genotypes TCR-transduced CD4^+^ T cells were co-cultured at an effector to target cell ratio of 2:1 with HLA-matched B-LCLs pulsed with 1 μM of peptide from HBV genotypes A, B, C, and D. TNF-α secretion was determined via ELISA after 16 h of co-culture and is shown relative to values from co-culture with genotype A (Gt A), since Gt A peptides had been used for initial T cell stimulation and isolation of HBV-specific CD4^+^ T cell clones. Co-cultures without peptide (w/o pep.) served as negative control. Data points represent mean values ±SD from triplicates. Square boxes at the top left of each graph indicate peptide specificities. TCR 1D4_P774_ was not included in this assay, since the P774 peptide is conserved across all four genotypes.

To evaluate recognition of HBV genotype B, C, and D homologous peptides (Table 1), TCR-transduced T cells were co-cultured with HLA-matched B-LCLs pulsed with the respective peptides from (Figure 4). All HLA-DRB1∗01:01-restricted C61-specific TCRs recognized all HBV genotypes, represented by the three different C61 variants. Interestingly, the DQA1∗01:01/DQB1∗06:03-restricted TCR 1F6_C61_ was only activated upon interaction with genotype A. C91-specific TCRs 3H6_C91_ and 1G11_C91_ detected both the A and B/C variant, whereas TCR 2F2_C91_ additionally bound to genotype D. The C113-specific TCRs recognized both epitope variants, thereby covering all four genotypes. The preS9-specific TCR 3G3_preS9_ was unable to recognize genotype D, which seems plausible given the major amino acid deletion in comparison with genotype A. TCR S123_S21_ recognized all four genotypes, with A, C, and D being sequence identical and B differing by one amino acid exchange. Interestingly, this amino acid exchange seemed relevant for TCRs 1F1_S17_, 1A2_S17_, and 1C1_S17_, which were derived from other donors and only bound the A/C/D epitope variant. S36-specific TCRs interacted mostly with genotype A; however, 1B9_S36_ and 2H12_S36_ displayed minor binding toward genotypes B and C, respectively. In total, nine TCRs recognized several HBV genotypes and could thus be attributed a higher therapeutic range than TCRs that only recognize a single genotypic variant.

### MHC II-restricted TCRs recognize nanomolar peptide concentrations

**Figure 5:**
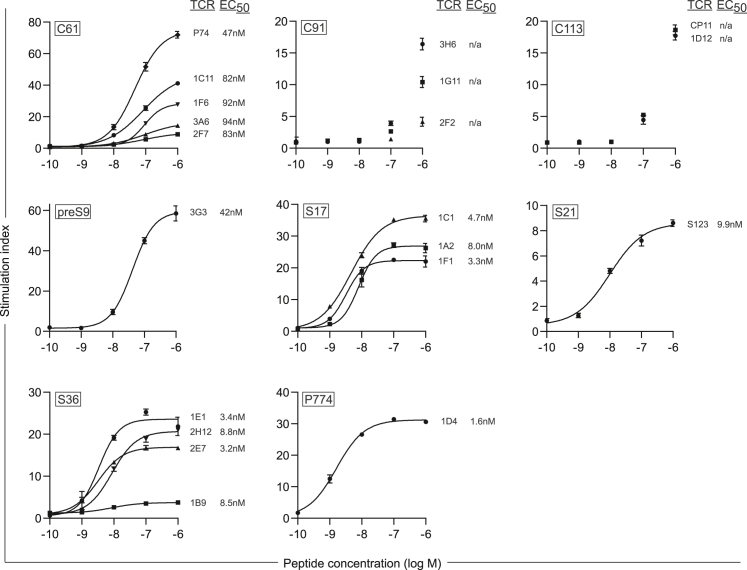
Functional avidity of MHC II-restricted TCRs determined with proliferation assay TCR-transduced CD4^+^ T cells were co-cultured at an effector to target cell ratio of 2:1 with HLA-matched B-LCLs titrating the amount of target peptide from 1 μM to 100 pM. Proliferation was assessed through integration of ^3^H-thymidine after 72 h of co-culture. Results are expressed as stimulation index, i.e., cpm of a stimulated sample divided by cpm of the unloaded control. Data points represent mean values ±SD from triplicates. All indicated EC_50_ values were calculated with a non-linear dose-response ordinary fit. EC_50_ values for C91- and C113-specific TCRs could not be calculated (n/a) because they did not reach a plateau of proliferation at the highest peptide concentration. Square boxes at the top left of each graph indicate peptide specificities.

To further characterize the set of HBV-specific TCRs, we analyzed their sensitivity and functional avidity. This was indirectly measured via their potential to induce T cell proliferation and determining the peptide concentration that induced the half maximum (half-maximal effective concentration [EC_50_]) proliferation capacity (Figure 5). All TCRs specific for epitopes S17, S21, S36, and P774, or C61 and preS9, showed EC_50_ values in a one-digit or two-digit nanomolar range, respectively. EC_50_ values of C91- and C113-specific TCRs could not be calculated and are expected to exceed the values of the TCRs above. In summary, most of the TCRs conveyed a high functional avidity with values typical for TCRs recognizing virus, i.e., foreign antigen.^37^

### CD4^+^ and CD8^+^ T cells transduced with MHC II-restricted TCRs are polyfunctional

**Figure 6:**
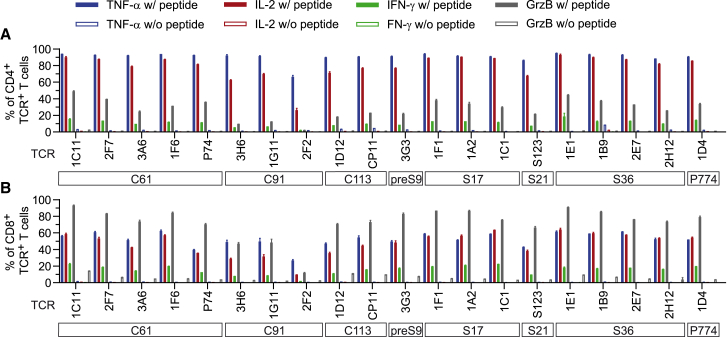
Cytokine and GrzB secretion of TCR-transduced CD4^+^ or CD8^+^ T cells CD4^+^ and CD8^+^ T cells were co-transduced and then separated by positive selection through magnetic-activated cell sorting prior to the experiment with purities ≥98%. TCR-transduced CD4^+^ (A) or CD8^+^ (B) T cells were co-cultured with HLA-matched B-LCLs, then pulsed with 1 μM of target peptide (w/ peptide). Brefeldin A was added 1 h after co-culture start to retain cytokines in the endoplasmic reticulum (ER). TNF-α (blue bars),IL-2 (red bars), IFN-γ (green bars) and GrzB (gray bars) were measured via intracellular cytokine staining and flow cytometry after 14 h of co-culture in CD4^+^ TCR^+^ or CD8^+^ TCR^+^ subsets, respectively. Co-cultures without peptide (w/o peptide) served as negative control (empty bars in respective colors). Data points represent mean values ±SD from triplicates. Square boxes below TCRs indicate peptide specificities.

Next, we asked which functional profile MHC II-restricted TCRs would convey when transduced into either CD4^+^ or CD8^+^ T cells. Therefore, the two T cell populations were purified after TCR engraftment and employed separately in co-cultures with peptide-loaded target cells. CD4^+^ T cells (Figure 6A) generally secreted high amounts of TNF-α and IL-2, with most TCRs inducing TNF-α in >81% and IL-2 in >74% of CD4^+^ TCR^+^ T cells. C91-specific TCRs, especially TCR 2F2_C91_, showed a slightly reduced cytokine secretion (Figure 6A), which correlated with the lower functional avidity of this TCR observed before (Figure 5). IFN-γ secretion was relatively low and occurred only in around 15% of all CD4^+^ TCR^+^ T cells (Figure 6A). Interestingly, most TCRs also induced granzyme B (GrzB) secretion, a serine protease associated with cytotoxic activity, in up to 50% of CD4^+^ TCR^+^ cells.

In line with their designated function, CD8^+^ T cells expressing MHC II-restricted TCRs had a predominantly cytotoxic profile as they secreted vast amounts of GrzB in 65%–90% of TCR^+^ cells for most TCRs (Figure 6B). They also secreted IL-2 and TNF-α in up to 60% of CD8^+^ TCR^+^ T cells. The fraction of IFN-γ producing cells with around 20% was slightly higher in CD8^+^ compared with CD4^+^ T cells. Again, all C91-specific TCRs, especially TCR 2F2_C91_, induced slightly less cytokines and GrzB in CD8^+^ T cells compared with other TCRs. In summary, expression in CD8^+^ T cells revealed that MHC II-restricted TCRs did not require CD4 co-receptor binding. In addition, activation of transduced CD4^+^ T cells induced a polyfunctional profile pointing at a T_H_1 phenotype.

### TCR-transduced CD4^+^ and CD8^+^ T cells are capable of killing MHC II-matched fibroblasts

**Figure 7:**
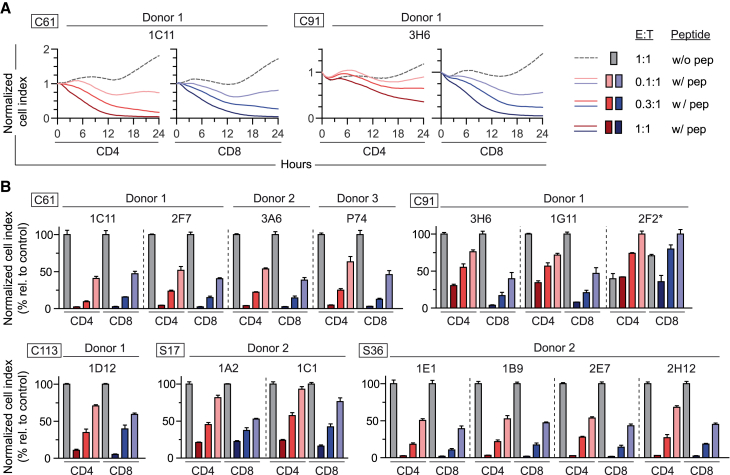
Cytotoxic capacity of TCR-transduced CD4^+^ and CD8^+^ T cells CD4^+^ and CD8^+^ T cells were co-transduced and then separated by positive selection through magnetic-activated cell sorting to purities of ≥98%. TCR-transduced CD4^+^ (red) or CD8^+^ (blue) T cells were co-cultured for 24 h with single MHC II transfectant fibroblasts pulsed with 1 μM of peptide (w/ pep) at an effector to target (E:T) cell ratio of 1:1 (dark color), 0.3:1 (medium color), or 0.1:1 (light color) or without peptide (w/o pep) at E:T ratio 1:1 (gray). Cytotoxicity was assessed via the adherence of target cells measured through electrical impedance and is given as a cell index normalized to the starting point of each co-culture. Considering the technical requirements of this assay, only TCRs were included, for which adherent single MHC II transfectant fibroblasts were available. (A) Cytotoxicity kinetics for exemplary TCRs 1C11_C61_ and 3H6_C91_. Data points were acquired every 30 min and represent mean values from triplicates. (B) Endpoint cytotoxicity after 24 h of co-culture for exemplary TCRs. The normalized cell index is given relative to killing of target cells without peptide at the highest E:T of 1:1, with the exception of TCR 2F2_C91_(∗), where samples with peptide at an E:T of 0.1:1 are set to 100%. Data points represent mean values ±SD from triplicates. Square boxes at the top left of each graph indicate peptide specificities.

In view of the strong GrzB secretion observed for most TCRs in both CD8^+^ and CD4^+^ T cells, the cytotoxic capacity of both T cell subsets was analyzed. To this end, peptide-loaded single MHC II transfectant fibroblasts served as target cells in a real-time cytotoxicity assay. In general, MHC II-restricted CD4^+^ or CD8^+^ T cells were able to recognize and kill peptide-loaded target cells within 24 h (Figure S3). Some TCRs conveyed distinct cytotoxic profiles depending on their expression in either CD4^+^ or CD8^+^ T cells, as shown in Figure 7A. While both TCR 1C11_C61_ CD4^+^ and CD8^+^ T cells had killed all target cells after around 12 h, killing of TCR 3H6_C91_ CD4^+^ T cells was delayed and only reached 50% compared with CD8^+^ T cells (Figure 7A). This slower killing kinetics of CD4^+^ T cells and reduced cytotoxicity after 24 h was mostly observed for C91-specific TCRs, possibly due to their lower functional avidity (Figures S3 and 7B). Unexpectedly, at the highest effector to target ratio of 1:1, TCR 2F2_C91_ showed unspecific killing of the control without peptide (Figures S3 and 7B). Since this unspecific activation was uniquely seen upon co-culture with single MHC II transfectant fibroblasts and never on donor-derived B-LCL, it may be related to cross-reactivity with a fibroblast-derived peptide, potentially enhanced by the artificial over-expression of HLA-DR13 on the single MHC II transfectant target cell line. Overall, nine out of the 14 tested TCRs had a functional avidity high enough to result in prominent killing by CD4^+^ as well as CD8^+^ T cells.

## Discussion

CD4^+^ T cells are needed for an efficient and long-lasting antiviral immune response by providing help to CD8^+^ T cells and inducing B cell differentiation. In this study, we isolated and characterized a panel of MHC II-restricted, HBV-specific TCRs, which can be used to complement MHC I-restricted T cells in T cell therapy or as an experimental tool to study HBV-specific immunity.

Twenty MHC II-restricted TCRs (Table S4) specific for eight peptides derived from the HBV core, envelope, and polymerase proteins could be isolated from donors with acute or resolved HBV infection. Interestingly, both epitopes newly identified to be immunogenic, i.e., C91 and preS9, had not stood out by their prediction score, and TCR 3G3_preS9_ was actually restricted toward an MHC II molecule other than those used for initial peptide prediction. The epitope preS9 is particularly intriguing, since CD4^+^ T cells redirected with the receptor 3G3_preS9_ would be expected to prime B cells specific for the large envelope protein, which could give rise to entry-neutralizing antibodies.^38^^,^^39^ Fourteen of the TCRs recognized more than one HBV genotype variant, making them therapeutically more widely applicable. The failure of, e.g., S36-specific TCRs in recognizing genotypes other than A could be caused by changes in the amino acids affecting the binding core and exposing different residues to the CDR3 regions of the TCR. To elucidate the importance of each amino acid, alanine scans in combination with *in silico* 3D modelling of the peptide:MHC-TCR interaction are warranted.

Besides the binding specificity and strength as defined by each TCR's unique CDR3 region, functionality of a TCR is also influenced by binding and presentation of the respective peptide on the MHC molecule. To address all of these points, T cells expressing MHC II-restricted, HBV-specific TCRs were generated by retroviral transduction. The levels of expression in terms of MFI were consistent for each TCR across several independent transduction experiments and did not correlate with the number of integrates. This suggests that the maximum expression level is a phenotypic feature inherent to each TCR and does not depend on the transduction efficiency. This observation concurs with a recent study classifying TCRs into weak and dominant phenotypes according to their MFI as a measure of surface expression.^40^ Thomas *et al*. showed that the variable β-chain TRBV7-9 was over-represented in TCRs with a weak expression phenotype, which holds true for our TCR with the lowest MFI of all, TCR 1D12_C113_. Another characteristic of a TCR is its functional avidity, which depends on the affinity of the TCR variable regions to the pMHC complex and correlates with the strength of the T cell response.^41^ Virus-specific TCRs are typically of high affinity, since they recognize foreign antigen in a *de novo* encounter and have hence eluded the negative selection process during thymic development.^37^ Accordingly, in this study, high values of functional avidity were determined for 15 TCRs with EC_50_ values in a low nanomolar range. Numerous studies in murine models and humans have gathered evidence that CD8^+^ T cells of higher functional avidity are more efficient in clearing viral infection.^42^, ^43^, ^44^ For CD4^+^ T cells in particular, fewer conclusive data regarding TCR affinity are available, and it has been suggested that MHC II-restricted TCRs generally display weaker binding affinities in comparison with MHC I-restricted TCRs.^45^^,^^46^ Hence, with our 20 TCRs covering a range of functional avidities and specificities, we here provide a highly useful resource for studying MHC II-restricted TCR affinities in more detail.

Finally, TCR binding depends on encountering the peptide on the correct MHC molecule. Nine MHC II restrictions were identified during this work, five of which have been linked to beneficial effects with regard to HBV infection, underlining their potential for immunotherapy. For example, studies have shown a correlation with protection against HBV for HLA-DRB1∗01:01,^47^ DQB1∗06:03,^48^ DPB1∗02:01,^49^ and DRB1∗13:01.^50^, ^51^, ^52^, ^53^ In addition, the latter has repeatedly been associated with resolution of HBV infection worldwide.^54^, ^55^, ^56^ HLA-DPB1∗15:01, in turn, has been linked to spontaneous HBsAg seroconversion in HBV-infected individuals.^57^

For application of TCRs in T cell therapy, an important aspect to consider is the geographic distribution of MHC molecules as well as the local prevalence of HBV infection. Out of our isolated TCRs, the HLA-DPB1∗02:01-restricted ones would have the broadest therapeutic applicability, given this allele is common with, e.g., a frequency of 23% in China's Han population.^58^ Interestingly, TCR 1C11_C61_ showed a promiscuous but specific binding behavior toward DRB1∗01:01 and DRB3∗02:02. Such TCR promiscuity has been reported previously in the form of a single target peptide being recognized on different MHC molecules,^59^^,^^60^ and can potentially broaden the number of patients eligible for T cell therapy with a given receptor. The number of different MHC restrictions covered by our library is also advantageous for further studies of, e.g., immune cell interactions and immunomodulators that supposedly increase MHC presentation, or in pre-clinical models of HBV infection and HBV-targeted T cell therapy. As such, our TCRs could be used with MHC II-expressing HBV-susceptible cell lines (e.g., HepaRG, DRB1∗07:01^+^, DPB1∗04:01^+^), “professional” APC lines that take up HBV (e.g., THP-1,^61^ DRB1∗01:01^+^, DQB1∗05:01^+^, DPB1∗02:01^+^), or *in vivo* in AAV-HBV infected mice featuring HLA-A2 and -DR1^34^ or -DP4^35^ expression.

T cell signaling after engagement of pMHC and TCR is supported by CD4 or CD8 co-receptor binding to the respective MHC molecule.^62^ All the MHC II-restricted TCRs characterized throughout this study were equally able to activate CD8^+^ T cells regardless of their different functional avidities. This argues for a minor role of CD4 regarding the actual TCR-pMHC interaction,^63^^,^^64^ but rather a role for enhancing T cell sensitivity via its strong intracellular association with tyrosine kinases in CD4^+^ T cells.^65^^,^^66^ The introduction of MHC II-restricted TCRs in CD8^+^ T cells has rarely been attempted. One group compared CD4^+^ and CD8^+^ T cells transduced with an HLA-DQ5-restricted TCR targeting the dead box RNA helicase Y *in vitro*. They reported similar killing capacity of both transduced CD4^+^ and CD8^+^ T cells, but cytokine secretion was significantly diminished in CD8^+^ T cells and only slightly increased after co-introduction of the CD4 co-receptor.^67^ Ample opportunity remains to further investigate the nature and potential of MHC II-restricted CD8^+^ T cells based on the panel of TCRs presented in this study.

Both CD4^+^ and CD8^+^ T cells transduced with MHC II-restricted TCRs were polyfunctional and produced varying amounts of cytokines, such as TNF-α, IFN-γ, and IL-2. Thus, CD4^+^ T cells showed rather a T_H_1-phenotype,^68^ most likely induced by the anti-CD3/anti-CD28 and IL-2 stimulation mimicking antigen encounter during the transduction procedure. IFN-γ and TNF-α were shown to interfere with the stability of HBV covalently closed circular DNA (cccDNA) via nuclear deaminases.^69^ This cytokine-mediated HBV inhibition was also observed upon the addition of HBV-specific redirected T cells without direct cell-cell contact.^69^ Therefore, cytokine-secreting CD4^+^ T cells could also contribute directly to the antiviral effect of adoptive T cell therapy. Given that especially IFN-γ-producing S-specific CD4^+^ T cells but not TNF-α-producing CD4^+^ T cells were shown to correlate with HBV clearance in CHB patients,^19^ artificial generation of such cells with our TCRs would be particularly interesting.

Furthermore, CD4^+^ T cells transduced with MHC II-restricted TCRs secreted varying amounts of GrzB and selectively killed MHC II-expressing peptide-pulsed target cells. The percentage of GrzB^+^ CD4^+^ T cells was consistently lower compared with CD8^+^ T cells, which could be explained by CD8^+^ T cells as professional cytotoxic cells storing more GrzB intracellularly.^70^ The presence and characteristics of CD4^+^ cytotoxic T lymphocytes (CTLs) have been described for murine or human viral infection.^71^^,^^72^ Initially, they were believed to be an artifact of long-term *in vitro* cell culture but, over time, numerous studies also reported their existence *ex vivo*.^73^, ^74^, ^75^, ^76^ In humans, they were associated with a protective role in influenza^77^ and HIV infection.^78^ Little is known, however, with regard to their role in HBV infection. A study comparing individuals with chronic HBV, hepatitis C virus (HCV), or HBV/hepatitis D virus (HDV) (co-)infection with healthy controls showed elevated numbers of CD4^+^ T cells expressing perforin *ex vivo*, with particularly high rates in HBV/HDV co-infected patients.^79^ In addition, perforin expression was most pronounced in patients with advanced hepatitis and was linked to liver damage.^79^ This has led to the hypothesis that CD4^+^ CTLs in chronic hepatitis may in fact contribute to immunopathology.^71^ The TCRs described in our study could help elucidate the role of CD4^+^ CTLs in chronic hepatitis and HBV-induced HCC in more detail.

In this regard, it is important to define potential target cells expressing MHC II before applying cytotoxic HBV-specific T cells in adoptive T cell therapy. Human hepatocytes are not thought to express MHC II under normal conditions, and although some upregulation has been proposed to occur during inflammation,^80^ solid and contemporary data on MHC II expression during viral hepatitis are missing. In transgenic mice overexpressing the transcriptional regulator of MHC II, hepatocytes were shown to function as APCs, specifically activating CD4^+^ T cells.^81^ Professional APCs like dendritic cells are presumably protected from cytotoxic CD4^+^ CTL activity given that they express the GrzB inhibitor SerpinB9.^82^ In addition, upon activation by dendritic cells, T cells rapidly express cytotoxic T lymphocyte–associated protein 4 (CTLA-4), which then competes with CD28 for interaction with CD80 on the APC. This prevents the formation of an effective immunological synapse and ultimately protects the APC from becoming a T cell target.^83^ Liver-resident Kupffer cells and liver sinusoidal endothelial cells, which upregulate MHC II expression in response to pro-inflammatory cytokines such as IFN-γ, may well be protected by similar mechanisms given their antigen-presenting function and CD80 expression.^84^ It seems reasonable to assume that major damage to APCs in the liver is unlikely. Efficient targeting of MHC II-expressing hepatocytes by CD4^+^ CTLs would be unexpected and their direct antiviral activity remains to be determined. Especially in the treatment of HBV-induced HCC, their role may even be beneficial, as, for example, CD4^+^ T cells with cytotoxic activity were shown to induce tumor rejection in a melanoma model.^85^

Taken together, we here described a library of 20 MHC II-restricted TCRs specific for different HBV antigens. An in-depth characterization defined their MHC restriction, expression levels, recognition of different genotypes, and intracellularly processed antigen, as well as their functional avidity. With regard to these qualities, C61-specific TCRs (e.g., 1C11_C61_ and P74_C61_) could be considered favorable for their further evaluation regarding their potential in adoptive T cell therapy. S- and L-specific TCRs (e.g., 1F1_S17_ and 3G3_preS9_) will be especially interesting for the investigation of B cell responses and induction of HBV-neutralizing antibodies. This study lays the groundwork for the further use of MHC II-restricted TCRs in T cell therapy of chronic HBV infection and HBV-induced HCC and provides a valuable tool for the study of CD4^+^ T cells and their role in HBV infection and cure.

## Materials and methods

### T cell stimulation

PBMC from donors with resolved or acute HBV infection were isolated via a standard densitygradient (Biocoll, Merck). Informed consent in writing was obtained from each patient. PBMC from donor 1 and donor 2 were stimulated with 1 μM of single peptides (Peptides & Elephants or JPT Peptide Technologies, Table S1) for 14 days at 1 × 10^6^/well in a 24-well plate and expanded when necessary, and 10 ng/mL IL-7 and IL-15 (both from Peprotech) were added on day 0. 50 U/mL IL-2 (Proleukin, Novartis Pharmaceuticals) was added on day 1 and to fresh medium after expansion. PBMCs were kept in T cell medium with human serum: RPMI, 10% human serum (own production from male, healthy donors), 1% penicillin-streptomycin (pen/strep), 1% glutamine, 1% sodium pyruvate, 1% non-essential amino acids (NEAA), 10 mM HEPES, and 16.6 μg/mL gentamicin (all from Thermo Fisher Scientific). Minor adjustments were applied to the stimulation conditions for donors 3 to 5 as described in the supplemental methods.

### T cell cloning

T cells were restimulated with the respective peptide (1 μM) and stained with the TNF-α and/or IFN-γ secretion assay (Miltenyi Biotec) according to the manufacturer's instructions as well as anti-human CD4-APC (eBioscience, Thermo Fisher Scientific) and anti-human CD8-PB (BioLegend). TNF-α^+^ CD4^+^ T cells were enriched using a fluorescence-activated cell sorting (FACS) Aria III (BD) or a MoFlo XDP cell sorter (Beckmann Coulter), and 0.5 cells/well were seeded in 96-well round-bottom plates containing 7.5 × 10^4^ irradiated heterologous PBMCs (35 Gy), 1 × 10^4^ B-LCLs (50 Gy), 50 IU/mL IL-2, and 30 ng/mL OKT-3 antibody (eBioscience, Thermo Fisher Scientific). HBV-specific T cells clones were identified as described in the supplemental methods. For expansion, selected HBV-specific T cell clones were moved to a 12-well plate containing 5 × 10^6^ irradiated PBMCs, 1 × 10^6^ irradiated B-LCLs, and 30 ng/mL OKT-3 antibody. Then 50 U/mL IL-2 were supplemented on days 1, 5, 8, and 11 and split to two wells when necessary. The TCR chains of HBV-specific clones were analyzed and cloned as described in the supplemental methods.

### Retroviral transduction of T cells

T cells were enriched using human T activator CD3/CD28 Dynabeads (Thermo Fisher Scientific) and pre-stimulated for 2 days in T cell medium with FBS: RPMI, 10% FBS, 1% pen/strep, 1% glutamine, 1% sodium pyruvate, 1% NEAA, 10 mM HEPES, 16.6 μg/mL gentamicin (all from Thermo Fisher Scientific), supplemented with 300 U/mL IL-2. The 0.45 μm-filtered retrovirus cell culture supernatant from stable producer cell lines was centrifuged at 2,000 × *g*, 32°C for 2 h on non-tissue culture-treated plates (Corning) coated with 20 μg/mL RetroNectin for 2 h (Takara). Retrovirus cell culture supernatant was removed and T cells were spinoculated onto the retrovirus-coated plate at 1,000 × *g* for 10 min. A second transduction was performed after 24 h. TCR expression was determined by flow cytometry. Staining was done for 30 min on ice in the dark, using the primary antibodies anti-human CD4-APC , anti-human CD8-PB, and anti-mouse TCRβ-PE (BD Biosciences), diluted in PBS with 0.1% BSA (Sigma-Aldrich). Cells were analyzed using a CytoFLEX S (Beckman Coulter) and data were analyzed with FlowJo 10.4 software. To determine the number of integrates per cell, genomic DNA from transduced T cells was isolated with a DNA tissue extraction kit (MACHEREY-NAGEL). The vector copy number was measured in a multiplex qPCR of viral woodchuck hepatitis virus postregulatory element relative to the genomic *PTBP2* as described elsewhere.^86^ The protocol and plasmid standard were kindly provided by the Hannover Medical School, Institute of Experimental Hematology.

### Co-cultures with B-LCLs

B-LCLs were cultivated in RPMI full medium: RPMI with 10% FBS, 1% pen/strep, 1% glutamine, 1% sodium pyruvate, and 1% NEAA (all from Thermo Fisher Scientific). Prior to the experiment, B-LCLs were irradiated with 50 Gy and loaded with 1 μM or decreasing amounts of peptide for 2 h at 37°C and then washed twice with PBS. For recognition of physiologically processed antigen, B-LCLs were pre-incubated with the HBV core or small envelope protein (genotype A, kindly provided by the Centro de Ingeniería Genética y Biotecnología de Cuba) at 1 or 10 μg/mL. TCR^+^ T cells (1 × 10^5^) were incubated with 5 × 10^4^ peptide-loaded B-LCLs at 37°C. TNF-α secretion via ELISA (BD) was measured after 16 h from supernatants. For intracellular cytokine staining, 2.5 μg/mL Brefeldin A (Sigma-Aldrich) was added 1 h after co-culture start, and staining was performed after 14 h, using the Fixation/Permeabilization Solution kit (BD Biosciences) and the following antibodies: live/dead Fixable Aqua stain (Invitrogen, Thermo Fisher Scientific), anti-human CD4-PerCP (clone SK3, BioLegend), anti-human CD8-FITC (clone RPA-T8, Invitrogen, Thermo Fisher Scientific), anti-mouse TCRβ-PE (clone H57-597, BD Biosciences), anti-human IFN-γ-AF700 (clone B27, BD Biosciences), anti-human TNF-α-APC (clone Mab11, BioLegend), anti-human IL-2-PE-Cy7 (clone MQ1-17H12, Invitrogen, Thermo Fisher Scientific), and anti-human GrzB-PB (clone N4TL33, Invitrogen, Thermo Fisher Scientific).

For proliferation analysis, after 72 h of co-culture 1 μCi ^3^H-thymidine was added per well and incubated for another 16 h at 37°C. The cells were then transferred onto a Filtermat A membrane using a Filtermat-96 Harvester. After a drying period of 5 h at 37°C, the membranes were placed into plastic scintillation sleeves with approximately 1 mL of BetaPlate scintillation fluid. Counts per minute (cpm) were evaluated in a MicroBeta TriLux 1450 scintillation counter (all from PerkinElmer). EC_50_ values were calculated with a non-linear dose-response ordinary fit with Prism8 (GraphPad). R^2^ values were consistently ≥0.99 with the exception of TCR 1E1_S36_ (0.97).

### Co-culture with single MHC II transfectant target cells

Single MHC II transfectant target cells^31^ were kindly provided by Alessandro Sette, La Jolla Institute of Immunology, San Diego, United States. These DAP3-based fibroblasts or RM3-based B-lymphoblasts were grown in RPMI full medium and maintained under selection pressure with 200 μg/mL Geneticin or 700 μg/mL Geneticin and 12 μg/mL blasticidin (all from Thermo Fisher Scientific), respectively. To increase MHC expression prior to co-culture, single MHC II transfectants were stimulated with 100 μg/mL sodium butyrate (Sigma-Aldrich) overnight at 37°C in their respective culture media. DAP3-based adherent fibroblasts were seeded with 5 × 10^4^ cells/well in flat-bottom 96-well plates, loaded with 1 μM peptide for 4 h at 37°C and then washed twice with PBS. Raji-based suspension B-LCLs were loaded in V-bottom plates with 1 μM of peptide for 4 h at 37°C, washed twice with PBS, and subsequently seeded into round-bottom 96-well plates with 5 × 10^4^ cells/well. Transduced T cells were added with 1 × 10^5^ cells/well and incubated overnight at 37°C. After 16 h, supernatants were stored at -20°C and TNF-α secretion was measured by ELISA at a later time point (Invitrogen, Thermo Fisher Scientific).

### Real-time cytotoxicity measurement

DAP3-based fibroblasts were prepared for co-culture with T cells as described above, seeded onto 96-well electronic microtiter plates (ACEA Biosciences) with 5 × 10^4^/well, loaded with 1 μM of peptide for 4 h at 37°C, and washed twice with PBS. CD4^+^ or CD8^+^ TCR^+^ T cells were added at different effector to target ratios (1:1, 0.3:1, 0.1:1). The impedance, which reflects adherence of the target cells to the bottom of the plate, was measured every 30 min using an xCELLigence SP real-time cell analyzer (ACEA Biosciences).

### Study approval

The use of volunteer PBMCs was approved by the local ethics board of the University Hospital rechts der Isar, Munich, and the ethics committee of the University of LMU, Munich. Written informed consent was obtained from all blood donors.

## Supplemental information

Document S1. Supplemental methods, Figures S1–S3, and Tables S1–S4

Document S2. Article plus supplemental information
