# Supplementary material for: Characterization of a library of 20 HBV-specific MHC class II-restricted T cell receptors
Source: Mol Ther Methods Clin Dev. 2021 Oct 29;23:476–89. doi: 10.1016/j.omtm.2021.10.012 (PMC8605085; doi:10.1016/j.omtm.2021.10.012)
Supplement: Document S1. Supplemental methods, Figures S1–S3, and Tables S1–S4 [file mmc1.pdf]

OMTM, Volume *23*

Supplemental information

Characterization of a library of 20 HBV-specific MHC class II-restricted T cell receptors

Sophia Schreiber, Melanie Honz, Weeda Mamozai, Peter Kurktschiev, Matthias Schiemann, Klaus Witter, Eugene Moore, Christina Zielinski, Alessandro Sette, Ulrike Protzer, and Karin Wisskirchen

Supplemental figures

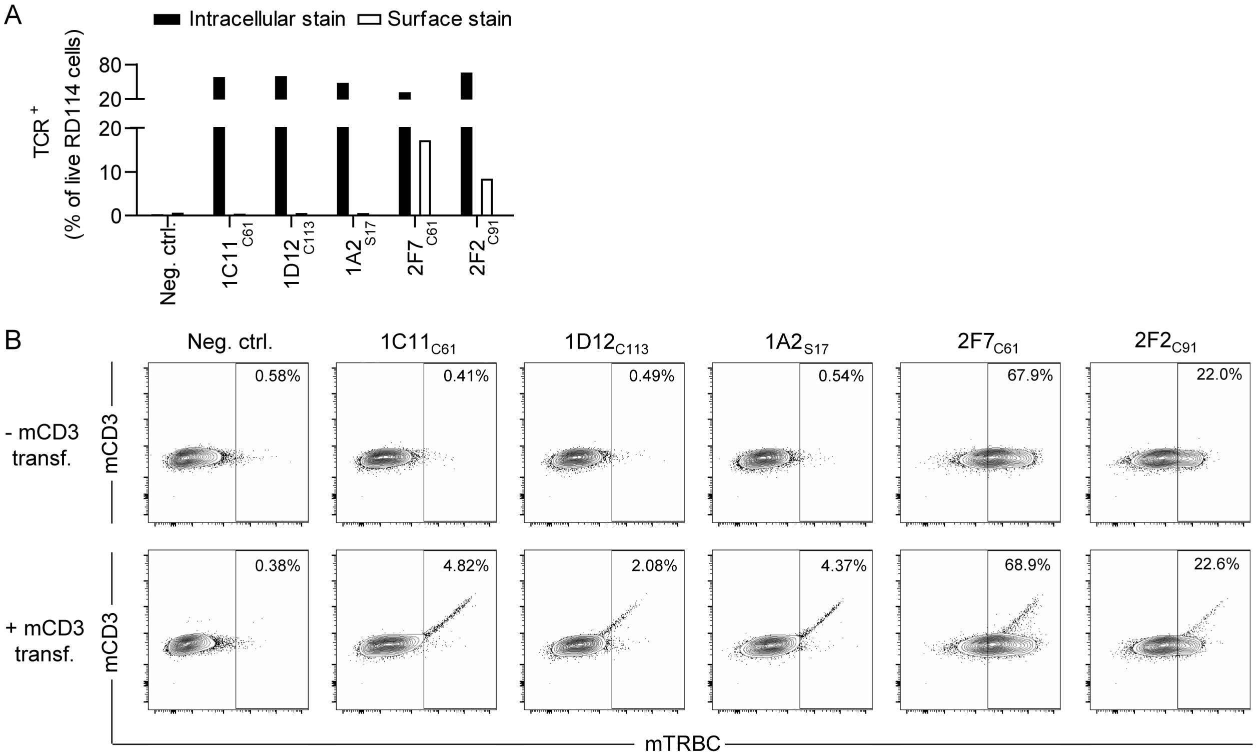

Figure S1: Generation of stable producer cell lines for the production of retroviral particles for transduction. Gibbon ape leukaemia virus (GALV) producer cells were transfected with the retroviral vector MP71 containing the respective transgenic TCR. The supernatant containing retroviral particles was used to transduce RD114 producer cells, resulting in a stable genomic integration of the TCR coding sequence. The resulting stably transduced RD114 cells were then enriched by flow cytometry cell sorting based on TCR surface expression. (A) Transduction rates of RD114 cells were determined by flow cytometry through intracellular (black bars) or surface staining (white bars) of the murine constant β-domain (mTRBC) of each TCR. The examples shown here include TCRs where surface staining was either possible or unsuccessful. (B) In producer cells showing little to no TCR surface expression, transient transfection (transf.) with the murine CD3 δγεζ-chains (mCD3) prior to enrichment increased and stabilized TCR surface expression sufficiently for cell sorting.

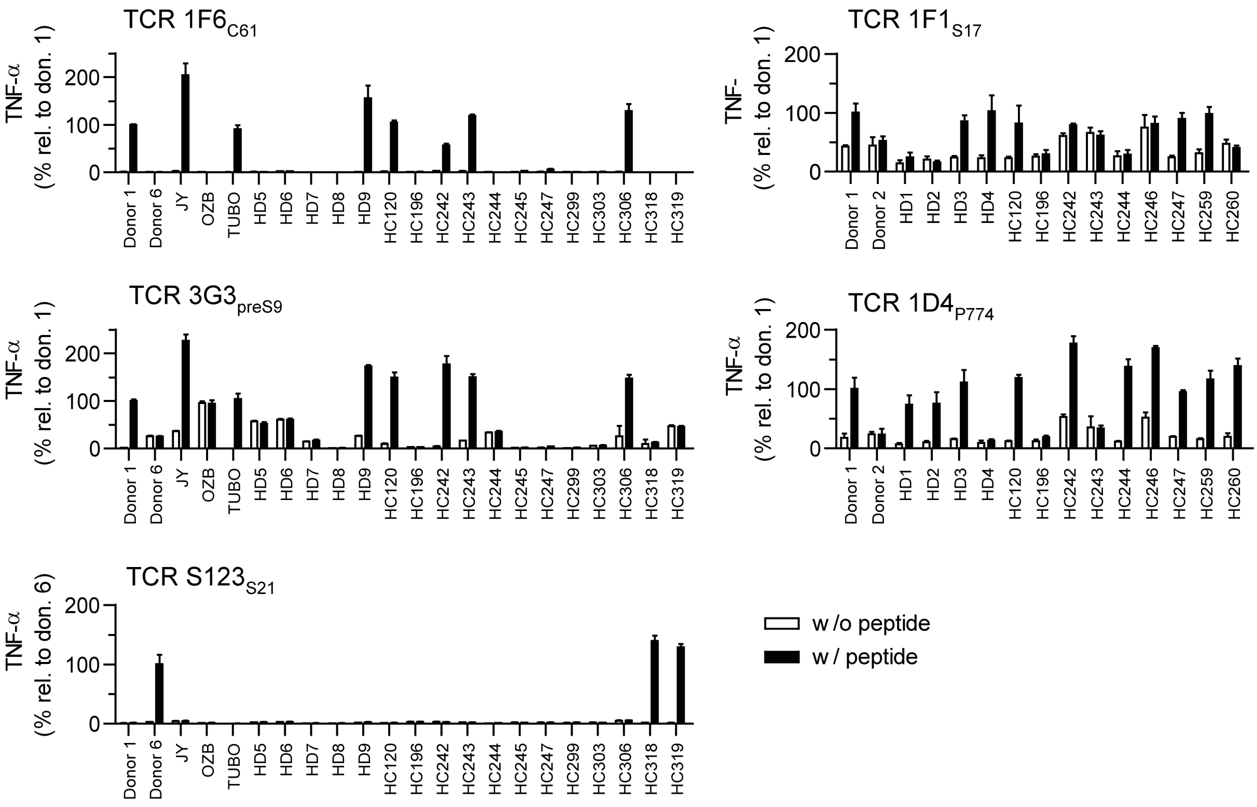

Figure S2: Co-culture of TCR-transduced T cells with partially HLA-matched B-LCLs to determine MHC class II restriction. T cells were transduced with TCRs 1F6$_{C61}$, 3G3$_{preS9}$, S123$_{S21}$, 1F1$_{S17}$ and 1D4$_{P774}$ and co-cultured at an E:T of 2:1 with partially HLA-matched B-LCLs, pulsed with 1 µM of the respective peptide (w/ peptide, black bars) or without peptide (w/o peptide, white bars). TNF-α secretion was determined via ELISA after 16 hours of co-culture and is shown relative to values from co-culture with donor B-LCLs. Data points represent mean values ± SD from triplicates. TCR clone names are indicated at the top left of each graph. The respective MHC II alleles of each B-LCL are summarized in Table S2.

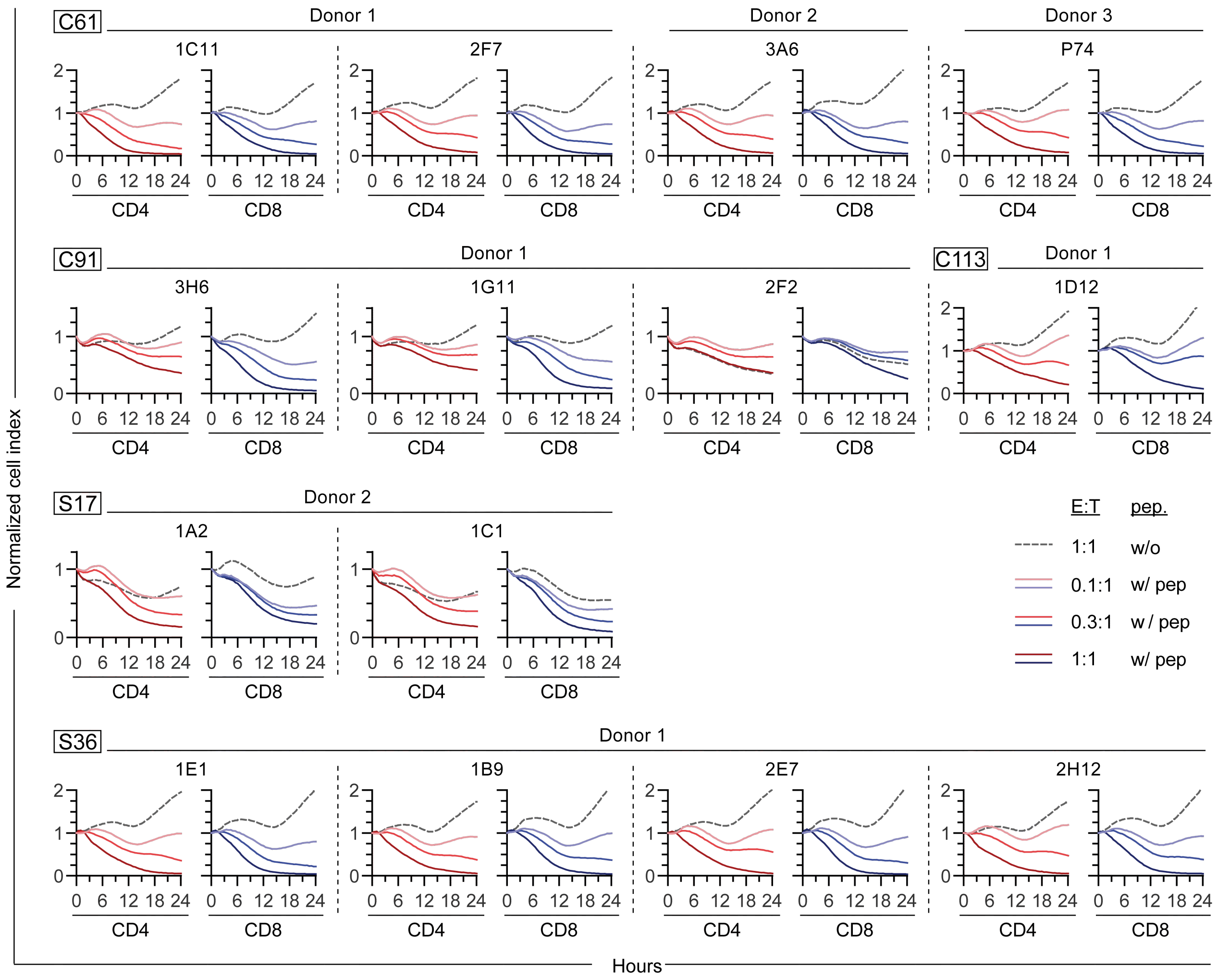

Figure S3: Kinetics of cytotoxicity of TCR-transduced CD4$^+$ and CD8$^+$ T cells. CD4$^+$ (red) or CD8$^+$ (blue) TCR-transduced T cells were co-cultured for 24 hours with single MHC II transfectant fibroblasts pulsed with 1 µM peptide (w/ pep) at different effector to target (E:T) cell ratios: 1:1 (dark color), 0.3:1 (medium color), or 0.1:1 (light color) or without peptide (w/o pep) at an E:T ratio of 1:1 (grey). Cytotoxicity was assessed via the adherence of target cells measured through electrical impedance and is given as a cell index normalized to the starting point of each co-culture. Considering the technical requirements of this assay, only TCRs were included, for which adherent single MHC II transfectant fibroblasts were available. Data points were acquired every 30 minutes for 24 hours and represent mean values from triplicates. Endpoint cytotoxicity data are shown in Figure 7. Square boxes at the top left of each graph indicate peptide specificities. Mock-transduced T cells served as an additional negative control and no killing was observed after 24 hours of co-culture (data not shown).

Supplemental tables

Table S1: Characteristics and sequences of peptides used for stimulation. Left side: Peptides from this study used for stimulation of PBMC of donors 1 and 2. Specificities for which TCRs were isolated are highlighted in bold. Binding affinities (IC$_{50}$) to HLA-DR1 and HLA-DR13 as predicted via the NetMHCIIpan 3.2 algorithm.[1] Predicted binding cores to DR1/DR13 are overlined/underlined in peptide sequence. IC$_{50}$ values <50 nM or <500 nM are considered strong or weak binders, respectively. Right side: Literature review with therein published epitopes, corresponding HLA alleles and references.[2-11] Amino acid overlaps with peptides from this study are double underlined. n.d.= none determined.

Peptides from this study					Literature review		
HBV protein	Peptide name	Peptide sequence (predicted binding cores of DR1 and DR13)	Predicted affinity IC$_{50}$ (nM) DR1	Predicted affinity IC$_{50}$ (nM) DR13	Published peptide (overlap with peptides from this study)	HLA	Reference
Core	C7	KEFGATVELLSFLPSDFF	493	1855	MDIDPYKEFGATVELLSFLP	DR	(2)
	C28	RDLLDTASALYREALESP	530	964			
	**C61**	WGELMTLATWVGNNLEDP	696	2048	LCWGELMTLATWVGVN	DR1	(3)
	C84	LVVNYVNTNMGLKIRQLL	156	65			
	**C91**	TNMGLKIRQLLWFHISCL	387	193			
	**C113**	ETVLEYLVSFGVWIRTPP	205	884	GRETVIEYLVSFGVW<br>EYLVSFGVWIRTPPA	DR1<br>DRw52/DR6	(4)<br>(2)
	C119	LVSFGVWIRTPPAYRPPN	104	277	VSFGVWIRTPPAYRPPNAPI	DR1	(2)
	C133	RPPNAPILSTLPETTVVR	801	2446			
Envelope	**preS9**	RKGMGTNLSVPNPLGFFP	704	2025			
	preS83	GILTTVSTIPPPASTNRQ	786	2276			
	preS116	HPQAMQWNSTAFHQALQD	573	780	MQWNSTTFHQTLQDPRVRGLYFPAGG<br>MQWNSTALHQALQDP	DR1<br>DR2	(5)<br>(6)
	preS134	PRVRGLYFPAGGSSSGTV	149	4167			
	S8	FLGPLLVLQAGFFLLTRI	502	1574			
	**S17**	AGFFLLTRILTIPQSLDS	130	395	FFLLTRILTIPQSLD<br>FFLLTRILTIPQSLD<br>QAGFFLLTRILTIPQS	DR2<br>DR7<br>DR1	(7)<br>(8)<br>(5)
	**S36**	WTSLNFLGGSPVCLGQNS	77	2344	TSLNFLGGSPVCLGQ	DR1	(5)
	S69	CPGYRWMCLRRFIIFLFI	363	242	PICPGYRWMCLRRFIIFL	DR12	(8)
	S93	FLLVLLDYQGMLPVCPLI	144	1106	FLLVLLDYQGMLP	DP4	(9)
	S158	FAKYLWEWASVRFSWLSL	117	450	WEWASARFSWLSL<br>WASVRFSW	DP4<br>DR11/14	(9)<br>(10)
	S165	WASVRFSWLSLLVPFVQW	165	656	SVRFSWLSLLVPFVQWF	DP2	(8)
	S179	FVQWFVGLSPTVWLSAIW	68	813	SLLVPFVQWFVGLSPTVWLSV<br>VGLSPTVWLSVI	DR1<br>DP4	(5)<br>(9)
	S199	WYWGPSLYSIVSPFIPLL	161	1164	YWGPSLYSIVSPFIPL	DR3	(8)
	S209	VSPFIPLLPIFFCLWVYI	410	2029			
Polymerase	P104	NEKRRLKLIMPARFYPTH	40	33			
	P412	PNLQSLTNLLSSNLSWLS	80	488	LQSLTNLLSSNLSWL	n.d.	(11)
	P454	SGLSRYVARLSSNSRIFN	61	92			
	P524	SPFLLAQFTSAICSVVRR	62	569			
	P573	TNFLLSLGIHLNPNKTKR	63	157			
	P636	QRIVGLLGFAAPFTQCGY	151	1112			
	P650	QCGYPALMPLYACIQSKQ	250	1532			
	**P774**	LRGTSFVYVPSALNPADD	22	908	AANWILRGTSFVYVP	n.d.	(11)
	P827	HLPVRVHFASPLHVAWRP	47	109			

Table S2: MHC restriction analysis for TCRs 1F6$_{C61}$, 3G3$_{preS9}$, S123$_{S21}$, 1F1$_{S17}$ and 1D4$_{P774}$ using partially HLA-matched B-LCLs. B-LCLs which induced cytokine secretion in TCR-transduced T cells solely in the presence of the corresponding peptide are labeled as "specific". B-LCLs which induced cytokine secretion both in the presence and absence of peptide and higher than the donor B-LCL unloaded control are labeled "unspecific". No cytokine secretion in the presence or absence of peptide is labeled "negative". MHC molecules shared exclusively among "specific" B-LCLs and the original donor's B-LCLs (underlined) are marked in grey for each TCR.

TCR 1F1$_{S17}$

	B-LCL	DRB1		DRB3		DRB4	DRB5	DQB1		DPB1		DQA1		DPA1	
Specific	Donor 1	01:01	13:01	02:02				05:01	06:03	02:01	04:01	01	01	01:03	
	HD3	07:01	15:01			01:03	01:01	02:02	05:01	02:01	04:01	01:02	02:01	01:03	
	HD4	07:01				01:03		03:03		02:01		02:01		01:03	
	HC120	13:01	13:02	01:01	03:01			06:03	06:04	03:01		01:02	01:03	01:03	
	HC247	15:01					01:01	06:02		02:01	04:02	01:02		01:03	
	HC259	04:01	08:02			01:03		03:02	04:02	02:01	04:01	03:01	04:01	01:03	
Negative	HD1	07:01				01:03		02:02		04:01	13:01	02:01		01:03	
	HD2	01:01	08:01					04:02	05:01	04:01	04:02	01:01	04:01	01:03	
	HC242	15:01					01:01	06:02	06:03	04:01		01:02		01:03	02:02
	HC244	03:01	07:01	01:01		01:03		02:01	02:02	04:01	10:01	02:01	01:03	01:03	02:01
	HC246	08:01						04:02		03:01	04:01	04:01	04:02	01:03	
	HC260	01:01	04:01			01:03		03:02	05:01	04:01	04:02	01:01	03:01	01:03	
	Donor 2	01:01	07:01			01:01		02:02	05:01	03:01	11:02	01	02:01	01:03	02:01
	HC243	13:01	13:02	01:01	03:01			06:03	06:04	03:01	11:01	01:02	01:03	01:03	02:01
	HC196	04:04				01:03		03:02	04:02	03:01	06:01	03:01	03:03	01:03	

TCR 1D4$_{P774}$

	B-LCL	DRB1		DRB3		DRB4	DRB5	DQB1		DPB1		DQA1		DPA1	
Specific	Donor 1	01:01	13:01	02:02				05:01	06:03	02:01	04:01	01	01	01:03	
	HD1	07:01				01:03		02:02		04:01	13:01	02:01		01:03	
	HD2	01:01	08:01					04:02	05:01	04:01	04:02	01:01	04:01	01:03	
	HD3	07:01	15:01			01:03	01:01	02:02	05:01	02:01	04:01	01:02	02:01	01:03	
	HC120	13:01	13:02	01:01	03:01			06:03	06:04	03:01		01:02	01:03	01:03	
	HC242	15:01					01:01	06:02	06:03	04:01		01:02		01:03	02:02
	HC244	03:01	07:01	01:01		01:03		02:01	02:02	04:01	10:01	02:01	01:03	01:03	02:01
	HC246	08:01						04:02		03:01	04:01	04:01	04:02	01:03	
	HC247	15:01					01:01	06:02		02:01	04:02	01:02		01:03	
	HC259	04:01	08:02			01:03		03:02	04:02	02:01	04:01	03:01	04:01	01:03	
	HC260	01:01	04:01			01:03		03:02	05:01	04:01	04:02	01:01	03:01	01:03	
Unspecific	Donor 2	01:01	07:01			01:01		02:02	05:01	03:01	11:02	01	02:01	01:03	02:01
	HC243	13:01	13:02	01:01	03:01			06:03	06:04	03:01	11:01	01:02	01:03	01:03	02:01
Negative	HD4	07:01				01:03		03:03		02:01		02:01		01:03	
	HC196	04:04				01:03		03:02	04:02	03:01	06:01	03:01	03:03	01:03	

(Table S2 continued on next page)

TCR 1F6$_{C61}$															
	B-LCL	DRB1		DRB3		DRB4	DRB5	DQB1		DPB1		DQA1		DPA1	
Specific	Donor 1	01:01	13:01	02:02				05:01	06:03	02:01	04:01	01	01	01:03	
	JY	04:04	13:01	01:01		01:03		03:02	06:03	02:01	04:01	01:03	03:01	01:03	
	TUBO	01:01	13:01	02:02				05:01	06:03	02:01	04:01	01:01	01:03	01:03	01:03
	HD9	01:01	11:04	02:02				03:01	05:01	04:01	04:02	01:01	05:05	01:03	
	HC120	13:01	13:02	01:01	03:01			06:03	06:04	03:01		01:02	01:03	01:03	
	HC242	15:01					01:01	06:02	06:03	04:01		01:02		01:03	02:02
	HC243	13:01	13:02	01:01	03:01			06:03	06:04	03:01	11:01	01:02	01:03	01:03	02:01
	HC306	11:01	13:01	02:02				03:01	06:03	04:01	13:01	01:03	05:05	01:03	02:01
Negative	Donor 6	04:03	11:04	02:02		01:03		03:01	03:02	04:01	15:01	03:01	05:05	01:03	
	OZB	11:04		02:02				03:01		04:02	10:01	05:05		01:03	02:01
	HD5	11:04	16:01	02:02			02:02	03:01	05:02	04:01	10:01	01:02	05:05	01:03	02:01
	HD6	04:02	11:01	02:02		01:03		03:01	03:02	02:01		03:01	05:05	01:03	
	HD7	03:01	04:01	01:01		01:03		02:01	03:01	01:01	16:01	03:03	05:01	01:03	02:01
	HD8	03:01	04:03	02:02		01:03		02:01	03:05	04:01	26:01	03:01	05:01	01:03	02:01
	HC196	04:04				01:03		03:02	04:02	03:01	06:01	03:01	03:03	01:03	
	HC244	03:01	07:01	01:01		01:03		02:01	02:02	04:01	10:01	02:01	05:01	01:03	02:01
	HC245	13:02	15:01	03:01			01:01	06:02	06:04	02:01	04:01	01:02		01:03	
	HC247	15:01					01:01	06:02		02:01	04:02	01:02		01:03	
	HC299	04:03	09:01			01:03		03:02	03:03	05:01	13:01	03:01	03:02	02:06	04:01
	HC303	04:04	13:03	01:01		01:03		03:01	03:02	04:01		03:01	05:05	01:03	01:03
	HC318	04:01	11:02	02:02		01:03		03:01		04:01	15:01	03:03	05:05	01:03	01:03
	HC319	03:01	11:04	02:02				02:01	03:01	04:01	15:01	05:01	05:05	01:03	01:04

TCR 3G3$_{preS9}$															
	B-LCL	DRB1		DRB3		DRB4	DRB5	DQB1		DPB1		DQA1		DPA1	
Specific	Donor 1	01:01	13:01	02:02				05:01	06:03	02:01	04:01	01	01	01:03	
	JY	04:04	13:01	01:01		01:03		03:02	06:03	02:01	04:01	01:03	03:01	01:03	
	TUBO	01:01	13:01	02:02				05:01	06:03	02:01	04:01	01:01	01:03	01:03	01:03
	HD9	01:01	11:04	02:02				03:01	05:01	04:01	04:02	01:01	05:05	01:03	
	HC120	13:01	13:02	01:01	03:01			06:03	06:04	03:01		01:02	01:03	01:03	
	HC242	15:01					01:01	06:02	06:03	04:01		01:02		01:03	02:02
	HC243	13:01	13:02	01:01	03:01			06:03	06:04	03:01	11:01	01:02	01:03	01:03	02:01
	HC306	11:01	13:01	02:02				03:01	06:03	04:01	13:01	01:03	05:05	01:03	02:01
Unspecific	Donor 6	04:03	11:04	02:02		01:03		03:01	03:02	04:01	15:01	03:01	05:05	01:03	
	OZB	11:04		02:02				03:01		04:02	10:01	05:05		01:03	02:01
	HD5	11:04	16:01	02:02			02:02	03:01	05:02	04:01	10:01	01:02	05:05	01:03	02:01
	HD6	04:02	11:01	02:02		01:03		03:01	03:02	02:01		03:01	05:05	01:03	
	HD7	03:01	04:01	01:01		01:03		02:01	03:01	01:01	16:01	03:03	05:01	01:03	02:01
	HC244	03:01	07:01	01:01		01:03		02:01	02:02	04:01	10:01	02:01	05:01	01:03	02:01
	HC318	04:01	11:02	02:02		01:03		03:01		04:01	15:01	03:03	05:05	01:03	01:03
	HC319	03:01	11:04	02:02				02:01	03:01	04:01	15:01	05:01	05:05	01:03	01:04
Negative	HD8	03:01	04:03	02:02		01:03		02:01	03:05	04:01	26:01	03:01	05:01	01:03	02:01
	HC196	04:04				01:03		03:02	04:02	03:01	06:01	03:01	03:03	01:03	
	HC245	13:02	15:01	03:01			01:01	06:02	06:04	02:01	04:01	01:02		01:03	
	HC247	15:01					01:01	06:02		02:01	04:02	01:02		01:03	
	HC299	04:03	09:01			01:03		03:02	03:03	05:01	13:01	03:01	03:02	02:06	04:01
	HC303	04:04	13:03	01:01		01:03		03:01	03:02	04:01		03:01	05:05	01:03	01:03

TCR S123$_{S21}$															
	B-LCL	DRB1		DRB3		DRB4	DRB5	DQB1		DPB1		DQA1		DPA1	
Specific	Donor 6	04:03	11:04	02:02		01:03		03:01	03:02	04:01	15:01	03:01	05:05	01:03	01:04
	HC318	04:01	11:02	02:02		01:03		03:01		04:01	15:01	03:03	05:05	01:03	01:03
	HC319	03:01	11:04	02:02				02:01	03:01	04:01	15:01	05:01	05:05	01:03	01:04
Negative	Donor 1	01:01	13:01	02:02				05:01	06:03	02:01	04:01	01	01	01:03	
	JY	04:04	13:01	01:01		01:03		03:02	06:03	02:01	04:01	01:03	03:01	01:03	
	OZB	11:04		02:02				03:01		04:02	10:01	05:05		01:03	02:01
	TUBO	01:01	13:01	02:02				05:01	06:03	02:01	04:01	01:01	01:03	01:03	01:03
	HD5	11:04	16:01	02:02			02:02	03:01	05:02	04:01	10:01	01:02	05:05	01:03	02:01
	HD6	04:02	11:01	02:02		01:03		03:01	03:02	02:01		03:01	05:05	01:03	
	HD7	03:01	04:01	01:01		01:03		02:01	03:01	01:01	16:01	03:03	05:01	01:03	02:01
	HD8	03:01	04:03	02:02		01:03		02:01	03:05	04:01	26:01	03:01	05:01	01:03	02:01
	HD9	01:01	11:04	02:02				03:01	05:01	04:01	04:02	01:01	05:05	01:03	
	HC120	13:01	13:02	01:01	03:01			06:03	06:04	03:01		01:02	01:03	01:03	
	HC196	04:04				01:03		03:02	04:02	03:01	06:01	03:01	03:03	01:03	
	HC242	15:01					01:01	06:02	06:03	04:01		01:02		01:03	02:02
	HC243	13:01	13:02	01:01	03:01			06:03	06:04	03:01	11:01	01:02	01:03	01:03	02:01
	HC244	03:01	07:01	01:01		01:03		02:01	02:02	04:01	10:01	02:01	05:01	01:03	02:01
	HC245	13:02	15:01	03:01			01:01	06:02	06:04	02:01	04:01	01:02		01:03	
	HC247	15:01					01:01	06:02		02:01	04:02	01:02		01:03	
	HC299	04:03	09:01			01:03		03:02	03:03	05:01	13:01	03:01	03:02	02:06	04:01
	HC303	04:04	13:03	01:01		01:03		03:01	03:02	04:01		03:01	05:05	01:03	01:03
	HC306	11:01	13:01	02:02				03:01	06:03	04:01	13:01	01:03	05:05	01:03	02:01

(Table S2 continued)

Table S3: Characteristics of the specific peptide:MHC complex identified for each TCR. Left side: Overview of MHC restrictions for each TCR as identified in Figure 3, Figure S2 and Table S2. Right side: Binding affinities of HBV peptides to their restricting MHC allele predicted via the IEDB MHC-II binding prediction tool (http://tools.iedb.org/mhcii/), with IC_{50} values from recommended algorithms NetMHCIIpan or NN-align. In addition, binding affinities were measured by an MHC-ligand binding assay.[12]

TCR	Peptide	Restricting MHC molecule		Binding affinity pMHC IC_{50} (nM)	
		α-chain	β-chain	Predicted	Measured
1C11	C61	DRA	DRB3*02:02	3700.0	4880.0
		DRA	DRB1*01:01	32.4	7.8
2F7					
3A6					
P74					
1F6		DQA1*01:01	DQB1*06:03	232.1	382.0
3H6	C91	DRA	DRB1*13:01	193.4	3222.0
1G11					
2F2					
1D12	C113	DRA	DRB1*01:01	12.1	27.8
		DQA1*01:01	DQB1*05:01	274.2	17.9
CP11		DRA	DRB3*02:02	1566.0	3030.0
3G3	preS9	DQA1*01:01	DQB1*06:03	391.0	3240.0
1F1	S17	DPA1*01:03	DPB1*02:01	2.2	1.0
1A2		DRA	DRB1*07:01	44.3	1.3
1C1					
S123	S21	DPA1*01:03	DPB1*15:01	71.2	n.a.
1E1	S36		DRB1*01:01	5.7	3.1
1B9					
2E7					
2H12					
1D4	P774	DPA1*01:03	DPB1*04:01	382.2	0.9

Table S4: Summary table of TCR characterization. Transduction (transd.) rates in % of $CD4^+$ T cells and vector copy number (VCN) as an average number of integrates per cell (avg./cell) are indicated for a representative cell batch. MFI of TCR^+ populations in flow cytometry from four independent transductions is normalized (norm.) to mean of each experiment. The recognition of processed (proc.) antigen is scored according to TNF-α secretion in co-culture with B-LCLs: low (<100 pg/ml), medium (100-200 pg/ml) and high (>200 pg/ml). The number of recognized HBV genotypes (Gt) by each TCR is given as a number of four (x/4) genotypes tested: A/B/C/D. Functional avidity is specified as EC_{50} in nM calculated from proliferation assays with peptide titration. $CD4^+IL\text{-}2^+$ cells and $CD8^+GrzB^+$ in % of TCR^+ T cells are listed representative for cytokine secretion. Cytotoxicity endpoint values for $CD4^+$ and $CD8^+$ T cells after 24 hours of co-culture are indicated in % relative (rel.) to the unloaded control. n.d.= none determined; EC_{50} could not be calculated when a plateau of maximum response was not reached and cytotoxicity could not be determined when matching single MHCII-transfectant adherent target cell lines were not available.

TCR	Peptide	MHC restriction β-chain	Transd. rate (%)	VCN (avg./ cell)	MFI (norm.)	Proc. antigen (score)	HBV Gt (x/4)	EC_{50} (nM)	Cytokine secretion (% of TCR^+T cells)		Cytotoxicity 24 h (% rel. to control)	
									CD4 (IL-2)	CD8 (GrzB)	CD4	CD8
1C11	C61	DRB1*01:01	87.6	4.7	0.70	low	4	82	89.3	92.6	2.4	2.8
2F7			67.3	2.0	1.24	low	4	83	87.1	82.7	4.6	2.5
3A6			70.6	1.9	0.89	low	4	94	87.0	83.5	4.0	2.7
P74			84.5	3.1	0.65	low	4	47	81.0	69.9	4.7	3.2
1F6		DQB1*06:03	76.7	1.8	1.57	low	1	92	78.5	73.2	n.d.	
3H6	C91	DRB1*13:01	73.2	2.1	1.09	high	3	n.d.	61.9	46.5	30.5	3.7
1G11			59.3	1.2	1.28	high	3	n.d.	69.2	47.7	34.3	8.0
2F2			84.6	2.8	0.89	low	4	n.d.	25.8	11.3	n.d.	
1D12	C113	DRB1*01:01	59.2	3.9	0.26	high	4	n.d.	70.6	70.0	10.9	5.3
CP11		DRB3*02:02	75.1	2.6	0.78	high	4	n.d.	76.4	72.3	n.d.	
3G3	preS9	DQB1*06:03	86.0	2.6	1.61	n.d.	3	42	76.4	82.3	n.d.	
1F1	S17	DPB1*02:01	78.0	2.6	1.06	low	3	3.3	88.8	86.1	39.3	4.9
1A2		DRB1*07:01	75.9	4.4	0.48	low	3	8	89.8	86.0	21.1	22.5
1C1			71.5	2.7	0.62	high	3	4.7	88.3	75.2	24.1	16.2
S123	S21	DPB1*15:01	75.7	2.6	0.71	low	4	9.9	66.8	65.7	n.d.	
1E1	S36	DRB1*01:01	65.1	2.5	0.32	high	1	3.4	92.3	90.3	2.7	2.1
1B9			75.6	2.5	0.59	high	1	8.5	89.3	84.9	3.3	2.0
2E7			72.4	1.7	1.30	med	1	3.2	86.8	75.7	2.9	1.8
2H12			85.5	2.4	2.56	med	1	8.8	81.2	73.1	3.1	2.5
1D4	P774	DPB1*04:01	82.7	2.8	1.20	n.d.	4	1.6	85.2	78.5	n.d.	

Supplemental methods

Stimulation of PBMC

PBMC were stimulated as described in the main manuscript. For stimulation of PBMC of donors 1 and 2, which resulted in identification of 17 out of the 20 TCRs, single peptides from Table S1 were used. The C61-specific T cell clone from donor 3 was obtained from the group of N. Gruener[10] and restimulated once with 1 µM of peptide C61, which resulted in identification of TCR $P74_{C61}$. For stimulation of donor 4, a pool of HBV core peptides was used at a final concentration of 2.7 µg/ml of each peptide (MDIDPYKEFGATVEL, LSFLPSDFFPSVRDL, FLPSDFFPSVRDLLD, RDLLDTASALYREAL, PHHTALRQAILCWGE, GRETVLEYLVSFGVW, EYLVSFGVWIR-TPPA, VSFGVWIRTPPAYRP, TVVRRRDRGRSPRRR), which resulted in identification of TCR $CP11_{C113}$. For stimulation of donor 5, a pool of HBV S peptides was used at a final concentration of 11 µg/ml of each peptide (LVLQAGFFLLTRILT, AGFFLLTRILTIPKS, LLTRILTIPKSLDSW, FFLLTRILTIPQSLD), which resulted in identification of TCR $S123_{S17}$.

Identification of HBV-specific T cells clones

96-well plates were microscopically screened for growing T cell clones. Donor-derived B-LCLs were irradiated (50 Gy), loaded with 1 µM of the respective peptide for 2 hours at 37 C°C and then washed twice with PBS. 20 µl/well of each visually outgrown T cell clone was co-cultured with $5x10^4$ peptide-pulsed or unloaded B-LCLs at 37 °C. TNF-α secretion via ELISA (BD) was measured after 16 hours from supernatants to determine HBV specificity.

Analysis of TCR repertoire

For RNA extraction from T cell clones, Trizol (Thermo Fisher Scientific) was used according to the manufacturer's instructions including 1-bromo-3-chloropropane (Sigma-Aldrich) and 20 µl Linear Acrylamide (Thermo Fisher Scientific). RNA was reverse transcribed to cccDNA using Superscript II (Thermo Fisher Scientific). TCR chains were amplified from cDNA with Illustra PureTaq PCR Beads (GE) using degenerated primers, VPANHUM (5'-TGAGTGTCCCPGAPGG2P-3') and CA2 (5'-GTGACACATTTGTTTGAGAATC-3') for α-chains, VP1 (5'-GCIITKTIYTGGTAYMGACA-3') or VP2 (5'-CTITKTWTTGGTAYCIKCAG-3') and CP1 (5'-GCACCTCCTTCCCATTCAC-3') for β-chains. The sequencing results were blasted with IMGT/V-QUEST to identify TCR chains. When degenerated primer PCR did not give a conclusive result, PCRs were repeated with primers specific for the individual α- or β-variable chain as described elsewhere.[13]

Cloning of TCR chains

5' Primers including a Kozak sequence and a NotI restriction site were designed according to the variable region identified for each TCR. An EcoRI or BsrGI restriction site was added to the 3' primers for the constant regions: TRAC (TRAC-EcoRI 5'-GGAATTCTCAGCTGGACCACAGCCGCAGC-3'and TRAC1-BsrGI 5'-CTTGTACATCAGCTGGACCACAGCCGCAGC-3') or TRBC (TRBC1-EcoRI 5'-TGGAATTCTCAGAAATCCTTTCTCTTGACC-3' and TRBC2-EcoRI 5'-TGGAATTCCTAGCCTCTGGAATCCTTTCTC-3'). TCR chains were amplified from cDNA with Phusion Hot Start II (New England Biolabs) and cloned separately into the retroviral vector MP71.[14] Variable domains of TCRs with confirmed HBV-specificity were codon-optimized and synthesized at GeneArt (Regensburg), fused by a P2A element and substituted with murine constant domains as described previously.[15]

Generation of stable producer cells

Stable 293GP-R30 (RD114-pseudotype) producer cells were generated by transduction with cell culture supernatant from 293GP-GLV9 cells[16] that had been transfected with TCR plasmids as described earlier.[17] Producer cell lines were transiently transfected with the murine CD3 δγεζ chains cloned into the vector pcDNA3.1 from the vector pMIG II Murine CD3 WTdelta-F2A-gamma-T2A-epsilon-P2A-zeta (Addgene) using the Lipofectamine 2000 transfection reagent (Thermo Fisher Scientific) prior to enrichment of TCR^+ cells with a FACS Aria II (BD) or a MoFlo II cell sorter (Beckmann Coulter).

Prediction of peptide to MHC binding affinity

Binding affinities of HBV peptides to the respective MHC allele were predicted using the NetMHCIIpan 3.2 algorithm based on 18-mers[1] or the IEDB MHC-II binding prediction tool (http://tools.iedb.org/mhcii/) using the recommended algorithms based on 15-mers.

MHC-ligand binding assay

Binding affinities of HBV peptides to their restricting MHC allele were measured based on their ability to inhibit the binding of a radiolabeled probe peptide to the purified MHC molecule as described previously.[12]

Supplemental references

1. Jensen, K.K., Andreatta, M., Marcatili, P., Buus, S., Greenbaum, J.A., Yan, Z., Sette, A., Peters, B., Nielsen, M. (2018). Improved methods for predicting peptide binding affinity to MHC class II molecules. Immunology 154, 394-406.

2. Barnaba, V., Franco, A., Alberti, A., Balsano, C., Benvenuto, R., Balsano, F. (1989). Recognition of hepatitis B virus envelope proteins by liver-infiltrating T lymphocytes in chronic HBV infection. J Immunol 143, 2650-2655.

3. Barnaba, V., Franco, A., Paroli, M., Benvenuto, R., De Petrillo, G., Burgio, V.L., Santilio, I., Balsano, C., Bonavita, M.S., Cappelli, G., et al. (1994). Selective expansion of cytotoxic T lymphocytes with a CD4+CD56+ surface phenotype and a T helper type 1 profile of cytokine secretion in the liver of patients chronically infected with Hepatitis B virus. J Immunol 152, 3074-3087.

4. Chisari, F.V., Ferrari, C. (1995). Hepatitis B virus immunopathogenesis. Annu Rev Immunol 13, 29-60.

5. Desombere, I., Gijbels, Y., Verwulgen, A., Leroux-Roels, G. (2000). Characterization of the T cell recognition of hepatitis B surface antigen (HBsAg) by good and poor responders to hepatitis B vaccines. Clin Exp Immunol 122, 390-399.

6. Ferrari, C., Bertoletti, A., Penna, A., Cavalli, A., Valli, A., Missale, G., Pilli, M., Fowler, P., Giuberti, T., Chisari, F.V., et al. (1991). Identification of immunodominant T cell epitopes of the hepatitis B virus nucleocapsid antigen. J Clin Invest 88, 214-222.

7. Honorati, M.C., Dolzani, P., Mariani, E., Piacentini, A., Lisignoli, G., Ferrari, C., Facchini, A. (1997). Epitope specificity of Th0/Th2 CD4+ T-lymphocyte clones induced by vaccination with rHBsAg vaccine. Gastroenterology 112, 2017-2027.

8. Mizukoshi, E., Sidney, J., Livingston, B., Ghany, M., Hoofnagle, J.H., Sette, A., Rehermann, B. (2004). Cellular immune responses to the hepatitis B virus polymerase. J Immunol 173, 5863-5871.

9. Pajot, A., Michel, M.L., Mancini-Bourgine, M., Ungeheuer, M.N., Ojcius, D.M., Deng, Q., Lemonnier, F.A., Lone, Y.C. (2006). Identification of novel HLA-DR1-restricted epitopes from the hepatitis B virus envelope protein in mice expressing HLA-DR1 and vaccinated human subjects. Microbes Infect 8, 2783-2790.

10. Raziorrouh, B., Heeg, M., Kurktschiev, P., Schraut, W., Zachoval, R., Wendtner, C., Wächtler, M., Spannagl, M., Denk, G., Ulsenheimer, A., et al. (2014). Inhibitory phenotype of HBV-specific CD4+ T-cells is characterized by high PD-1 expression but absent coregulation of multiple inhibitory molecules. PLoS One 9, e105703.

11. Ru, Z., Xiao, W., Pajot, A., Kou, Z., Sun, S., Maillere, B., Zhao, G., Ojcius, D.M., Lone, Y.C., Zhou, Y. (2012). Development of a humanized HLA-A2.1/DP4 transgenic mouse model and the use of this model to map HLA-DP4-restricted epitopes of HBV envelope protein. PLoS One 7, e32247.

12. Sidney, J., Southwood, S., Moore, C., Oseroff, C., Pinilla, C., Grey, H.M., Sette, A. (2013). Measurement of MHC/peptide interactions by gel filtration or monoclonal antibody capture. Curr Protoc Immunol Chapter 18, Unit 18.13.

13. Steinle, A., Reinhardt, C., Jantzer, P., Schendel, D.J. (1995). In vivo expansion of HLA-B35 alloreactive T cells sharing homologous T cell receptors: evidence for maintenance of an oligoclonally dominated allospecificity by persistent stimulation with an autologous MHC/peptide complex. J Exp Med 181, 503-513.

14. Engels, B., Cam, H., Schüler, T., Indraccolo, S., Gladow, M., Baum, C., Blankenstein, T., Uckert, W. (2003). Retroviral vectors for high-level transgene expression in T lymphocytes. Hum Gene Ther 14, 1155-1168.

15. Sommermeyer, D., Uckert, W. (2010). Minimal amino acid exchange in human TCR constant regions fosters improved function of TCR gene-modified T cells. J Immunol 184, 6223-6231.

16. Ghani, K., Wang, X., de Campos-Lima, P.O., Olszewska, M., Kamen, A., Riviere, I., Caruso, M. (2009). Efficient human hematopoietic cell transduction using RD114- and GALV-pseudotyped retroviral vectors produced in suspension and serum-free media. Hum Gene Ther 20, 966-974.

17. Wisskirchen, K., Metzger, K., Schreiber, S., Asen, T., Weigand, L., Dargel, C., Witter, K., Kieback, E., Sprinzl, M.F., Uckert, W., et al. (2017). Isolation and functional characterization of hepatitis B virus-specific T-cell receptors as new tools for experimental and clinical use. PLoS One 12, e0182936.
