# Supplementary material for: Characterization of a library of 20 HBV-specific MHC class II-restricted T cell receptors
Source: Mol Ther Methods Clin Dev. 2021 Oct 29;23:476–89. doi: 10.1016/j.omtm.2021.10.012 (PMC8605085; doi:10.1016/j.omtm.2021.10.012)
Supplement: Document S2. Article plus supplemental information [file mmc2.pdf]

Molecular Therapy
Methods & Clinical Development
Original Article

Characterization of a library of 20 HBV-specific MHC class II-restricted T cell receptors

Sophia Schreiber,[1,2] Melanie Honz,[2] Weeda Mamozai,[2] Peter Kurktschiev,[3] Matthias Schiemann,[4,5] Klaus Witter,[6] Eugene Moore,[7] Christina Zielinski,[2,5] Alessandro Sette,[7,8] Ulrike Protzer,[1,2,5] and Karin Wisskirchen[1,2,5]

[1]Institute of Virology, Helmholtz Zentrum München, 81675 Munich, Germany; [2]Institute of Virology, School of Medicine, Technical University of Munich, 81675 Munich, Germany; [3]Medical Department II and Institute for Immunology, Hospital of the Ludwig-Maximilians-University (LMU) Munich, 81377 Munich, Germany; [4]Institute for Medical Microbiology, Immunology and Hygiene, School of Medicine, Technical University of Munich, 81675 Munich, Germany; [5]German Center for Infection Research (DZIF), Munich Partner Site, Munich, Germany; [6]Laboratory for Immunogenetics and Molecular Diagnostics, Hospital of the Ludwig-Maximilians-University (LMU) Munich, 81377 Munich, Germany; [7]Center for Infectious Disease and Vaccine Research, La Jolla Institute for Immunology (LJI), La Jolla, CA 92037, USA; [8]Department of Medicine, Division of Infectious Diseases and Global Public Health, University of California, San Diego (UCSD), La Jolla, CA 92037, USA

$CD4^+$ T cells play an important role in the immune response against cancer and infectious diseases. However, mechanistic details of their helper function in hepatitis B virus (HBV) infection in particular, or their advantage for adoptive T cell therapy remain poorly understood as experimental and therapeutic tools are missing. Therefore, we identified, cloned, and characterized a comprehensive library of 20 MHC class II-restricted HBV-specific T cell receptors (TCRs) from donors with acute or resolved HBV infection. The TCRs were restricted by nine different MHC II molecules and specific for eight different epitopes derived from intracellularly processed HBV envelope, core, and polymerase proteins. Retroviral transduction resulted in a robust expression of all TCRs on primary T cells. A high functional avidity was measured for all TCRs specific for epitopes S17, S21, S36, and P774 (half-maximal effective concentration [EC_{50}] <10 nM), or C61 and preS9 (EC_{50} <100 nM). Eight TCRs recognized peptide variants of HBV genotypes A to D. Both $CD4^+$ and $CD8^+$ T cells transduced with the MHC II-restricted TCRs were polyfunctional, producing interferon (IFN)-γ, tumor necrosis factor (TNF)-α, interleukin (IL)-2, and granzyme B (GrzB), and killed peptide-loaded target cells. Our set of MHC class II-restricted TCRs represents an important tool for elucidating $CD4^+$ T cell help in viral infection with potential benefit for T cell therapy.

INTRODUCTION

Chronic hepatitis B (CHB) continuous to be a global health problem, with 296 million people affected worldwide.[1] Current treatment options, such as nucleos(t)ide analogues and pegylated interferon (IFN)-α, are rarely able to cure the infection, and chronically infected patients remain at an elevated risk of developing liver cirrhosis and eventually hepatocellular carcinoma (HCC) during their lifetimes. Chronic hepatitis B virus (HBV) infection is marked by a progressive functional exhaustion and ultimately depletion of virus-specific $CD4^+$ and $CD8^+$ T cells.[2] Naturally resolving, acute hepatitis B, on the other hand, is characterized by a strong and polyfunctional T cell response, which can be restored in CHB patients by transfer of HBV-specific T cells.[3] The therapeutic potential of transferring HBV immunity initially emerged through clinical reports of CHB patients who cleared the infection after receiving a bone marrow transplant from HBV-immune donors, giving rise to HBV-specific $CD4^+$ and $CD8^+$ T cells as well as B cells.[4,5] Hence, adoptive T cell therapy represents a promising therapeutic approach to treat CHB- and HBV-induced HCC.[6] In order to imitate such an effective immune response needed for clearing the infection, we have previously generated both HBV-specific chimeric antigen receptors (CARs)[7] and MHC class I (MHC I)-restricted T cell receptors (TCRs)[8] that showed promising antiviral activity in models of CHB.[9,10] In a humanized mouse model, in which transplanted human hepatocytes were infected with HBV, MHC I-restricted HBV-specific T cells even led to undetectable serum levels of HBV surface antigen and HBV DNA,[10] a status that is described as functional cure.[11] However, when T cells vanished, a viral rebound could only be contained with an HBV entry inhibitor,[10] underlining the necessity for long-term persistence of anti-HBV immunity.

Although cytotoxic $CD8^+$ T cells are key to clearing HBV infection by destroying infected cells, $CD4^+$ T cells are known to play an important role.[12,13] They are indispensable for viral clearance as, for example, chimpanzees are unable to clear HBV infection when $CD4^+$ T cells are depleted early on during the course of infection.[14] Besides their direct role in targeting infected or malignant cells, $CD4^+$ T cells provide help to other immune cells. For instance, they license dendritic cells to cross-present viral antigen to $CD8^+$ T cells,[15] and $CD8^+$ T cells that have received $CD4^+$ T cell help during priming and second

Received 15 July 2021; accepted 27 October 2021;
https://doi.org/10.1016/j.omtm.2021.10.012.

Correspondence: Karin Wisskirchen, PhD, Institute of Virology, Trogerstrasse 30, 81675 Munich, Germany.
E-mail: karin.wisskirchen@tum.de

 © 2021 The Authors.
This is an open access article under the CC BY license (http://creativecommons.org/licenses/by/4.0/).

antigen encounter differentiate to memory cells more effectively, proliferate better, and increase their migratory and invasive potential.[16] Moreover, CD4$^+$ T cells can induce maturation and antibody production in B cells of the same antigen specificity. This interaction requires the engagement of an MHC class II (MHC II)-restricted TCR specific for an antigen-derived peptide with the peptide:MHC complex (pMHC) on a B cell that has taken up the same antigen via its B cell receptor.[17] Little is known about the detailed functionality of HBV-specific CD4$^+$ T cells as the few available studies have focused on describing their *ex vivo* immunophenotypes. Presumably they exert an indirect role in viral clearance by helping CD8$^+$ T cells and B cells, which is especially important in fighting a poorly immunogenic virus like HBV.[18] Recently, a higher frequency of HBV-specific CD4$^+$ T cells was positively correlated with hepatitis B e antigen (HBeAg) or hepatitis B surface antigen (HBsAg) loss after flares in CHB patients.[19] Hence, overcoming the low numbers and dysfunctional phenotype of HBV-specific CD4$^+$ T cells in CHB[20] might also promote viral clearance.

Along that line, the importance of CD4$^+$ T cells in fighting viral infections and tumor diseases also implies their benefit for immunotherapy and potentially for adoptive T cell therapy,[21,22] including treatment of CHB- and HBV-induced HCC. To date, most T cell therapeutic approaches have focused on tumor diseases using genetically engineered T cells expressing an MHC I-restricted TCR or a CAR, and those T cell products typically also include redirected CD4$^+$ T cells. Co-transfer of these CD4$^+$ T cells can confer superior therapeutic efficacy to some extent[23,24] but does not provide the potential benefits of CD4$^+$ T cells naturally engaging with peptides presented on MHC II.[21] In mice, they were shown to alter the tumor microenvironment through interaction with antigen-presenting cells (APCs)[25] and were required for the recruitment and cytolytic function of CD8$^+$ T cells.[26] In addition, IFN-γ-dependent interaction of CD4$^+$ T cells with non-hematopoietic cells was shown to interfere with tumor angiogenesis.[27] Moreover, clinical evidence is encouraging as a metastatic patient who received autologous NY-ESO-1-specific CD4$^+$ T cell clones reportedly went into complete remission after developing an endogenous multi-specific T cell response.[28] Although the application of MHC II-restricted T cells in T cell therapy has recently gained more attention,[21,22] only a few TCRs have been isolated to date, including one TCR against human papilloma virus (HPV) E7 as a viral target.[29] Indeed, the addition of redirected MHC II-restricted CD4$^+$ to MHC I-restricted CD8$^+$ T cells was shown to strongly increase tumor regression in a xenograft mouse model.[30] These results emphasize the potential that can be attributed to the use of CD4$^+$ T cells in adoptive T cell therapy, also in the context of viral infection.

In order to address the lack of knowledge on HBV-specific CD4$^+$ T cells and to improve the success rate of adoptive T cell transfer, experimental and therapeutic tools are needed. In the present study, we isolated MHC II-restricted TCRs from HBV-specific CD4$^+$ T cells. These TCRs target different epitopes of the HBV core, envelope, and polymerase proteins and were extensively characterized regarding MHC restriction, binding affinity, and recognition of antigen, so as to evaluate them and compare their overall applicability in adoptive T cell therapy of HBV infection.

RESULTS

TCRs isolated from HBV-specific CD4$^+$ T cells are expressed at high levels after retroviral transduction

First, we sought to isolate HBV-specific CD4$^+$ T cells and to identify their MHC II-restricted TCR sequences. Peripheral blood mononuclear cells (PBMCs) of donors with acute or resolved HBV infection (Figure 1A) were stimulated with peptides from HBV core (C), envelope (preS/S), and polymerase (P) proteins (Figure 1B), based on a literature review of known CD4$^+$ T cell epitopes, as well as *in silico* prediction for human leukocyte antigen (HLA)-DR1 and HLA-DR13 (Table S1). After two weeks, tumor necrosis factor (TNF)-α, and/or IFN-γ-secreting CD4$^+$ T cells were isolated by flow cytometry cell sorting and clonally expanded through limiting dilution cloning. Specificities comprised three core-derived peptides (C61, C91, C113), four envelope-derived peptides (preS9, S17, S21, and S36), and one polymerase-derived peptide (P774). Six peptides or overlapping parts of them had been described previously, and two peptides, preS9 and C91, were newly identified to be immunogenic (Table S1). In total, 20 TCRs with functional pairs of α and β chains were identified (Figure 1C). Next, to be able to express and characterize our MHC II-restricted HBV-specific TCRs in T cells, the respective TCR sequences were cloned into a retroviral vector, using codon-optimized variable α and β chain domains combined with murine constant domains (Figure 1B). Having established stable producer cell lines and a robust transduction protocol that yielded high but non-toxic transduction rates (Figure S1), we set out to characterize our panel of HBV-specific, MHC II-restricted TCRs in depth. First, we addressed the TCR expression level, which can be described with regard to the number of transduced cells in relation to the number of integrates as determined by quantitative polymerase chain reaction (qPCR), or the mean fluorescence intensity (MFI) in flow cytometry. With transduction rates ranging from approximately 60% to 90%, our cell batches featured an average of fewer than five integrated vector copies per cell (Figure 1D). Regarding the MFI of the TCR$^+$ population in flow cytometry, we found distinct levels for each TCR across four independent transductions, with, e.g., TCR $2H12_{S36}$ displaying a relatively high and $1D12_{C113}$ a consistently low MFI (Figure 1E). Overall, we were able to reproducibly generate HBV-specific T cells of good quality, i.e., high TCR expression despite low integrate number, which is an important safety aspect for the use of transduced T cells in adoptive T cell therapy.

TCRs recognize HBV peptides presented on nine different MHC II molecules

For TCRs to be used for research or clinical applications, it is a prerequisite to know the MHC molecule by which each TCR is restricted. The restriction of our TCRs from CD4$^+$ T cells was pre-defined by the MHC II alleles of the respective donors who were initially used for the isolation procedure (Figure 1A). The MHC restriction was primarily identified by co-culturing peptide-pulsed single MHC II transfectant target cells[31] (limited to availability) with TCR-transduced

A

Donor	Age	Sex	HBV infection status	DRB1*	DQB1*	DPB1*	DQA1*	DPA1*	DRB3*	DRB4*	DRB5*
1	52	M	resolved	01:01, 13:01	05:01, 06:03	02:01, 04:01	01, 01	01:03	02:02	n.d.	n.d.
2	33	F	resolved	01:01, 07:01	02:02, 05:01	03:01, 11:01	01, 02:01	01:03, 02:01	n.d.	01:01	n.d.
3	63	M	acute, resolving	01:01, 03:01	02:01, 05:01	03:01, 04:01	n.a.	n.a.	n.a.	n.a.	n.a.
4	24	M	acute	13:01, 14:54	05:03, 06:03	03:01	01, 01	01:03	02:02	n.d.	n.d.
5	57	M	acute, resolving	04:03, 11:04	03:01, 03:02	04:01, 15:01	03:01, 05:05	01:03, 01:04	02:02	01:03	n.d.

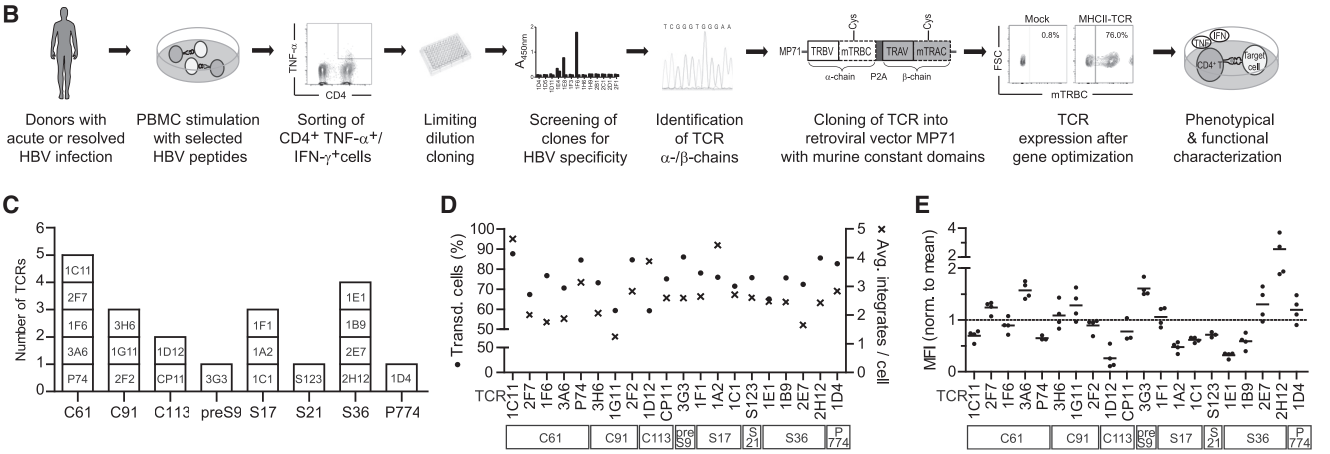

Figure 1. Identification and cloning of TCRs from HBV-specific CD4$^+$ T cell clones

(A) MHC II alleles of donors 1–5 with acute or resolved HBV infection. For donor 3, high-resolution MHC II typing was not available (n.a.) due to limited material resources. n.d., none detected. (B) Overview of the procedure: PBMCs from donors with acute or resolved HBV infection were stimulated with 1 μM of selected HBV peptides derived from core, envelope, or polymerase proteins. After two weeks, HBV-specific TNF-α or TNF-α/IFN-γ-secreting CD4$^+$ T cells were sorted by fluorescence-activated cell sorting and expanded from a single-cell level by limiting dilution cloning. After two further weeks of expansion with the addition of feeder cells and IL-2, clones were screened for HBV specificity and TCR α and β chains were identified by Sanger sequencing. Codon-optimized variable α and β domains (TRAV and TRBV) were cloned into the retroviral vector MP71 in combination with murine constant domains (mTRAC and mTRBC), including an additional cysteine (Cys) residue to increase pairing. TCR transduction rates were determined via flow cytometry by staining the mTRBC, here plotted against the cell volume (forward scatter, FSC). TCRs were phenotypically and functionally characterized through co-cultures of TCR-transduced T cells with peptide-pulsed target cells; i.e. HLA-matched B-LCLs or fibroblasts. (C) Final panel of identified TCRs. Clone and TCR names are indicated in square boxes. Peptide specificities from HBV core (C), envelope (preS/S), or polymerase (P) proteins are written below with the number indicating the peptide starting residue within the respective antigen. (D) Transduction rates (●, left y-axis) and average number of integrates per cell (x, right y-axis) of a representative cell batch. The average vector copy number per bulk cell population, i.e., including both transduced and non-transduced cells, was measured in a multiplex qPCR of the viral woodchuck hepatitis virus postregulatory element relative to the genomic single-copy gene *PTBP2*. (E) MFI of TCR$^+$ populations in flow cytometry from four independent transductions normalized to mean of each experiment. Square boxes below TCRs indicate peptide specificities.

T cells and measuring their TNF-α secretion (Figure 2). Based on this approach, the MHC II restriction was determined for most TCRs: all four S36-specific TCRs and C61-specific TCRs $1C11_{C61}$, $2F7_{C61}$, $3A6_{C61}$, and $P74_{C61}$ were HLA-DRB1*01:01-restricted. Interestingly, a promiscuous but specific binding behavior of TCR $1C11_{C61}$ toward DRB3*02:02 in the presence of the target peptide was noticed. All three C91-specific TCRs were DRB1*13:01-restricted. TCR $1D12_{C113}$ was both DRB1*01:01- and DQA1*01:01/DQB1*05:01-restricted, whereas $CP11_{C113}$ was DRB3*02:02-restricted. TCRs $1A2_{S17}$ and $1C1_{S17}$ were DRB1*07:01-restricted. In previous studies, peptides C61 and C113 had been associated with DRB1*01:01[20,32,33] and hence, we considered DRB1*01:01 to be the main restriction of TCRs $1C11_{C61}$ and $1D12_{C113}$.

Since the available single MHCII-transfectant target cells did not cover all donor MHC II molecules, the restriction of TCRs $1F6_{C61}$, $3G3_{preS9}$, $1F1_{S17}$, $S123_{S21}$, and $1D4_{P774}$ remained unclear. T cells transduced with these TCRs were therefore additionally co-cultured with a panel of partially HLA-matched B-LCLs (Figure S2, Table S2). A shared MHC allele between the respective donor and other cytokine-inducing B-LCLs suggested the specific MHC restriction. With this method, TCR $1F6_{C61}$ and TCR $3G3_{preS9}$ were found to be DQA1*01:01/DQB1*06:03-restricted; $3G3_{preS9}$, however, showed additional unspecific cytokine secretion (i.e., both in the presence and absence of peptide) in co-culture with a variety of B-LCLs that expressed the 01, 02, and 04 subtypes of the DRB1*11 allele family. To confirm this unspecific cross-reactivity, further experiments, e.g., with single MHC II transfectant target cells, would be needed. The MHC II restrictions for TCR $1F1_{S17}$, $S123_{S21}$, and $1D4_{P774}$ were identified to be DPA1*01:03/DPB1*02:01, DPA1*01:03/DPB1*15:01, and DPA1*01:03/DPB1*04:01, respectively.

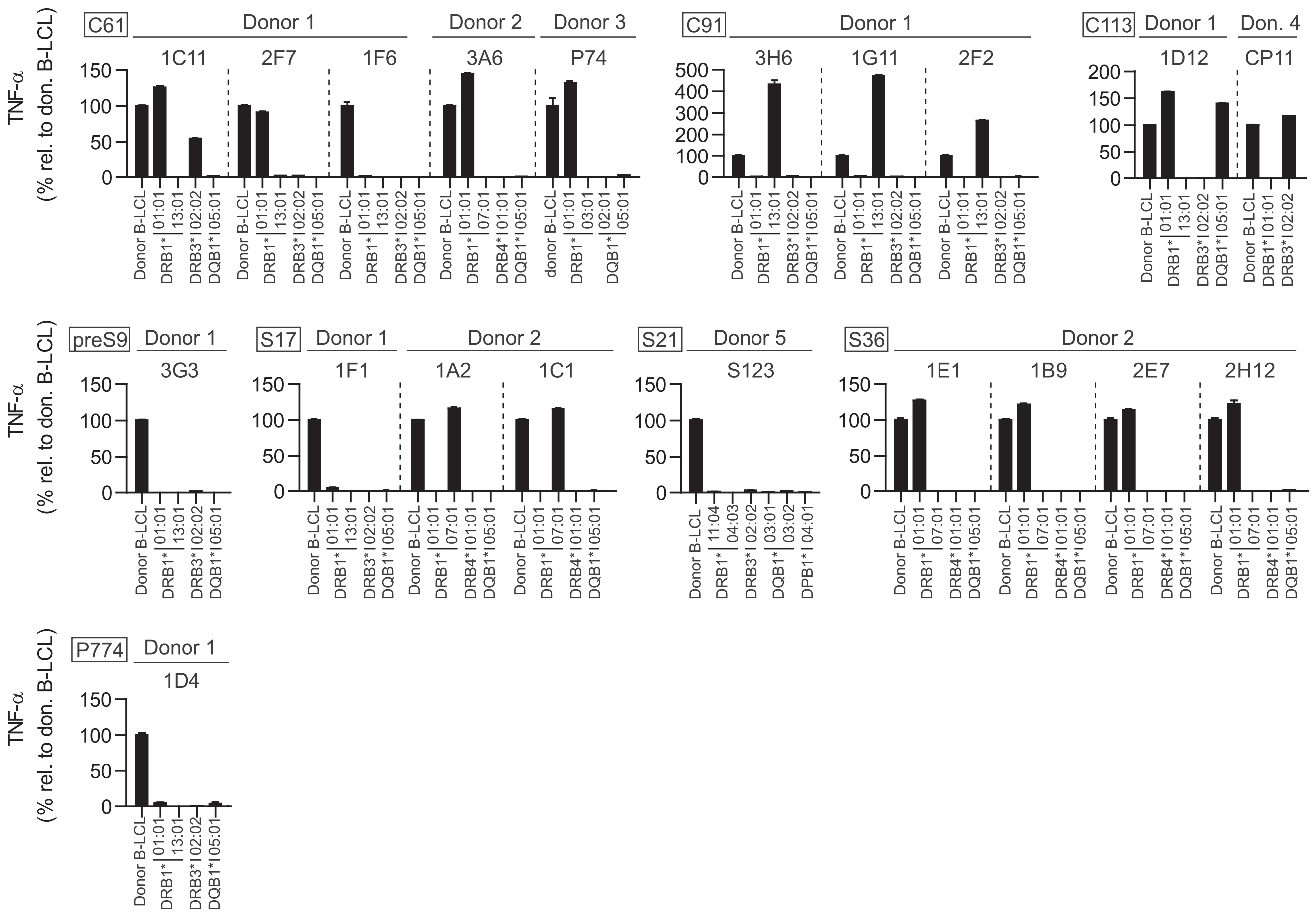

Figure 2. Verification of MHC II restrictions of HBV-specific TCRs

TCR-transduced $CD4^+$ T cells were co-cultured at an effector to target cell ratio of 2:1 with MHC II knockout (KO) fibroblasts or Raji-derived B-LCLs, stably transfected to express a single MHC II molecule and pulsed with 1 μM of target peptide. Each TCR was matched with target cells (limited to availability) co-expressing the corresponding MHC α and β chains of the respective donor; single MHC II transfectant cells are named after their respective MHC II β chain allele. TNF-α secretion was determined via ELISA and is shown relative to values from co-culture with the original B-LCLs of the respective donor. Data points represent mean values ±SD from triplicates. Controls without peptide were consistently below 5%, with the exception of TCR $2F2_{C91}$, which showed similarly high TNF-α secretion during co-culture with HLA-DRB1*13:01 target cells with or without peptide (data not shown). Square boxes at the top left of each graph indicate peptide specificities.

In total, nine MHC II restrictions were confirmed (Table S3), thereby covering a broad range of MHC haplotypes worldwide. TCRs with HLA-DR1 and -DP4 restriction are particularly interesting for research purposes of T cell therapy against HBV infection, as HLA-A2/DR1 and -A2/DP4 double-transgenic mouse models are available.[34,35]

MHC II-restricted TCR-transduced T cells recognize processed HBV antigen

Next, we asked whether the TCRs would recognize not only externally loaded peptide but also physiological epitopes, which requires antigen uptake, processing, and loading on MHC II. Accordingly, donor-derived B-LCLs were pre-incubated with native HBV core or small envelope protein, followed by co-culture with TCR-transduced T cells. Both antigens were taken up, processed intracellularly, and all corresponding HBV epitopes (C61, C91, C113, S17, S21, and S36) were presented to TCR-transduced T cells as indicated by their dose-dependent activation (Figure 3). TNF-α secretion in the absence of protein was below 5 pg/mL for all TCRs, with the exception of TCR $1B9_{S36}$, possibly related to a slight unspecific binding of TCR $1B9_{S36}$ or a minor background activation of T cells transduced with TCR $1B9_{S36}$. Of note, recognition of C61 was on average 10-fold lower than that of the other two core epitopes, C91 and C113, despite the fact that these two peptides had a lower binding affinity to the corresponding restricting MHC molecule (Table S3). This overall poorer response could argue for a diminished intracellular processing of the C61 peptide. Due to a lack of availability of the HBV large envelope and polymerase proteins, TCRs $3G3_{preS9}$ and $1D4_{P774}$ could not be included in this assay. Hence, one can only speculate that these TCRs similarly recognize processed antigen since they had been selected from a T cell repertoire primed by natural HBV infection.

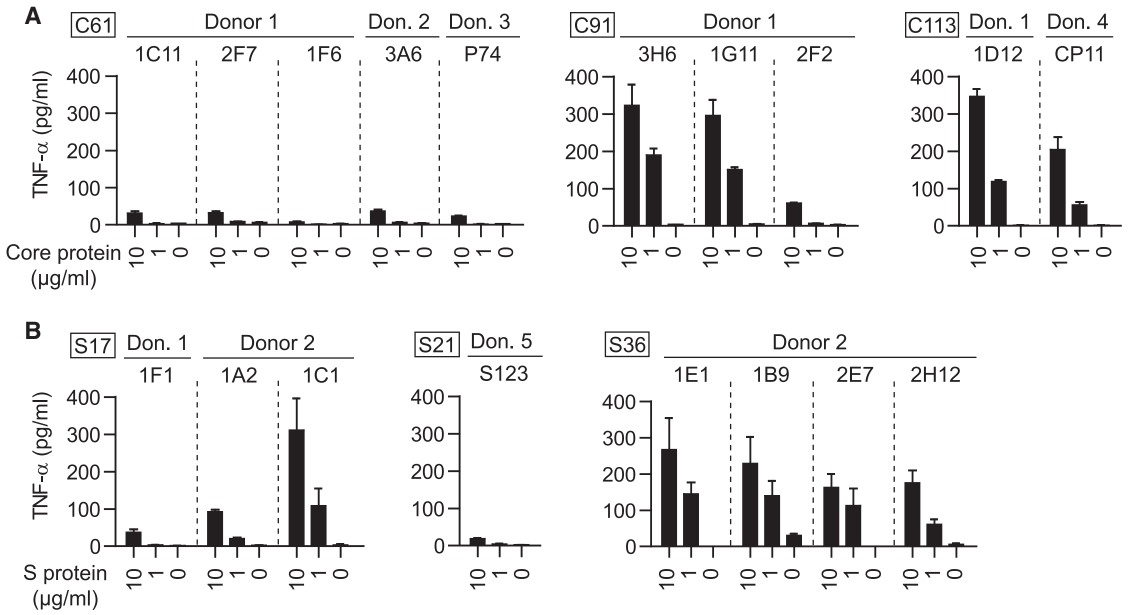

Figure 3. Recognition of physiologically processed HBV epitopes

TCR-transduced CD4+ T cells were co-cultured at an effector to target cell ratio of 2:1 with HLA-matched B-LCLs that had been pre-incubated for 4 h with 10 or 1 μg/mL of core protein (A) or small (S) envelope protein (B). TNF-α secretion was determined after 16 h of co-culture via ELISA. Data points represent mean values ±SD from triplicates. Square boxes at the top left of each graph indicate peptide specificities.

HBV-specific TCRs recognize peptides from different HBV genotypes

The interaction of the TCR with the pMHC complex is also influenced by variances in the peptide sequences. Therefore, we determined the recognition of different HBV genotypes by each TCR, which is considered favorable as it gives TCRs a broader range of therapeutic applicability. Out of the different HBV genotypes, genotype A-derived peptides had been used for initial T cell stimulation. The amino acid sequences of all eight epitopes are given in Table 1 for HBV genotypes A, B, C, and D, covering 79% of the worldwide HBV infections.[36] Across these four genotypes, HBV core and envelope epitopes C61, C91, C113, preS9, S17, S21, and S36 vary in their respective amino acid sequences, whereas the polymerase epitope P774 is fully conserved (Table 1).

To evaluate recognition of HBV genotype B, C, and D homologous peptides (Table 1), TCR-transduced T cells were co-cultured with HLA-matched B-LCLs pulsed with the respective peptides from (Figure 4). All HLA-DRB1*01:01-restricted C61-specific TCRs recognized all HBV genotypes, represented by the three different C61 variants. Interestingly, the DQA1*01:01/DQB1*06:03-restricted TCR $1F6_{C61}$ was only activated upon interaction with genotype A. C91-specific TCRs $3H6_{C91}$ and $1G11_{C91}$ detected both the A and B/C variant, whereas TCR $2F2_{C91}$ additionally bound to genotype D. The C113-specific TCRs recognized both epitope variants, thereby covering all four genotypes. The preS9-specific TCR $3G3_{preS9}$ was unable to recognize genotype D, which seems plausible given the major amino acid deletion in comparison with genotype A. TCR $S123_{S21}$ recognized all four genotypes, with A, C, and D being sequence identical and B differing by one amino acid exchange. Interestingly, this amino acid exchange seemed relevant for TCRs $1F1_{S17}$, $1A2_{S17}$, and $1C1_{S17}$, which were derived from other donors and only bound the A/C/D epitope variant. S36-specific TCRs interacted mostly with genotype A; however, $1B9_{S36}$ and $2H12_{S36}$ displayed minor binding toward genotypes B and C, respectively. In total, nine TCRs recognized several HBV genotypes and could thus be attributed a higher therapeutic range than TCRs that only recognize a single genotypic variant.

MHC II-restricted TCRs recognize nanomolar peptide concentrations

To further characterize the set of HBV-specific TCRs, we analyzed their sensitivity and functional avidity. This was indirectly measured via their potential to induce T cell proliferation and determining the peptide concentration that induced the half maximum (half-maximal effective concentration [EC_{50}]) proliferation capacity (Figure 5). All TCRs specific for epitopes S17, S21, S36, and P774, or C61 and preS9, showed EC_{50} values in a one-digit or two-digit nanomolar range, respectively. EC_{50} values of C91- and C113-specific TCRs could not be calculated and are expected to exceed the values of the TCRs above. In summary, most of the TCRs conveyed a high functional avidity with values typical for TCRs recognizing virus, i.e., foreign antigen.[37]

$CD4^+$ and $CD8^+$ T cells transduced with MHC II-restricted TCRs are polyfunctional

Next, we asked which functional profile MHC II-restricted TCRs would convey when transduced into either $CD4^+$ or $CD8^+$ T cells.

Table 1. Amino acid sequences of core, envelope, and polymerase peptides from HBV genotypes A, B, C, and D

	Genotype			
Peptide	A	B	C	D
C61	WGELMTLATWVGNNLEDP	WGELMNNLATWVGSNLEDP		WGELMTLATWVGGNLEDP
C91	TNMGLKIRQLLWFHISCL	VNMGLKIRQLLWFHISCL		TNMGLKFRQLLWFHISCL
C113	ETVLEYLVSFGVWIRTPP			ETVIEYLVSFGVWIRTPP
preS9	RKGMGTNLSVPNPLGFFP		RQGMGTNLSVPNPLGFFP	- - - MGQNLSTSNPLGFFP
S17	AGFFLLTRILTIPQSLDS	AGFFLLTKILTIPQSLDS	AGFFLLTRILTIPQSLDS	
S21	LLTRILTIPQSLDSW	LLTKILTIPQSLDSW	LLTRILTIPQSLDSW	
S36	WTSLNFLGGSPVCLGQNS	WTSLNFLGGTPVCLGQNS	WTSLNFLGGAPTCPGQNS	WTSLNFLGGTTVCLGQNS
P774	LRGTSFVYVPSALNPADD			

Genotype A peptides were used for isolation of TCRs, since donor 1 had been formerly diagnosed with HBV genotype A infection. Amino acid differences in comparison with genotype A are underlined. Peptide specificities from HBV core (C), envelope (preS/S), or polymerase (P) proteins are given with a number indicating the peptide starting residue within the respective antigen.

Therefore, the two T cell populations were purified after TCR engraftment and employed separately in co-cultures with peptide-loaded target cells. $CD4^+$ T cells (Figure 6A) generally secreted high amounts of TNF-α and IL-2, with most TCRs inducing TNF-α in >81% and IL-2 in >74% of $CD4^+$ TCR^+ T cells. C91-specific TCRs, especially TCR $2F2_{C91}$, showed a slightly reduced cytokine secretion (Figure 6A), which correlated with the lower functional avidity of this TCR observed before (Figure 5). IFN-γ secretion was relatively low and occurred only in around 15% of all $CD4^+$ TCR^+ T cells (Figure 6A). Interestingly, most TCRs also induced granzyme B (GrzB) secretion, a serine protease associated with cytotoxic activity, in up to 50% of $CD4^+$ TCR^+ cells.

In line with their designated function, $CD8^+$ T cells expressing MHC II-restricted TCRs had a predominantly cytotoxic profile as they secreted vast amounts of GrzB in 65%–90% of TCR^+ cells for most TCRs (Figure 6B). They also secreted IL-2 and TNF-α in up to 60% of $CD8^+$ TCR^+ T cells. The fraction of IFN-γ producing cells with around 20% was slightly higher in $CD8^+$ compared with $CD4^+$ T cells. Again, all C91-specific TCRs, especially TCR $2F2_{C91}$, induced slightly less cytokines and GrzB in $CD8^+$ T cells compared with other TCRs. In summary, expression in $CD8^+$ T cells revealed that MHC II-restricted TCRs did not require CD4 co-receptor binding. In addition, activation of transduced $CD4^+$ T cells induced a polyfunctional profile pointing at a T_H1 phenotype.

TCR-transduced $CD4^+$ and $CD8^+$ T cells are capable of killing MHC II-matched fibroblasts

In view of the strong GrzB secretion observed for most TCRs in both $CD8^+$ and $CD4^+$ T cells, the cytotoxic capacity of both T cell subsets was analyzed. To this end, peptide-loaded single MHC II transfectant fibroblasts served as target cells in a real-time cytotoxicity assay. In general, MHC II-restricted $CD4^+$ or $CD8^+$ T cells were able to recognize and kill peptide-loaded target cells within 24 h (Figure S3). Some TCRs conveyed distinct cytotoxic profiles depending on their expression in either $CD4^+$ or $CD8^+$ T cells, as shown in Figure 7A. While both TCR $1C11_{C61}$ $CD4^+$ and $CD8^+$ T cells had killed all target cells after around 12 h, killing of TCR $3H6_{C91}$ $CD4^+$ T cells was delayed and only reached 50% compared with $CD8^+$ T cells (Figure 7A). This slower killing kinetics of $CD4^+$ T cells and reduced cytotoxicity after 24 h was mostly observed for C91-specific TCRs, possibly due to their lower functional avidity (Figures S3 and 7B). Unexpectedly, at the highest effector to target ratio of 1:1, TCR $2F2_{C91}$ showed unspecific killing of the control without peptide (Figures S3 and 7B). Since this unspecific activation was uniquely seen upon co-culture with single MHC II transfectant fibroblasts and never on donor-derived B-LCL, it may be related to cross-reactivity with a fibroblast-derived peptide, potentially enhanced by the artificial over-expression of HLA-DR13 on the single MHC II transfectant target cell line. Overall, nine out of the 14 tested TCRs had a functional avidity high enough to result in prominent killing by $CD4^+$ as well as $CD8^+$ T cells.

DISCUSSION

$CD4^+$ T cells are needed for an efficient and long-lasting antiviral immune response by providing help to $CD8^+$ T cells and inducing B cell differentiation. In this study, we isolated and characterized a panel of MHC II-restricted, HBV-specific TCRs, which can be used to complement MHC I-restricted T cells in T cell therapy or as an experimental tool to study HBV-specific immunity.

Twenty MHC II-restricted TCRs (Table S4) specific for eight peptides derived from the HBV core, envelope, and polymerase proteins could be isolated from donors with acute or resolved HBV infection. Interestingly, both epitopes newly identified to be immunogenic, i.e., C91 and preS9, had not stood out by their prediction score, and TCR $3G3_{preS9}$ was actually restricted toward an MHC II molecule other than those used for initial peptide prediction. The epitope preS9 is particularly intriguing, since $CD4^+$ T cells redirected with the receptor $3G3_{preS9}$ would be expected to prime B cells specific for the large envelope protein, which could give rise to entry-neutralizing antibodies.[38,39] Fourteen of the TCRs recognized more than one HBV genotype variant, making them therapeutically more widely applicable. The failure of, e.g., S36-specific TCRs in recognizing genotypes other than A could be caused by changes in the amino acids affecting the

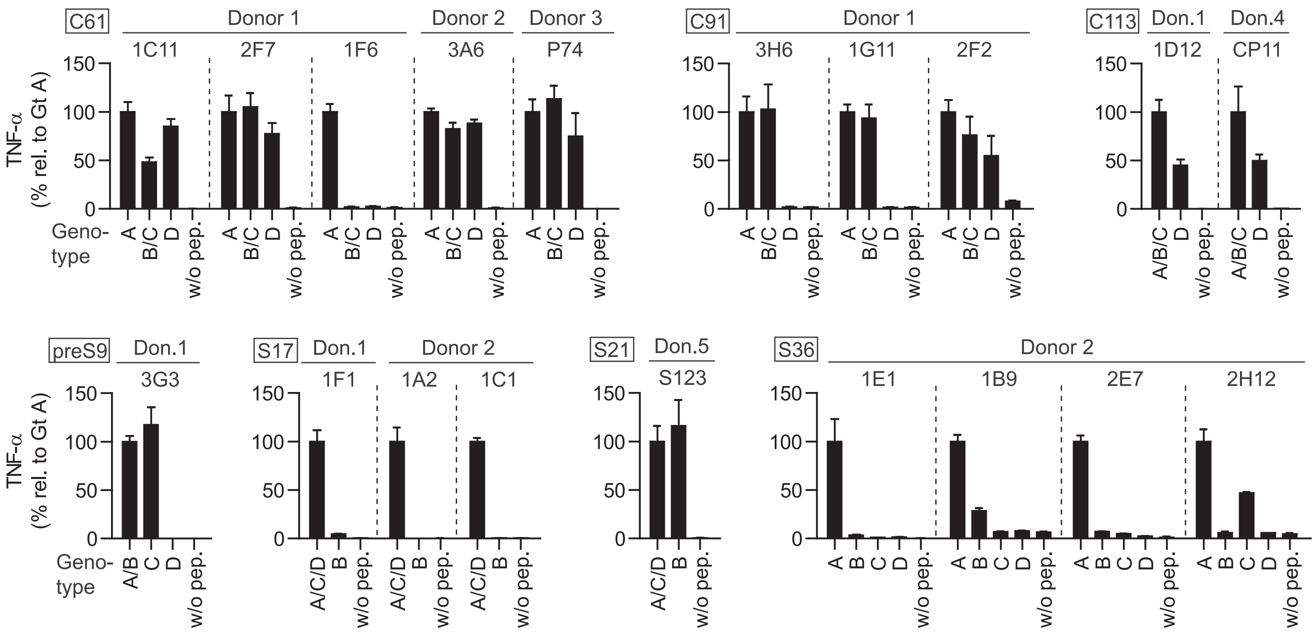

Figure 4. Recognition of peptide variants of major HBV genotypes

TCR-transduced CD4+ T cells were co-cultured at an effector to target cell ratio of 2:1 with HLA-matched B-LCLs pulsed with 1 μM of peptide from HBV genotypes A, B, C, and D. TNF-α secretion was determined via ELISA after 16 h of co-culture and is shown relative to values from co-culture with genotype A (Gt A), since Gt A peptides had been used for initial T cell stimulation and isolation of HBV-specific CD4+ T cell clones. Co-cultures without peptide (w/o pep.) served as negative control. Data points represent mean values ±SD from triplicates. Square boxes at the top left of each graph indicate peptide specificities. TCR $1D4_{P774}$ was not included in this assay, since the P774 peptide is conserved across all four genotypes.

binding core and exposing different residues to the CDR3 regions of the TCR. To elucidate the importance of each amino acid, alanine scans in combination with *in silico* 3D modelling of the peptide:MHC-TCR interaction are warranted.

Besides the binding specificity and strength as defined by each TCR's unique CDR3 region, functionality of a TCR is also influenced by binding and presentation of the respective peptide on the MHC molecule. To address all of these points, T cells expressing MHC II-restricted, HBV-specific TCRs were generated by retroviral transduction. The levels of expression in terms of MFI were consistent for each TCR across several independent transduction experiments and did not correlate with the number of integrates. This suggests that the maximum expression level is a phenotypic feature inherent to each TCR and does not depend on the transduction efficiency. This observation concurs with a recent study classifying TCRs into weak and dominant phenotypes according to their MFI as a measure of surface expression.[40] Thomas *et al.* showed that the variable β-chain TRBV7-9 was over-represented in TCRs with a weak expression phenotype, which holds true for our TCR with the lowest MFI of all, TCR $1D12_{C113}$. Another characteristic of a TCR is its functional avidity, which depends on the affinity of the TCR variable regions to the pMHC complex and correlates with the strength of the T cell response.[41] Virus-specific TCRs are typically of high affinity, since they recognize foreign antigen in a *de novo* encounter and have hence eluded the negative selection process during thymic development.[37] Accordingly, in this study, high values of functional avidity were determined for 15 TCRs with EC_{50} values in a low nanomolar range. Numerous studies in murine models and humans have gathered evidence that CD8+ T cells of higher functional avidity are more efficient in clearing viral infection.[42–44] For CD4+ T cells in particular, fewer conclusive data regarding TCR affinity are available, and it has been suggested that MHC II-restricted TCRs generally display weaker binding affinities in comparison with MHC I-restricted TCRs.[45,46] Hence, with our 20 TCRs covering a range of functional avidities and specificities, we here provide a highly useful resource for studying MHC II-restricted TCR affinities in more detail.

Finally, TCR binding depends on encountering the peptide on the correct MHC molecule. Nine MHC II restrictions were identified during this work, five of which have been linked to beneficial effects with regard to HBV infection, underlining their potential for immunotherapy. For example, studies have shown a correlation with protection against HBV for HLA-DRB1*01:01,[47] DQB1*06:03,[48] DPB1*02:01,[49] and DRB1*13:01.[50–53] In addition, the latter has repeatedly been associated with resolution of HBV infection worldwide.[54–56] HLA-DPB1*15:01, in turn, has been linked to spontaneous HBsAg seroconversion in HBV-infected individuals.[57]

For application of TCRs in T cell therapy, an important aspect to consider is the geographic distribution of MHC molecules as well as

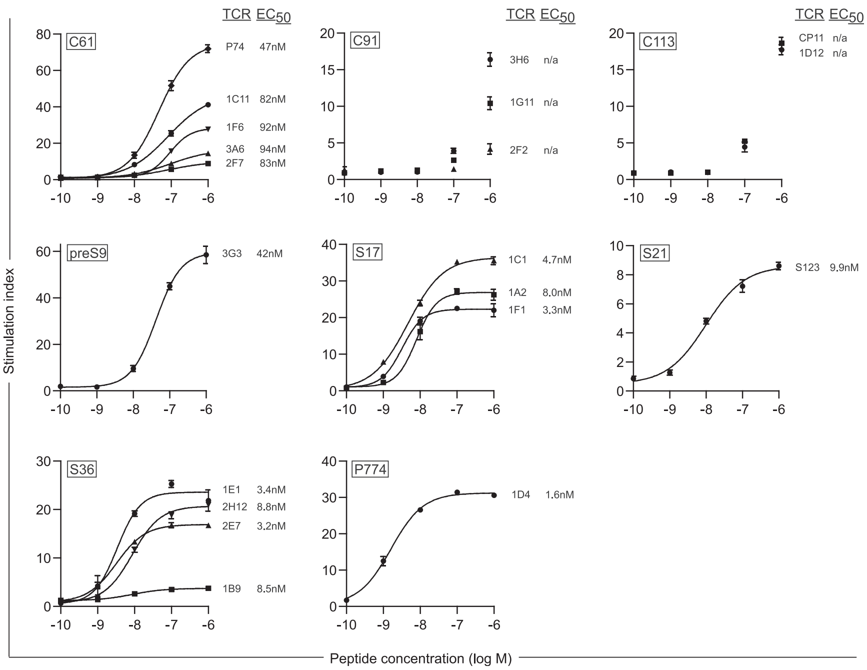

Figure 5. Functional avidity of MHC II-restricted TCRs determined with proliferation assay

TCR-transduced CD4$^+$ T cells were co-cultured at an effector to target cell ratio of 2:1 with HLA-matched B-LCLs titrating the amount of target peptide from 1 μM to 100 pM. Proliferation was assessed through integration of ^{3}H-thymidine after 72 h of co-culture. Results are expressed as stimulation index, i.e., cpm of a stimulated sample divided by cpm of the unloaded control. Data points represent mean values ±SD from triplicates. All indicated EC_{50} values were calculated with a non-linear dose-response ordinary fit. EC_{50} values for C91- and C113-specific TCRs could not be calculated (n/a) because they did not reach a plateau of proliferation at the highest peptide concentration. Square boxes at the top left of each graph indicate peptide specificities.

the local prevalence of HBV infection. Out of our isolated TCRs, the HLA-DPB1*02:01-restricted ones would have the broadest therapeutic applicability, given this allele is common with, e.g., a frequency of 23% in China's Han population.[58] Interestingly, TCR 1C11$_{C61}$ showed a promiscuous but specific binding behavior toward DRB1*01:01 and DRB3*02:02. Such TCR promiscuity has been reported previously in the form of a single target peptide being recognized on different MHC molecules,[59,60] and can potentially broaden the number of patients eligible for T cell therapy with a given receptor. The number of different MHC restrictions covered by our library is also advantageous for further studies of, e.g., immune cell interactions and immunomodulators that supposedly increase MHC presentation, or in pre-clinical models of HBV infection and HBV-targeted T cell therapy. As such, our TCRs could be used with MHC II-expressing HBV-susceptible cell lines (e.g., HepaRG, DRB1*07:01$^+$, DPB1*04:01$^+$), "professional" APC lines that take up HBV (e.g., THP-1,[61] DRB1*01:01$^+$, DQB1*05:01$^+$, DPB1*02:01$^+$), or *in vivo* in AAV-HBV infected mice featuring HLA-A2 and -DR1[34] or -DP4[35] expression.

T cell signaling after engagement of pMHC and TCR is supported by CD4 or CD8 co-receptor binding to the respective MHC molecule.[62] All the MHC II-restricted TCRs characterized throughout this study were equally able to activate CD8$^+$ T cells regardless of their different functional avidities. This argues for a minor role of CD4 regarding the actual TCR-pMHC interaction,[63,64] but rather a role for enhancing T cell sensitivity via its strong intracellular association with tyrosine kinases in CD4$^+$ T cells.[65,66] The introduction of MHC II-restricted TCRs in CD8$^+$ T cells has rarely been attempted. One group compared CD4$^+$ and CD8$^+$ T cells transduced with an HLA-DQ5-restricted TCR targeting the dead box RNA helicase Y *in vitro*. They reported similar killing capacity of both transduced CD4$^+$ and CD8$^+$ T cells, but cytokine secretion was significantly diminished in CD8$^+$ T cells and only slightly increased after co-introduction of the CD4 co-receptor.[67] Ample opportunity remains to further investigate the nature and potential of MHC II-restricted CD8$^+$ T cells based on the panel of TCRs presented in this study.

Both CD4$^+$ and CD8$^+$ T cells transduced with MHC II-restricted TCRs were polyfunctional and produced varying amounts of cytokines, such as TNF-α, IFN-γ, and IL-2. Thus, CD4$^+$ T cells showed rather a T_H1-phenotype,[68] most likely induced by the anti-CD3/anti-CD28 and IL-2 stimulation mimicking antigen encounter during the transduction procedure. IFN-γ and TNF-α were shown to interfere with the stability of HBV covalently closed circular DNA (cccDNA) via nuclear deaminases.[69] This cytokine-mediated HBV inhibition was also observed upon the addition of HBV-specific redirected T cells without direct cell-cell contact.[69] Therefore, cytokine-secreting CD4$^+$ T cells could also contribute directly to the antiviral effect of adoptive T cell therapy. Given that especially IFN-γ-producing S-specific CD4$^+$ T cells but not TNF-α-producing CD4$^+$ T cells were shown to correlate with HBV clearance in CHB patients,[19] artificial generation of such cells with our TCRs would be particularly interesting.

Furthermore, CD4$^+$ T cells transduced with MHC II-restricted TCRs secreted varying amounts of GrzB and selectively killed MHC II-expressing peptide-pulsed target cells. The percentage of GrzB$^+$ CD4$^+$ T cells was consistently lower compared with CD8$^+$ T cells, which could be explained by CD8$^+$ T cells as professional cytotoxic cells storing more GrzB intracellularly.[70] The presence and characteristics of CD4$^+$ cytotoxic T lymphocytes (CTLs) have been described for murine or human viral infection.[71,72] Initially, they were believed to be an artifact of long-term *in vitro* cell culture but, over time, numerous studies also reported their existence *ex vivo*.[73–76] In humans, they were associated with a protective role in influenza[77] and HIV

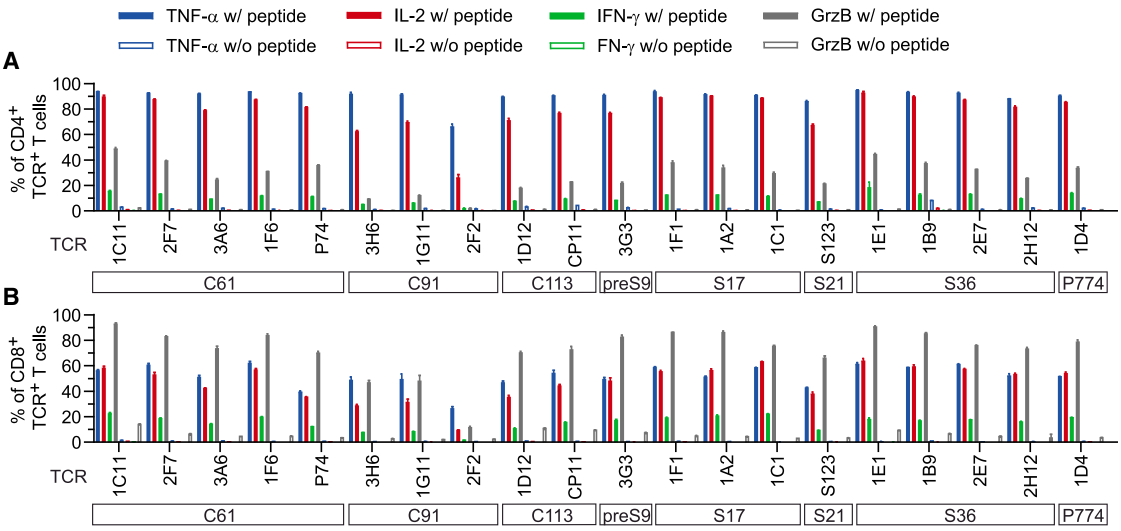

Figure 6. Cytokine and GrzB secretion of TCR-transduced CD4+ or CD8+ T cells

CD4+ and CD8+ T cells were co-transduced and then separated by positive selection through magnetic-activated cell sorting prior to the experiment with purities ≥98%. TCR-transduced CD4+ (A) or CD8+ (B) T cells were co-cultured with HLA-matched B-LCLs, then pulsed with 1 μM of target peptide (w/ peptide). Brefeldin A was added 1 h after co-culture start to retain cytokines in the endoplasmic reticulum (ER). TNF-α (blue bars),IL-2 (red bars), IFN-γ (green bars) and GrzB (gray bars) were measured via intracellular cytokine staining and flow cytometry after 14 h of co-culture in CD4+ TCR+ or CD8+ TCR+ subsets, respectively. Co-cultures without peptide (w/o peptide) served as negative control (empty bars in respective colors). Data points represent mean values ±SD from triplicates. Square boxes below TCRs indicate peptide specificities.

infection.[78] Little is known, however, with regard to their role in HBV infection. A study comparing individuals with chronic HBV, hepatitis C virus (HCV), or HBV/hepatitis D virus (HDV) (co-)infection with healthy controls showed elevated numbers of CD4+ T cells expressing perforin *ex vivo*, with particularly high rates in HBV/HDV co-infected patients.[79] In addition, perforin expression was most pronounced in patients with advanced hepatitis and was linked to liver damage.[79] This has led to the hypothesis that CD4+ CTLs in chronic hepatitis may in fact contribute to immunopathology.[71] The TCRs described in our study could help elucidate the role of CD4+ CTLs in chronic hepatitis and HBV-induced HCC in more detail.

In this regard, it is important to define potential target cells expressing MHC II before applying cytotoxic HBV-specific T cells in adoptive T cell therapy. Human hepatocytes are not thought to express MHC II under normal conditions, and although some upregulation has been proposed to occur during inflammation,[80] solid and contemporary data on MHC II expression during viral hepatitis are missing. In transgenic mice overexpressing the transcriptional regulator of MHC II, hepatocytes were shown to function as APCs, specifically activating CD4+ T cells.[81] Professional APCs like dendritic cells are presumably protected from cytotoxic CD4+ CTL activity given that they express the GrzB inhibitor SerpinB9.[82] In addition, upon activation by dendritic cells, T cells rapidly express cytotoxic T lymphocyte–associated protein 4 (CTLA-4), which then competes with CD28 for interaction with CD80 on the APC. This prevents the formation of an effective immunological synapse and ultimately protects the APC from becoming a T cell target.[83] Liver-resident Kupffer cells and liver sinusoidal endothelial cells, which upregulate MHC II expression in response to pro-inflammatory cytokines such as IFN-γ, may well be protected by similar mechanisms given their antigen-presenting function and CD80 expression.[84] It seems reasonable to assume that major damage to APCs in the liver is unlikely. Efficient targeting of MHC II-expressing hepatocytes by CD4+ CTLs would be unexpected and their direct antiviral activity remains to be determined. Especially in the treatment of HBV-induced HCC, their role may even be beneficial, as, for example, CD4+ T cells with cytotoxic activity were shown to induce tumor rejection in a melanoma model.[85]

Taken together, we here described a library of 20 MHC II-restricted TCRs specific for different HBV antigens. An in-depth characterization defined their MHC restriction, expression levels, recognition of different genotypes, and intracellularly processed antigen, as well as their functional avidity. With regard to these qualities, C61-specific TCRs (e.g., $1C11_{C61}$ and $P74_{C61}$) could be considered favorable for their further evaluation regarding their potential in adoptive T cell therapy. S- and L-specific TCRs (e.g., $1F1_{S17}$ and $3G3_{preS9}$) will be especially interesting for the investigation of B cell responses and induction of HBV-neutralizing antibodies. This study lays the groundwork for the further use of MHC II-restricted TCRs in T cell therapy of chronic HBV infection and HBV-induced HCC and provides a valuable tool for the study of CD4+ T cells and their role in HBV infection and cure.

MATERIALS AND METHODS

T cell stimulation

PBMC from donors with resolved or acute HBV infection were isolated via a standard densitygradient (Biocoll, Merck). Informed consent in writing was obtained from each patient. PBMC from donor 1

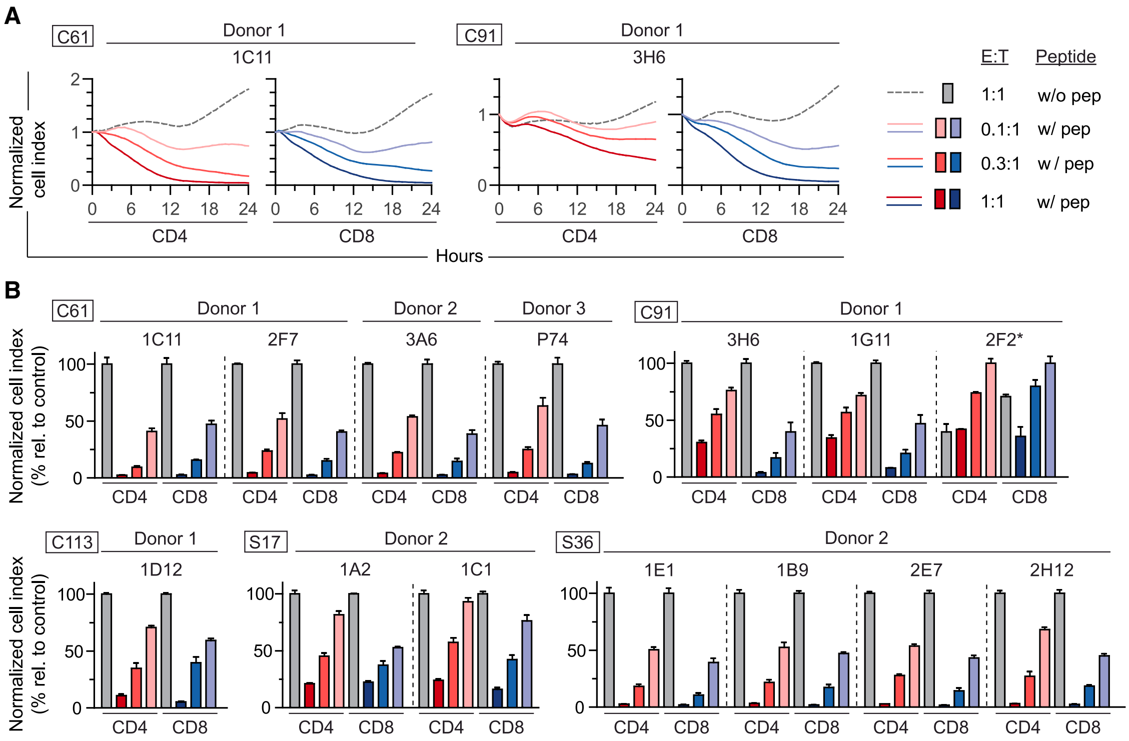

Figure 7. Cytotoxic capacity of TCR-transduced CD4$^+$ and CD8$^+$ T cells

CD4$^+$ and CD8$^+$ T cells were co-transduced and then separated by positive selection through magnetic-activated cell sorting to purities of ≥98%. TCR-transduced CD4$^+$ (red) or CD8$^+$ (blue) T cells were co-cultured for 24 h with single MHC II transfectant fibroblasts pulsed with 1 μM of peptide (w/ pep) at an effector to target (E:T) cell ratio of 1:1 (dark color), 0.3:1 (medium color), or 0.1:1 (light color) or without peptide (w/o pep) at E:T ratio 1:1 (gray). Cytotoxicity was assessed via the adherence of target cells measured through electrical impedance and is given as a cell index normalized to the starting point of each co-culture. Considering the technical requirements of this assay, only TCRs were included, for which adherent single MHC II transfectant fibroblasts were available. (A) Cytotoxicity kinetics for exemplary TCRs $1C11_{C61}$ and $3H6_{C91}$. Data points were acquired every 30 min and represent mean values from triplicates. (B) Endpoint cytotoxicity after 24 h of co-culture for exemplary TCRs. The normalized cell index is given relative to killing of target cells without peptide at the highest E:T of 1:1, with the exception of TCR $2F2_{C91}$(*), where samples with peptide at an E:T of 0.1:1 are set to 100%. Data points represent mean values ±SD from triplicates. Square boxes at the top left of each graph indicate peptide specificities.

and donor 2 were stimulated with 1 μM of single peptides (Peptides & Elephants or JPT Peptide Technologies, Table S1) for 14 days at 1 × 10^6/well in a 24-well plate and expanded when necessary, and 10 ng/mL IL-7 and IL-15 (both from Peprotech) were added on day 0. 50 U/mL IL-2 (Proleukin, Novartis Pharmaceuticals) was added on day 1 and to fresh medium after expansion. PBMCs were kept in T cell medium with human serum: RPMI, 10% human serum (own production from male, healthy donors), 1% penicillin-streptomycin (pen/strep), 1% glutamine, 1% sodium pyruvate, 1% non-essential amino acids (NEAA), 10 mM HEPES, and 16.6 μg/mL gentamicin (all from Thermo Fisher Scientific). Minor adjustments were applied to the stimulation conditions for donors 3 to 5 as described in the supplemental methods.

T cell cloning

T cells were restimulated with the respective peptide (1 μM) and stained with the TNF-α and/or IFN-γ secretion assay (Miltenyi Biotec) according to the manufacturer's instructions as well as anti-human CD4-APC (eBioscience, Thermo Fisher Scientific) and anti-human CD8-PB (BioLegend). TNF-α$^+$ CD4$^+$ T cells were enriched using a fluorescence-activated cell sorting (FACS) Aria III (BD) or a MoFlo XDP cell sorter (Beckmann Coulter), and 0.5 cells/well were seeded in 96-well round-bottom plates containing 7.5 × 10^4 irradiated heterologous PBMCs (35 Gy), 1 × 10^4 B-LCLs (50 Gy), 50 IU/mL IL-2, and 30 ng/mL OKT-3 antibody (eBioscience, Thermo Fisher Scientific). HBV-specific T cells clones were identified as described in the supplemental methods. For expansion, selected HBV-specific T cell clones were moved to a 12-well plate containing 5 × 10^6 irradiated PBMCs, 1 × 10^6 irradiated B-LCLs, and 30 ng/mL OKT-3 antibody. Then 50 U/mL IL-2 were supplemented on days 1, 5, 8, and 11 and split to two wells when necessary. The TCR chains of HBV-specific clones were analyzed and cloned as described in the supplemental methods.

Retroviral transduction of T cells

T cells were enriched using human T activator CD3/CD28 Dynabeads (Thermo Fisher Scientific) and pre-stimulated for 2 days in T cell medium with FBS: RPMI, 10% FBS, 1% pen/strep, 1% glutamine, 1% sodium pyruvate, 1% NEAA, 10 mM HEPES, 16.6 μg/mL gentamicin (all from Thermo Fisher Scientific), supplemented with

300 U/mL IL-2. The 0.45 μm-filtered retrovirus cell culture supernatant from stable producer cell lines was centrifuged at 2,000 × g, 32°C for 2 h on non-tissue culture-treated plates (Corning) coated with 20 μg/mL RetroNectin for 2 h (Takara). Retrovirus cell culture supernatant was removed and T cells were spinoculated onto the retrovirus-coated plate at 1,000 × g for 10 min. A second transduction was performed after 24 h. TCR expression was determined by flow cytometry. Staining was done for 30 min on ice in the dark, using the primary antibodies anti-human CD4-APC , anti-human CD8-PB, and anti-mouse TCRβ-PE (BD Biosciences), diluted in PBS with 0.1% BSA (Sigma-Aldrich). Cells were analyzed using a CytoFLEX S (Beckman Coulter) and data were analyzed with FlowJo 10.4 software. To determine the number of integrates per cell, genomic DNA from transduced T cells was isolated with a DNA tissue extraction kit (MACHEREY-NAGEL). The vector copy number was measured in a multiplex qPCR of viral woodchuck hepatitis virus postregulatory element relative to the genomic *PTBP2* as described elsewhere.[86] The protocol and plasmid standard were kindly provided by the Hannover Medical School, Institute of Experimental Hematology.

Co-cultures with B-LCLs

B-LCLs were cultivated in RPMI full medium: RPMI with 10% FBS, 1% pen/strep, 1% glutamine, 1% sodium pyruvate, and 1% NEAA (all from Thermo Fisher Scientific). Prior to the experiment, B-LCLs were irradiated with 50 Gy and loaded with 1 μM or decreasing amounts of peptide for 2 h at 37°C and then washed twice with PBS. For recognition of physiologically processed antigen, B-LCLs were pre-incubated with the HBV core or small envelope protein (genotype A, kindly provided by the Centro de Ingeniería Genética y Biotecnología de Cuba) at 1 or 10 μg/mL. TCR^+ T cells (1×10^5) were incubated with 5×10^4 peptide-loaded B-LCLs at 37°C. TNF-α secretion via ELISA (BD) was measured after 16 h from supernatants. For intracellular cytokine staining, 2.5 μg/mL Brefeldin A (Sigma-Aldrich) was added 1 h after co-culture start, and staining was performed after 14 h, using the Fixation/Permeabilization Solution kit (BD Biosciences) and the following antibodies: live/dead Fixable Aqua stain (Invitrogen, Thermo Fisher Scientific), anti-human CD4-PerCP (clone SK3, BioLegend), anti-human CD8-FITC (clone RPA-T8, Invitrogen, Thermo Fisher Scientific), anti-mouse TCRβ-PE (clone H57-597, BD Biosciences), anti-human IFN-γ-AF700 (clone B27, BD Biosciences), anti-human TNF-α-APC (clone Mab11, BioLegend), anti-human IL-2-PE-Cy7 (clone MQ1-17H12, Invitrogen, Thermo Fisher Scientific), and anti-human GrzB-PB (clone N4TL33, Invitrogen, Thermo Fisher Scientific).

For proliferation analysis, after 72 h of co-culture 1 μCi ^{3}H-thymidine was added per well and incubated for another 16 h at 37°C. The cells were then transferred onto a Filtermat A membrane using a Filtermat-96 Harvester. After a drying period of 5 h at 37°C, the membranes were placed into plastic scintillation sleeves with approximately 1 mL of BetaPlate scintillation fluid. Counts per minute (cpm) were evaluated in a MicroBeta TriLux 1450 scintillation counter (all from PerkinElmer). EC_{50} values were calculated with a non-linear dose-response ordinary fit with Prism8 (GraphPad). R^2 values were consistently ≥0.99 with the exception of TCR $1E1_{S36}$ (0.97).

Co-culture with single MHC II transfectant target cells

Single MHC II transfectant target cells[31] were kindly provided by Alessandro Sette, La Jolla Institute of Immunology, San Diego, United States. These DAP3-based fibroblasts or RM3-based B-lymphoblasts were grown in RPMI full medium and maintained under selection pressure with 200 μg/mL Geneticin or 700 μg/mL Geneticin and 12 μg/mL blasticidin (all from Thermo Fisher Scientific), respectively. To increase MHC expression prior to co-culture, single MHC II transfectants were stimulated with 100 μg/mL sodium butyrate (Sigma-Aldrich) overnight at 37°C in their respective culture media. DAP3-based adherent fibroblasts were seeded with 5×10^4 cells/well in flat-bottom 96-well plates, loaded with 1 μM peptide for 4 h at 37°C and then washed twice with PBS. Raji-based suspension B-LCLs were loaded in V-bottom plates with 1 μM of peptide for 4 h at 37°C, washed twice with PBS, and subsequently seeded into round-bottom 96-well plates with 5×10^4 cells/well. Transduced T cells were added with 1×10^5 cells/well and incubated overnight at 37°C. After 16 h, supernatants were stored at -20°C and TNF-α secretion was measured by ELISA at a later time point (Invitrogen, Thermo Fisher Scientific).

Real-time cytotoxicity measurement

DAP3-based fibroblasts were prepared for co-culture with T cells as described above, seeded onto 96-well electronic microtiter plates (ACEA Biosciences) with 5×10^4/well, loaded with 1 μM of peptide for 4 h at 37°C, and washed twice with PBS. $CD4^+$ or $CD8^+$ TCR^+ T cells were added at different effector to target ratios (1:1, 0.3:1, 0.1:1). The impedance, which reflects adherence of the target cells to the bottom of the plate, was measured every 30 min using an xCELLigence SP real-time cell analyzer (ACEA Biosciences).

Study approval

The use of volunteer PBMCs was approved by the local ethics board of the University Hospital rechts der Isar, Munich, and the ethics committee of the University of LMU, Munich. Written informed consent was obtained from all blood donors.

SUPPLEMENTAL INFORMATION

Supplemental information can be found online at https://doi.org/10.1016/j.omtm.2021.10.012.

ACKNOWLEDGMENTS

We thank Ali Afzali and Thomas Korn of the Department of Neurology at the University Hospital rechts der Isar, Technical University of Munich, for help with the proliferation assay; Michael Rothe and Tobias Mätzig of the Institute of Experimental Hematology at Hannover Medical School for providing the protocol and plasmid standard to determine the vector copy number; and Bijan Raziorrouh of the Medical Department II and Institute for Immunology, Hospital of the Ludwig-Maximilians-University (LMU) Munich, , for assistance with sample acquisition. We are grateful to John Sidney of

the Center for Infectious Disease and Vaccine Research at the La Jolla Institute for Immunology for help with the MHC-ligand binding assay and for critical reading of the manuscript.

The project was in part funded by the German Research Foundation (Deutsche Forschungsgemeinschaft [DFG]) via the Collaborative Research Center, TRR338 project B07 to U.P. and TRR 179 (project no. 272983813) to M.S.; the German Center for Infection Research to U.P. and K. Wisskirchen (DZIF, TTU Hepatitis 05.806); the DFG through the TUM International Graduate School of Science and Engineering (IGSSE), GSC 81 to S.S.; and NIH NIAID (United States) contract no. 75N9301900065 to A.S.

AUTHOR CONTRIBUTIONS

S.S., U.P., and K. Wisskirchen designed experiments. S.S. and K. Wisskirchen wrote the manuscript. S.S., M.H., W.M., and K. Witter performed experiments. S.S., E.M., U.P., and K. Wisskirchen analyzed data. C.Z. and P.K. provided cellular material and know-how. A.S. and M.S. provided critical reagents and/or infrastructure. U.P. provided financial resources and key infrastructure. All authors discussed the data and provided input for writing the manuscript.

DECLARATION OF INTERESTS

K. Wisskirchen is partially employed by SCG Cell Therapy GmbH and holds shares of SCG Cell Therapy Pte. Ltd. U.P. holds shares and received research funding from SCG Cell Therapy Pte. Ltd. The other authors declare no competing interests.

REFERENCES

1. WHO. (2021). Hepatitis B, Fact Sheet, https://www.who.int/news-room/fact-sheets/detail/hepatitis-b.
2. Rehermann, B. (2013). Pathogenesis of chronic viral hepatitis: differential roles of T cells and NK cells. Nat. Med. *19*, 859–868.
3. Bohne, F., and Protzer, U. (2007). Adoptive T-cell therapy as a therapeutic option for chronic hepatitis B. J. Viral Hepat. *14* (*Suppl 1*), 45–50.
4. Ilan, Y., Nagler, A., Adler, R., Naparstek, E., Or, R., Slavin, S., Brautbar, C., and Shouval, D. (1993). Adoptive transfer of immunity to hepatitis B virus after T cell-depleted allogeneic bone marrow transplantation. Hepatology *18*, 246–252.
5. Lau, G.K., Lok, A.S., Liang, R.H., Lai, C.L., Chiu, E.K., Lau, Y.L., and Lam, S.K. (1997). Clearance of hepatitis B surface antigen after bone marrow transplantation: role of adoptive immunity transfer. Hepatology *25*, 1497–1501.
6. Tan, A.T., and Schreiber, S. (2020). Adoptive T-cell therapy for HBV-associated HCC and HBV infection. Antivir. Res. *176*, 104748.
7. Bohne, F., Chmielewski, M., Ebert, G., Wiegmann, K., Kurschner, T., Schulze, A., Urban, S., Kronke, M., Abken, H., and Protzer, U. (2008). T cells redirected against hepatitis B virus surface proteins eliminate infected hepatocytes. Gastroenterology *134*, 239–247.
8. Wisskirchen, K., Metzger, K., Schreiber, S., Asen, T., Weigand, L., Dargel, C., Witter, K., Kieback, E., Sprinzl, M.F., Uckert, W., et al. (2017). Isolation and functional characterization of hepatitis B virus-specific T-cell receptors as new tools for experimental and clinical use. PLoS One *12*, e0182936.
9. Krebs, K., Bottinger, N., Huang, L.R., Chmielewski, M., Arzberger, S., Gasteiger, G., Jager, C., Schmitt, E., Bohne, F., Aichler, M., et al. (2013). T cells expressing a chimeric antigen receptor that binds hepatitis B virus envelope proteins control virus replication in mice. Gastroenterology *145*, 456–465.
10. Wisskirchen, K., Kah, J., Malo, A., Asen, T., Volz, T., Allweiss, L., Wettengel, J.M., Lütgehetmann, M., Urban, S., Bauer, T., et al. (2019). T cell receptor grafting allows virological control of Hepatitis B virus infection. J. Clin. Invest. *129*, 2932–2945.
11. Bertoletti, A., and Bert, N.L. (2018). Immunotherapy for chronic hepatitis B virus infection. Gut Liver *12*, 497–507. https://doi.org/10.5009/gnl17233.
12. Ferrari, C., Penna, A., Bertoletti, A., Valli, A., Antoni, A.D., Giuberti, T., Cavalli, A., Petit, M.A., and Fiaccadori, F. (1990). Cellular immune response to hepatitis B virus-encoded antigens in acute and chronic hepatitis B virus infection. J. Immunol. *145*, 3442–3449.
13. Penna, A., Del Prete, G., Cavalli, A., Bertoletti, A., D'Elios, M.M., Sorrentino, R., D'Amato, M., Boni, C., Pilli, M., Fiaccadori, F., et al. (1997). Predominant T-helper 1 cytokine profile of hepatitis B virus nucleocapsid-specific T cells in acute self-limited hepatitis B. Hepatology *25*, 1022–1027.
14. Asabe, S., Wieland, S.F., Chattopadhyay, P.K., Roederer, M., Engle, R.E., Purcell, R.H., and Chisari, F.V. (2009). The size of the viral inoculum contributes to the outcome of hepatitis B virus infection. J. Virol. *83*, 9652–9662.
15. Borst, J., Ahrends, T., Bąbała, N., Melief, C.J.M., and Kastenmüller, W. (2018). CD4(+) T cell help in cancer immunology and immunotherapy. Nat. Rev. Immunol. *18*, 635–647.
16. Swain, S.L., McKinstry, K.K., and Strutt, T.M. (2012). Expanding roles for $CD4^+$ T cells in immunity to viruses. Nat. Rev. Immunol. *12*, 136–148.
17. Murphy, K., and Weaver, C. (2016). Janeway's Immunobiology, *xx*, 9th edition (Garland Science/Taylor & Francis Group, LLC), p. 904.
18. Tan, A., Koh, S., and Bertoletti, A. (2015). Immune response in hepatitis B virus infection. Cold Spring Harb Perspect. Med. *5*, a021428.
19. Wang, H., Luo, H., Wan, X., Fu, X., Mao, Q., Xiang, X., Zhou, Y., He, W., Zhang, J., Guo, Y., et al. (2020). TNF-α/IFN-γ profile of HBV-specific CD4 T cells is associated with liver damage and viral clearance in chronic HBV infection. J. Hepatol. *72*, 45–56.
20. Jacobi, F.J., Wild, K., Smits, M., Zoldan, K., Csernalabics, B., Flecken, T., Lang, J., Ehrenmann, P., Emmerich, F., Hofmann, M., et al. (2019). OX40 stimulation and PD-L1 blockade synergistically augment HBV-specific CD4 T cells in patients with HBeAg-negative infection. J. Hepatol. *70*, 1103–1113.
21. Kamphorst, A.O., and Ahmed, R. (2013). CD4 T-cell immunotherapy for chronic viral infections and cancer. Immunotherapy *5*, 975–987.
22. Tay, R.E., Richardson, E.K., and Toh, H.C. (2020). Revisiting the role of CD4(+) T cells in cancer immunotherapy-new insights into old paradigms. Cancer Gene Ther. *28*, 5–17. https://doi.org/10.1038/s41417-020-0183-x.
23. Sommermeyer, D., Hudecek, M., Kosasih, P.L., Gogishvili, T., Maloney, D.G., Turtle, C.J., and Riddell, S.R. (2016). Chimeric antigen receptor-modified T cells derived from defined CD8+ and CD4+ subsets confer superior antitumor reactivity in vivo. Leukemia *30*, 492–500.
24. Wang, D., Aguilar, B., Starr, R., Alizadeh, D., Brito, A., Sarkissian, A., Ostberg, J.R., Forman, S.J., and Brown, C.E. (2018). Glioblastoma-targeted CD4+ CAR T cells mediate superior antitumor activity. JCI Insight *3*, e99048. https://doi.org/10.1172/jci.insight.99048.
25. Ostroumov, D., Fekete-Drimusz, N., Saborowski, M., Kühnel, F., and Woller, N. (2018). CD4 and CD8 T lymphocyte interplay in controlling tumor growth. Cell Mol Life Sci *75*, 689–713.
26. Bos, R., and Sherman, L.A. (2010). CD4+ T-cell help in the tumor milieu is required for recruitment and cytolytic function of CD8+ T lymphocytes. Cancer Res. *70*, 8368–8377.
27. Qin, Z., and Blankenstein, T. (2000). CD4+ T cell–mediated tumor rejection involves inhibition of angiogenesis that is dependent on IFN gamma receptor expression by nonhematopoietic cells. Immunity *12*, 677–686.
28. Hunder, N.N., Wallen, H., Cao, J., Hendricks, D.W., Reilly, J.Z., Rodmyre, R., Jungbluth, A., Gnjatic, S., Thompson, J.A., and Yee, C. (2008). Treatment of metastatic melanoma with autologous CD4+ T cells against NY-ESO-1. N. Engl. J. Med. *358*, 2698–2703.
29. Mercier-Letondal, P., Marton, C., Deschamps, M., Ferrand, C., Vauchy, C., Chenut, C., Baguet, A., Adotévi, O., Borg, C., Galaine, J., et al. (2018). Isolation and characterization of an HLA-DRB1*04-restricted HPV16-E7 T cell receptor for cancer immunotherapy. Hum. Gene Ther. *29*, 1202–1212.
30. Poncette, L., Chen, X., Lorenz, F.K., and Blankenstein, T. (2019). Effective NY-ESO-1-specific MHC II-restricted T cell receptors from antigen-negative hosts enhance tumor regression. J. Clin. Invest. *129*, 324–335.

31. McKinney, D.M., Southwood, S., Hinz, D., Oseroff, C., Arlehamn, C.S., Schulten, V., Taplitz, R., Broide, D., Hanekom, W.A., Scriba, T.J., et al. (2013). A strategy to determine HLA class II restriction broadly covering the DR, DP, and DQ allelic variants most commonly expressed in the general population. Immunogenetics *65*, 357–370.

32. Chisari, F.V., and Ferrari, C. (1995). Hepatitis B virus immunopathogenesis. Annu. Rev. Immunol. *13*, 29–60.

33. Raziorrouh, B., Heeg, M., Kurktschiev, P., Schraut, W., Zachoval, R., Wendtner, C., Wächtler, M., Spannagl, M., Denk, G., Ulsenheimer, A., et al. (2014). Inhibitory phenotype of HBV-specific CD4+ T-cells is characterized by high PD-1 expression but absent coregulation of multiple inhibitory molecules. PLoS One *9*, e105703.

34. Pajot, A., Michel, M.L., Fazilleau, N., Pancré, V., Auriault, C., Ojcius, D.M., Lemonnier, F.A., and Lone, Y.C. (2004). A mouse model of human adaptive immune functions: HLA-A2.1-/HLA-DR1-transgenic H-2 class I-/class II-knockout mice. Eur. J. Immunol. *34*, 3060–3069.

35. Ru, Z., Xiao, W., Pajot, A., Kou, Z., Sun, S., Maillere, B., Zhao, G., Ojcius, D.M., Lone, Y.C., and Zhou, Y. (2012). Development of a humanized HLA-A2.1/DP4 transgenic mouse model and the use of this model to map HLA-DP4-restricted epitopes of HBV envelope protein. PLoS One *7*, e32247.

36. Velkov, S., Ott, J.J., Protzer, U., and Michler, T. (2018). The global hepatitis B virus genotype distribution approximated from available genotyping data. Genes (Basel) *9*, 495. https://doi.org/10.3390/genes9100495.

37. Aleksic, M., Liddy, N., Molloy, P.E., Pumphrey, N., Vuidepot, A., Chang, K.M., and Jakobsen, B.K. (2012). Different affinity windows for virus and cancer-specific T-cell receptors: implications for therapeutic strategies. Eur. J. Immunol. *42*, 3174–3179.

38. Gerlich, W.H. (2015). Prophylactic vaccination against hepatitis B: achievements, challenges and perspectives. Med. Microbiol. Immunol. *204*, 39–55.

39. Neurath, A.R., Seto, B., and Strick, N. (1989). Antibodies to synthetic peptides from the preS1 region of the hepatitis B virus (HBV) envelope (env) protein are virus-neutralizing and protective. Vaccine *7*, 234–236.

40. Thomas, S., Mohammed, F., Reijmers, R.M., Woolston, A., Stauss, T., Kennedy, A., Stirling, D., Holler, A., Green, L., Jones, D., et al. (2019). Framework engineering to produce dominant T cell receptors with enhanced antigen-specific function. Nat. Commun. *10*, 4451.

41. Zhong, S., Malecek, K., Johnson, L.A., Yu, Z., Vega-Saenz de Miera, E., Darvishian, F., McGary, K., Huang, K., Boyer, J., Corse, E., et al. (2013). T-cell receptor affinity and avidity defines antitumor response and autoimmunity in T-cell immunotherapy. Proc. Natl. Acad. Sci. U S A *110*, 6973–6978.

42. Almeida, J.R., Price, D.A., Papagno, L., Arkoub, Z.A., Sauce, D., Bornstein, E., Asher, T.E., Samri, A., Schnuriger, A., Theodorou, I., et al. (2007). Superior control of HIV-1 replication by CD8+ T cells is reflected by their avidity, polyfunctionality, and clonal turnover. J. Exp. Med. *204*, 2473–2485.

43. Derby, M., Alexander-Miller, M., Tse, R., and Berzofsky, J. (2001). High-avidity CTL exploit two complementary mechanisms to provide better protection against viral infection than low-avidity CTL. J. Immunol. *166*, 1690–1697.

44. Viganò, S., Utzschneider, D.T., Perreau, M., Pantaleo, G., Zehn, D., and Harari, A. (2012). Functional avidity: a measure to predict the efficacy of effector T cells? Clin. Dev. Immunol. *2012*, 153863.

45. Nakatsugawa, M., Rahman, M.A., Yamashita, Y., Ochi, T., Wnuk, P., Tanaka, S., Chamoto, K., Kagoya, Y., Saso, K., Guo, T., et al. (2016). CD4(+) and CD8(+) TCRβ repertoires possess different potentials to generate extraordinarily high-avidity T cells. Sci. Rep. *6*, 23821.

46. van der Merwe, P.A., and Davis, S.J. (2003). Molecular interactions mediating T cell antigen recognition. Annu. Rev. Immunol. *21*, 659–684.

47. Matei, H.V., Vica, M.L., and Siserman, C.V. (2018). Association between HLA class II alleles and hepatitis B virus infection in Transylvania, Romania. Immunol. Invest. *47*, 735–744.

48. Ou, G., Xu, H., Yu, H., Liu, X., Yang, L., Ji, X., Wang, J., and Liu, Z. (2018). The roles of HLA-DQB1 gene polymorphisms in hepatitis B virus infection. J. Transl Med. *16*, 362.

49. Nishida, N., Sawai, H., Kashiwase, K., Minami, M., Sugiyama, M., Seto, W.K., Yuen, M.F., Posuwan, N., Poovorawan, Y., Ahn, S.H., et al. (2014). New susceptibility and resistance HLA-DP alleles to HBV-related diseases identified by a trans-ethnic association study in Asia. PLoS One *9*, e86449.

50. Ahn, S.H., Han, K.H., Park, J.Y., Lee, C.K., Kang, S.W., Chon, C.Y., Kim, Y.S., Park, K., Kim, D.K., and Moon, Y.M. (2000). Association between hepatitis B virus infection and HLA-DR type in Korea. Hepatology *31*, 1371–1373.

51. Höhler, T., Gerken, G., Notghi, A., Lubjuhn, R., Taheri, H., Protzer, U., Löhr, H.F., Schneider, P.M., Meyer zum Büschenfelde, K.H., and Rittner, C. (1997). HLA-DRB1*1301 and *1302 protect against chronic hepatitis B. J. Hepatol. *26*, 503–507.

52. Li, X., Liu, W., Wang, H., Jin, X., Fang, S., Shi, Y., Liu, Z., Zhang, S., and Yang, S. (2012). The influence of HLA alleles and HBV subgenotyes on the outcomes of HBV infections in northeast China. Virus Res. *163*, 328–333.

53. Thursz, M.R., Kwiatkowski, D., Allsopp, C.E., Greenwood, B.M., Thomas, H.C., and Hill, A.V. (1995). Association between an MHC class II allele and clearance of hepatitis B virus in the Gambia. N. Engl. J. Med. *332*, 1065–1069.

54. Kummee, P., Tangkijvanich, P., Poovorawan, Y., and Hirankarn, N. (2007). Association of HLA-DRB1*13 and TNF-alpha gene polymorphisms with clearance of chronic hepatitis B infection and risk of hepatocellular carcinoma in Thai population. J. Viral Hepat. *14*, 841–848.

55. Singh, R., Kaul, R., Kaul, A., and Khan, K. (2007). A comparative review of HLA associations with hepatitis B and C viral infections across global populations. World J. Gastroenterol. *13*, 1770–1787.

56. Yan, Z.H., Fan, Y., Wang, X.H., Mao, Q., Deng, G.H., and Wang, Y.M. (2012). Relationship between HLA-DR gene polymorphisms and outcomes of hepatitis B viral infections: a meta-analysis. World J. Gastroenterol. *18*, 3119–3128.

57. Katrinli, S., Nilay Karatas Erkut, G., Ozdil, K., Yilmaz Enc, F., Ozturk, O., Kahraman, R., Tuncer, I., Dinler Doganay, G., and Doganay, L. (2017). HLA DPB1 15:01 allele predicts spontaneus hepatitis B surface antigen seroconversion. Acta Gastroenterol. Belg. *80*, 351–355.

58. Lv, N., Dang, A., Wang, Z., Zheng, D., and Liu, G. (2011). Association of susceptibility to Takayasu arteritis in Chinese Han patients with HLA-DPB1. Hum. Immunol. *72*, 893–896.

59. Doherty, D.G., Penzotti, J.E., Koelle, D.M., Kwok, W.W., Lybrand, T.P., Masewicz, S., and Nepom, G.T. (1998). Structural basis of specificity and degeneracy of T cell recognition: pluriallelic restriction of T cell responses to a peptide antigen involves both specific and promiscuous interactions between the T cell receptor, peptide, and HLA-DR. J. Immunol. *161*, 3527–3535.

60. Shi, Y., Kaliyaperumal, A., Lu, L., Southwood, S., Sette, A., Michaels, M.A., and Datta, S.K. (1998). Promiscuous presentation and recognition of nucleosomal autoepitopes in lupus: role of autoimmune T cell receptor alpha chain. J. Exp. Med. *187*, 367–378.

61. Cheng, X., Xia, Y., Serti, E., Block, P.D., Chung, M., Chayama, K., Rehermann, B., and Liang, T.J. (2017). Hepatitis B virus evades innate immunity of hepatocytes but activates cytokine production by macrophages. Hepatology *66*, 1779–1793.

62. Courtney, A.H., Lo, W.L., and Weiss, A. (2018). TCR signaling: mechanisms of initiation and propagation. Trends Biochem. Sci. *43*, 108–123.

63. Huppa, J.B., Axmann, M., Mörtelmaier, M.A., Lillemeier, B.F., Newell, E.W., Brameshuber, M., Klein, L.O., Schütz, G.J., and Davis, M.M. (2010). TCR-peptide-MHC interactions in situ show accelerated kinetics and increased affinity. Nature *463*, 963–967.

64. Jönsson, P., Southcombe, J.H., Santos, A.M., Huo, J., Fernandes, R.A., McColl, J., Lever, M., Evans, E.J., Hudson, A., Chang, V.T., et al. (2016). Remarkably low affinity of CD4/peptide-major histocompatibility complex class II protein interactions. Proc. Natl. Acad. Sci. U S A *113*, 5682–5687.

65. Ballek, O., Valečka, J., Manning, J., and Filipp, D. (2015). The pool of preactivated Lck in the initiation of T-cell signaling: a critical re-evaluation of the Lck standby model. Immunol. Cell Biol. *93*, 384–395.

66. Veillette, A., Bookman, M.A., Horak, E.M., and Bolen, J.B. (1988). The CD4 and CD8 T cell surface antigens are associated with the internal membrane tyrosine-protein kinase p56lck. Cell *55*, 301–308.

67. van der Veken, L.T., Hoogeboom, M., de Paus, R.A., Willemze, R., Falkenburg, J.H., and Heemskerk, M.H. (2005). HLA class II restricted T-cell receptor gene transfer generates CD4+ T cells with helper activity as well as cytotoxic capacity. Gene Ther. *12*, 1686–1695.

68. Raphael, I., Nalawade, S., Eagar, T.N., and Forsthuber, T.G. (2015). T cell subsets and their signature cytokines in autoimmune and inflammatory diseases. Cytokine *74*, 5–17.

69. Xia, Y., Stadler, D., Lucifora, J., Reisinger, F., Webb, D., Hösel, M., Michler, T., Wisskirchen, K., Cheng, X., Zhang, K., et al. (2016). Interferon-γ and tumor necrosis factor-α produced by T cells reduce the HBV persistence form, cccDNA, without cytolysis. Gastroenterology *150*, 194–205.

70. Lin, L., Couturier, J., Yu, X., Medina, M.A., Kozinetz, C.A., and Lewis, D.E. (2014). Granzyme B secretion by human memory CD4 T cells is less strictly regulated compared to memory CD8 T cells. BMC Immunol. *15*, 36.

71. Juno, J.A., van Bockel, D., Kent, S.J., Kelleher, A.D., Zaunders, J.J., and Munier, C.M. (2017). Cytotoxic CD4 T cells-friend or foe during viral infection? Front. Immunol. *8*, 19.

72. Takeuchi, A., and Saito, T. (2017). CD4 CTL, a cytotoxic subset of CD4(+) T cells, their differentiation and function. Front. Immunol. *8*, 194.

73. Appay, V., Zaunders, J.J., Papagno, L., Sutton, J., Jaramillo, A., Waters, A., Easterbrook, P., Grey, P., Smith, D., McMichael, A.J., et al. (2002). Characterization of CD4(+) CTLs ex vivo. J. Immunol. *168*, 5954–5958.

74. Norris, P.J., Moffett, H.F., Yang, O.O., Kaufmann, D.E., Clark, M.J., Addo, M.M., and Rosenberg, E.S. (2004). Beyond help: direct effector functions of human immunodeficiency virus type 1-specific CD4(+) T cells. J. Virol. *78*, 8844–8851.

75. Suni, M.A., Ghanekar, S.A., Houck, D.W., Maecker, H.T., Wormsley, S.B., Picker, L.J., Moss, R.B., and Maino, V.C. (2001). CD4(+)CD8(dim) T lymphocytes exhibit enhanced cytokine expression, proliferation and cytotoxic activity in response to HCMV and HIV-1 antigens. Eur. J. Immunol. *31*, 2512–2520.

76. van Leeuwen, E.M., Remmerswaal, E.B., Vossen, M.T., Rowshani, A.T., Wertheim-van Dillen, P.M., van Lier, R.A., and ten Berge, I.J. (2004). Emergence of a CD4+CD28- granzyme B+, cytomegalovirus-specific T cell subset after recovery of primary cytomegalovirus infection. J. Immunol. *173*, 1834–1841.

77. Brown, D.M., Lee, S., Garcia-Hernandez Mde, L., and Swain, S.L. (2012). Multifunctional CD4 cells expressing gamma interferon and perforin mediate protection against lethal influenza virus infection. J. Virol. *86*, 6792–6803.

78. Soghoian, D.Z., Jessen, H., Flanders, M., Sierra-Davidson, K., Cutler, S., Pertel, T., Ranasinghe, S., Lindqvist, M., Davis, I., Lane, K., et al. (2012). HIV-specific cytolytic CD4 T cell responses during acute HIV infection predict disease outcome. Sci. Transl Med. *4*, 123ra125.

79. Aslan, N., Yurdaydin, C., Wiegand, J., Greten, T., Ciner, A., Meyer, M.F., Heiken, H., Kuhlmann, B., Kaiser, T., Bozkaya, H., et al. (2006). Cytotoxic CD4 T cells in viral hepatitis. J. Viral Hepat. *13*, 505–514.

80. Knolle, P.A. (2016). Staying local-antigen presentation in the liver. Curr. Opin. Immunol. *40*, 36–42.

81. Herkel, J., Jagemann, B., Wiegard, C., Lazaro, J.F., Lueth, S., Kanzler, S., Blessing, M., Schmitt, E., and Lohse, A.W. (2003). MHC class II-expressing hepatocytes function as antigen-presenting cells and activate specific CD4 T lymphocytes. Hepatology *37*, 1079–1085.

82. Law, R.H., Zhang, Q., McGowan, S., Buckle, A.M., Silverman, G.A., Wong, W., Rosado, C.J., Langendorf, C.G., Pike, R.N., Bird, P.I., et al. (2006). An overview of the serpin superfamily. Genome Biol. *7*, 216.

83. Wei, S.C., Duffy, C.R., and Allison, J.P. (2018). Fundamental mechanisms of immune checkpoint blockade therapy. Cancer Discov. *8*, 1069–1086.

84. Lohse, A.W., Knolle, P.A., Bilo, K., Uhrig, A., Waldmann, C., Ibe, M., Schmitt, E., Gerken, G., and Meyer Zum Büschenfelde, K.H. (1996). Antigen-presenting function and B7 expression of murine sinusoidal endothelial cells and Kupffer cells. Gastroenterology *110*, 1175–1181.

85. Quezada, S.A., Simpson, T.R., Peggs, K.S., Merghoub, T., Vider, J., Fan, X., Blasberg, R., Yagita, H., Muranski, P., Antony, P.A., et al. (2010). Tumor-reactive CD4(+) T cells develop cytotoxic activity and eradicate large established melanoma after transfer into lymphopenic hosts. J. Exp. Med. *207*, 637–650.

86. Heinz, N., Schambach, A., Galla, M., Maetzig, T., Baum, C., Loew, R., and Schiedlmeier, B. (2011). Retroviral and transposon-based tet-regulated all-in-one vectors with reduced background expression and improved dynamic range. Hum. Gene Ther. *22*, 166–176.

OMTM, Volume *23*

Supplemental information

Characterization of a library of 20 HBV-specific MHC class II-restricted T cell receptors

Sophia Schreiber, Melanie Honz, Weeda Mamozai, Peter Kurktschiev, Matthias Schiemann, Klaus Witter, Eugene Moore, Christina Zielinski, Alessandro Sette, Ulrike Protzer, and Karin Wisskirchen

Supplemental figures

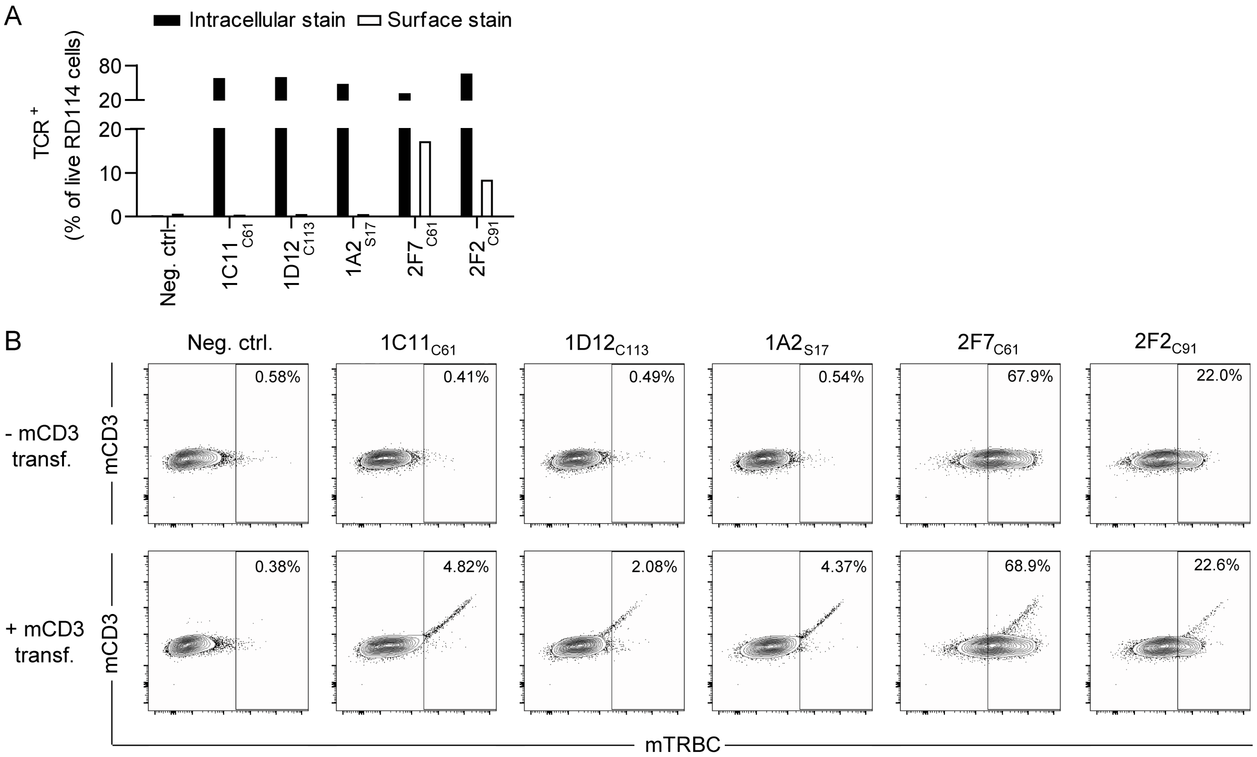

Figure S1: Generation of stable producer cell lines for the production of retroviral particles for transduction. Gibbon ape leukaemia virus (GALV) producer cells were transfected with the retroviral vector MP71 containing the respective transgenic TCR. The supernatant containing retroviral particles was used to transduce RD114 producer cells, resulting in a stable genomic integration of the TCR coding sequence. The resulting stably transduced RD114 cells were then enriched by flow cytometry cell sorting based on TCR surface expression. (A) Transduction rates of RD114 cells were determined by flow cytometry through intracellular (black bars) or surface staining (white bars) of the murine constant β-domain (mTRBC) of each TCR. The examples shown here include TCRs where surface staining was either possible or unsuccessful. (B) In producer cells showing little to no TCR surface expression, transient transfection (transf.) with the murine CD3 δγεζ-chains (mCD3) prior to enrichment increased and stabilized TCR surface expression sufficiently for cell sorting.

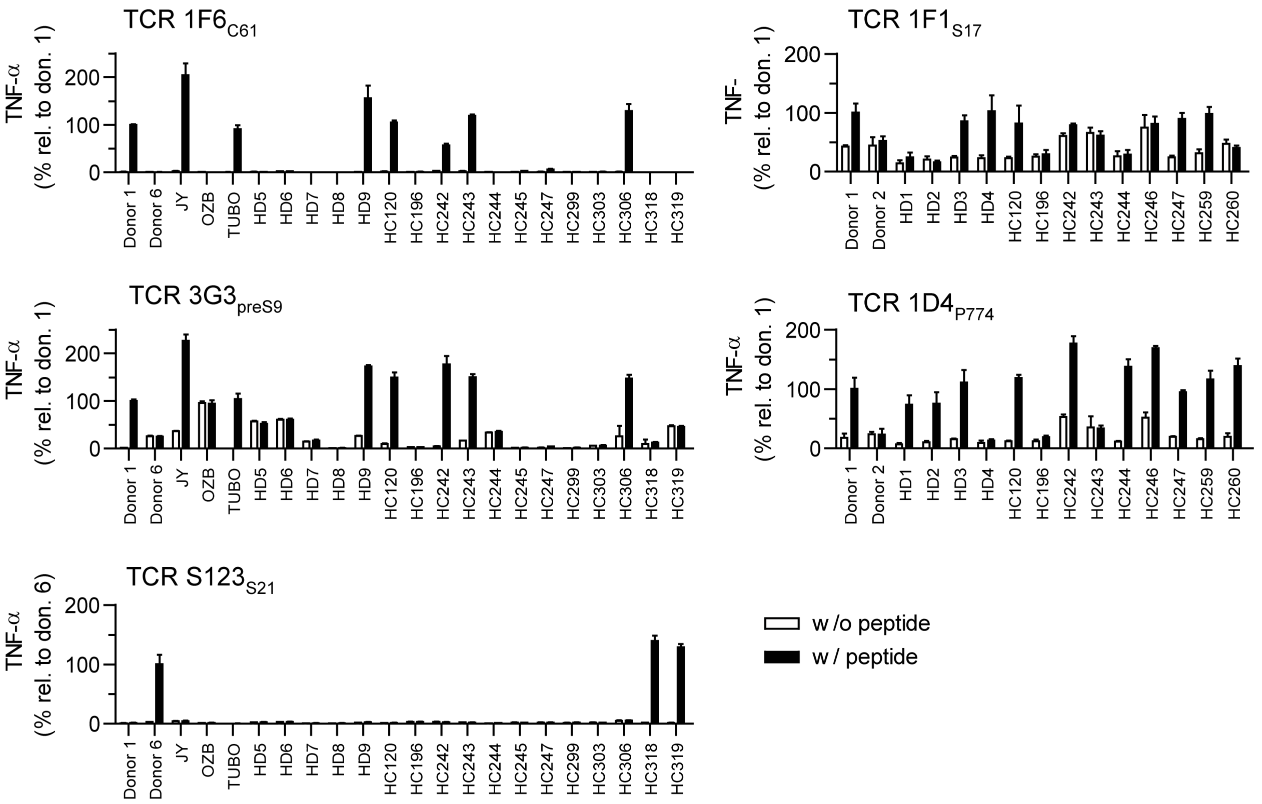

Figure S2: Co-culture of TCR-transduced T cells with partially HLA-matched B-LCLs to determine MHC class II restriction. T cells were transduced with TCRs 1F6$_{C61}$, 3G3$_{preS9}$, S123$_{S21}$, 1F1$_{S17}$ and 1D4$_{P774}$ and co-cultured at an E:T of 2:1 with partially HLA-matched B-LCLs, pulsed with 1 µM of the respective peptide (w/ peptide, black bars) or without peptide (w/o peptide, white bars). TNF-α secretion was determined via ELISA after 16 hours of co-culture and is shown relative to values from co-culture with donor B-LCLs. Data points represent mean values ± SD from triplicates. TCR clone names are indicated at the top left of each graph. The respective MHC II alleles of each B-LCL are summarized in Table S2.

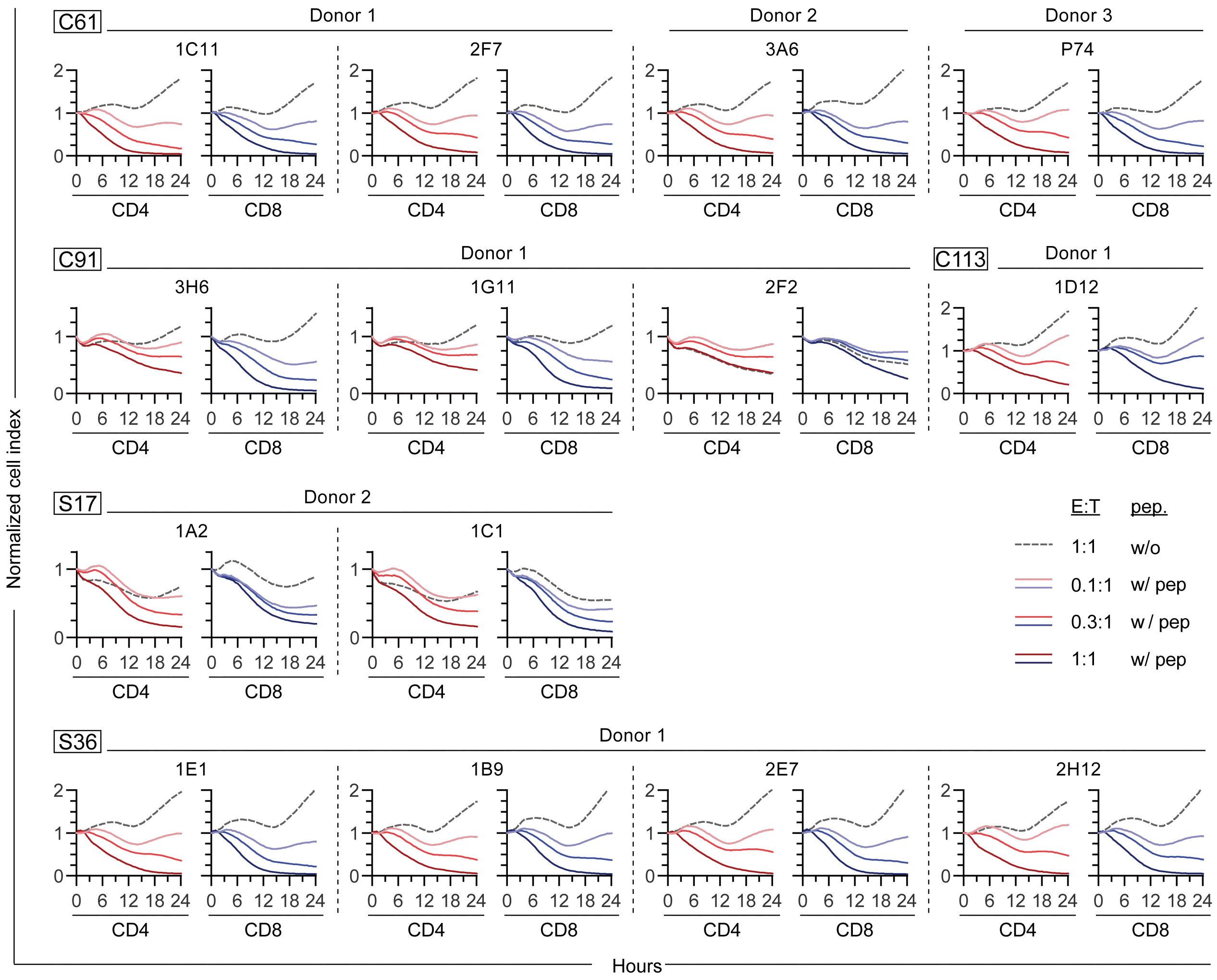

Figure S3: Kinetics of cytotoxicity of TCR-transduced CD4⁺ and CD8⁺ T cells. CD4⁺ (red) or CD8⁺ (blue) TCR-transduced T cells were co-cultured for 24 hours with single MHC II transfectant fibroblasts pulsed with 1 µM peptide (w/ pep) at different effector to target (E:T) cell ratios: 1:1 (dark color), 0.3:1 (medium color), or 0.1:1 (light color) or without peptide (w/o pep) at an E:T ratio of 1:1 (grey). Cytotoxicity was assessed via the adherence of target cells measured through electrical impedance and is given as a cell index normalized to the starting point of each co-culture. Considering the technical requirements of this assay, only TCRs were included, for which adherent single MHC II transfectant fibroblasts were available. Data points were acquired every 30 minutes for 24 hours and represent mean values from triplicates. Endpoint cytotoxicity data are shown in Figure 7. Square boxes at the top left of each graph indicate peptide specificities. Mock-transduced T cells served as an additional negative control and no killing was observed after 24 hours of co-culture (data not shown).

Supplemental tables

Table S1: Characteristics and sequences of peptides used for stimulation. Left side: Peptides from this study used for stimulation of PBMC of donors 1 and 2. Specificities for which TCRs were isolated are highlighted in bold. Binding affinities (IC_{50}) to HLA-DR1 and HLA-DR13 as predicted via the NetMHCIIpan 3.2 algorithm.[1] Predicted binding cores to DR1/DR13 are overlined/underlined in peptide sequence. IC_{50} values <50 nM or <500 nM are considered strong or weak binders, respectively. Right side: Literature review with therein published epitopes, corresponding HLA alleles and references.[2-11] Amino acid overlaps with peptides from this study are double underlined. n.d.= none determined.

Peptides from this study					Literature review		
HBV protein	Peptide name	Peptide sequence (predicted binding cores of DR1 and DR13)	Predicted affinity IC_{50} (nM) DR1	Predicted affinity IC_{50} (nM) DR13	Published peptide (overlap with peptides from this study)	HLA	Reference
Core	C7	KEFGATVELLSFLPSDFF	493	1855	MDIDPYKEFGATVELLSFLP	DR	(2)
Core	C28	RDLLDTASALYREALESP	530	964			
Core	**C61**	WGELMTLATWVGNNLEDP	696	2048	LCWGELMTLATWVGVN	DR1	(3)
Core	C84	LVVNYVNTNMGLKIRQLL	156	65			
Core	**C91**	TNMGLKIRQLLWFHISCL	387	193			
Core	**C113**	ETVLEYLVSFGVWIRTPP	205	884	GRETVIEYLVSFGVW<br>EYLVSFGVWIRTPPA	DR1<br>DRw52/DR6	(4)<br>(2)
Core	C119	LVSFGVWIRTPPAYRPPN	104	277	VSFGVWIRTPPAYRPPNAPI	DR1	(2)
Core	C133	RPPNAPILSTLPETTVVR	801	2446			
Envelope	**preS9**	RKGMGTNLSVPNPLGFFP	704	2025			
Envelope	preS83	GILTTVSTIPPPASTNRQ	786	2276			
Envelope	preS116	HPQAMQWNSTAFHQALQD	573	780	MQWNSTTFHQTLQDPRVRGLYFPAGG<br>MQWNSTALHQALQDP	DR1<br>DR2	(5)<br>(6)
Envelope	preS134	PRVRGLYFPAGGSSSGTV	149	4167			
Envelope	S8	FLGPLLVLQAGFFLLTRI	502	1574			
Envelope	**S17**	AGFFLLTRILTIPQSLDS	130	395	FFLLTRILTIPQSLD<br>FFLLTRILTIPQSLD<br>QAGFFLLTRILTIPQS	DR2<br>DR7<br>DR1	(7)<br>(8)<br>(5)
Envelope	**S36**	WTSLNFLGGSPVCLGQNS	77	2344	TSLNFLGGSPVCLGQ	DR1	(5)
Envelope	S69	CPGYRWMCLRRFIIFLFI	363	242	PICPGYRWMCLRRFIIFL	DR12	(8)
Envelope	S93	FLLVLLDYQGMLPVCPLI	144	1106	FLLVLLDYQGMLP	DP4	(9)
Envelope	S158	FAKYLWEWASVRFSWLSL	117	450	WEWASARFSWLSL<br>WASVRFSW	DP4<br>DR11/14	(9)<br>(10)
Envelope	S165	WASVRFSWLSLLVPFVQW	165	656	SVRFSWLSLLVPFVQWF	DP2	(8)
Envelope	S179	FVQWFVGLSPTVWLSAIW	68	813	SLLVPFVQWFVGLSPTVWLSV<br>VGLSPTVWLSVI	DR1<br>DP4	(5)<br>(9)
Envelope	S199	WYWGPSLYSIVSPFIPLL	161	1164	YWGPSLYSIVSPFIPL	DR3	(8)
Envelope	S209	VSPFIPLLPIFFCLWVYI	410	2029			
Polymerase	P104	NEKRRLKLIMPARFYPTH	40	33			
Polymerase	P412	PNLQSLTNLLSSNLSWLS	80	488	LQSLTNLLSSNLSWL	n.d.	(11)
Polymerase	P454	SGLSRYVARLSSNSRIFN	61	92			
Polymerase	P524	SPFLLAQFTSAICSVVRR	62	569			
Polymerase	P573	TNFLLSLGIHLNPNKTKR	63	157			
Polymerase	P636	QRIVGLLGFAAPFTQCGY	151	1112			
Polymerase	P650	QCGYPALMPLYACIQSKQ	250	1532			
Polymerase	**P774**	LRGTSFVYVPSALNPADD	22	908	AANWILRGTSFVYVP	n.d.	(11)
Polymerase	P827	HLPVRVHFASPLHVAWRP	47	109			

Table S2: MHC restriction analysis for TCRs 1F6$_{C61}$, 3G3$_{preS9}$, S123$_{S21}$, 1F1$_{S17}$ and 1D4$_{P774}$ using partially HLA-matched B-LCLs. B-LCLs which induced cytokine secretion in TCR-transduced T cells solely in the presence of the corresponding peptide are labeled as “specific”. B-LCLs which induced cytokine secretion both in the presence and absence of peptide and higher than the donor B-LCL unloaded control are labeled “unspecific”. No cytokine secretion in the presence or absence of peptide is labeled “negative”. MHC molecules shared exclusively among “specific” B-LCLs and the original donor’s B-LCLs (underlined) are marked in grey for each TCR.

TCR 1F1$_{S17}$															
	B-LCL	DRB1		DRB3		DRB4	DRB5	DQB1		DPB1		DQA1		DPA1	
Specific	Donor 1	01:01	13:01	02:02				05:01	06:03	02:01	04:01	01	01	01:03	
	HD3	07:01	15:01			01:03	01:01	02:02	05:01	02:01	04:01	01:02	02:01	01:03	
	HD4	07:01				01:03		03:03		02:01		02:01		01:03	
	HC120	13:01	13:02	01:01	03:01			06:03	06:04	03:01		01:02	01:03	01:03	
	HC247	15:01					01:01	06:02		02:01	04:02	01:02		01:03	
	HC259	04:01	08:02			01:03		03:02	04:02	02:01	04:01	03:01	04:01	01:03	
Negative	HD1	07:01				01:03		02:02		04:01	13:01	02:01		01:03	
	HD2	01:01	08:01					04:02	05:01	04:01	04:02	01:01	04:01	01:03	
	HC242	15:01					01:01	06:02	06:03	04:01		01:02		01:03	02:02
	HC244	03:01	07:01	01:01		01:03		02:01	02:02	04:01	10:01	02:01	01:03	01:03	02:01
	HC246	08:01						04:02		03:01	04:01	04:01	04:02	01:03	
	HC260	01:01	04:01			01:03		03:02	05:01	04:01	04:02	01:01	03:01	01:03	
	Donor 2	01:01	07:01			01:01		02:02	05:01	03:01	11:02	01	02:01	01:03	02:01
	HC243	13:01	13:02	01:01	03:01			06:03	06:04	03:01	11:01	01:02	01:03	01:03	02:01
	HC196	04:04				01:03		03:02	04:02	03:01	06:01	03:01	03:03	01:03	
TCR 1D4$_{P774}$															
	B-LCL	DRB1		DRB3		DRB4	DRB5	DQB1		DPB1		DQA1		DPA1	
Specific	Donor 1	01:01	13:01	02:02				05:01	06:03	02:01	04:01	01	01	01:03	
	HD1	07:01				01:03		02:02		04:01	13:01	02:01		01:03	
	HD2	01:01	08:01					04:02	05:01	04:01	04:02	01:01	04:01	01:03	
	HD3	07:01	15:01			01:03	01:01	02:02	05:01	02:01	04:01	01:02	02:01	01:03	
	HC120	13:01	13:02	01:01	03:01			06:03	06:04	03:01		01:02	01:03	01:03	
	HC242	15:01					01:01	06:02	06:03	04:01		01:02		01:03	02:02
	HC244	03:01	07:01	01:01		01:03		02:01	02:02	04:01	10:01	02:01	01:03	01:03	02:01
	HC246	08:01						04:02		03:01	04:01	04:01	04:02	01:03	
	HC247	15:01					01:01	06:02		02:01	04:02	01:02		01:03	
	HC259	04:01	08:02			01:03		03:02	04:02	02:01	04:01	03:01	04:01	01:03	
	HC260	01:01	04:01			01:03		03:02	05:01	04:01	04:02	01:01	03:01	01:03	
Unspecific	Donor 2	01:01	07:01			01:01		02:02	05:01	03:01	11:02	01	02:01	01:03	02:01
	HC243	13:01	13:02	01:01	03:01			06:03	06:04	03:01	11:01	01:02	01:03	01:03	02:01
Negative	HD4	07:01				01:03		03:03		02:01		02:01		01:03	
	HC196	04:04				01:03		03:02	04:02	03:01	06:01	03:01	03:03	01:03	

(Table S2 continued on next page)

TCR 1F6$_{C61}$

	B-LCL	DRB1		DRB3		DRB4	DRB5	DQB1		DPB1		DQA1		DPA1	
Specific	Donor 1	01:01	13:01	02:02				05:01	06:03	02:01	04:01	01	01	01:03	
	JY	04:04	13:01	01:01		01:03		03:02	06:03	02:01	04:01	01:03	03:01	01:03	
	TUBO	01:01	13:01	02:02				05:01	06:03	02:01	04:01	01:01	01:03	01:03	01:03
	HD9	01:01	11:04	02:02				03:01	05:01	04:01	04:02	01:01	05:05	01:03	
	HC120	13:01	13:02	01:01	03:01			06:03	06:04	03:01		01:02	01:03	01:03	
	HC242	15:01					01:01	06:02	06:03	04:01		01:02		01:03	02:02
	HC243	13:01	13:02	01:01	03:01			06:03	06:04	03:01	11:01	01:02	01:03	01:03	02:01
	HC306	11:01	13:01	02:02				03:01	06:03	04:01	13:01	01:03	05:05	01:03	02:01
Negative	Donor 6	04:03	11:04	02:02		01:03		03:01	03:02	04:01	15:01	03:01	05:05	01:03	
	OZB	11:04		02:02				03:01		04:02	10:01	05:05		01:03	02:01
	HD5	11:04	16:01	02:02			02:02	03:01	05:02	04:01	10:01	01:02	05:05	01:03	02:01
	HD6	04:02	11:01	02:02		01:03		03:01	03:02	02:01		03:01	05:05	01:03	
	HD7	03:01	04:01	01:01		01:03		02:01	03:01	01:01	16:01	03:03	05:01	01:03	02:01
	HD8	03:01	04:03	02:02		01:03		02:01	03:05	04:01	26:01	03:01	05:01	01:03	02:01
	HC196	04:04				01:03		03:02	04:02	03:01	06:01	03:01	03:03	01:03	
	HC244	03:01	07:01	01:01		01:03		02:01	02:02	04:01	10:01	02:01	05:01	01:03	02:01
	HC245	13:02	15:01	03:01			01:01	06:02	06:04	02:01	04:01	01:02		01:03	
	HC247	15:01					01:01	06:02		02:01	04:02	01:02		01:03	
	HC299	04:03	09:01			01:03		03:02	03:03	05:01	13:01	03:01	03:02	02:06	04:01
	HC303	04:04	13:03	01:01		01:03		03:01	03:02	04:01		03:01	05:05	01:03	01:03
	HC318	04:01	11:02	02:02		01:03		03:01		04:01	15:01	03:03	05:05	01:03	01:03
	HC319	03:01	11:04	02:02				02:01	03:01	04:01	15:01	05:01	05:05	01:03	01:04

TCR 3G3$_{preS9}$

	B-LCL	DRB1		DRB3		DRB4	DRB5	DQB1		DPB1		DQA1		DPA1	
Specific	Donor 1	01:01	13:01	02:02				05:01	06:03	02:01	04:01	01	01	01:03	
	JY	04:04	13:01	01:01		01:03		03:02	06:03	02:01	04:01	01:03	03:01	01:03	
	TUBO	01:01	13:01	02:02				05:01	06:03	02:01	04:01	01:01	01:03	01:03	01:03
	HD9	01:01	11:04	02:02				03:01	05:01	04:01	04:02	01:01	05:05	01:03	
	HC120	13:01	13:02	01:01	03:01			06:03	06:04	03:01		01:02	01:03	01:03	
	HC242	15:01					01:01	06:02	06:03	04:01		01:02		01:03	02:02
	HC243	13:01	13:02	01:01	03:01			06:03	06:04	03:01	11:01	01:02	01:03	01:03	02:01
	HC306	11:01	13:01	02:02				03:01	06:03	04:01	13:01	01:03	05:05	01:03	02:01
Unspecific	Donor 6	04:03	11:04	02:02		01:03		03:01	03:02	04:01	15:01	03:01	05:05	01:03	
	OZB	11:04		02:02				03:01		04:02	10:01	05:05		01:03	02:01
	HD5	11:04	16:01	02:02			02:02	03:01	05:02	04:01	10:01	01:02	05:05	01:03	02:01
	HD6	04:02	11:01	02:02		01:03		03:01	03:02	02:01		03:01	05:05	01:03	
	HD7	03:01	04:01	01:01		01:03		02:01	03:01	01:01	16:01	03:03	05:01	01:03	02:01
	HC244	03:01	07:01	01:01		01:03		02:01	02:02	04:01	10:01	02:01	05:01	01:03	02:01
	HC318	04:01	11:02	02:02		01:03		03:01		04:01	15:01	03:03	05:05	01:03	01:03
	HC319	03:01	11:04	02:02				02:01	03:01	04:01	15:01	05:01	05:05	01:03	01:04
Negative	HD8	03:01	04:03	02:02		01:03		02:01	03:05	04:01	26:01	03:01	05:01	01:03	02:01
	HC196	04:04				01:03		03:02	04:02	03:01	06:01	03:01	03:03	01:03	
	HC245	13:02	15:01	03:01			01:01	06:02	06:04	02:01	04:01	01:02		01:03	
	HC247	15:01					01:01	06:02		02:01	04:02	01:02		01:03	
	HC299	04:03	09:01			01:03		03:02	03:03	05:01	13:01	03:01	03:02	02:06	04:01
	HC303	04:04	13:03	01:01		01:03		03:01	03:02	04:01		03:01	05:05	01:03	01:03

TCR S123$_{S21}$

	B-LCL	DRB1		DRB3		DRB4	DRB5	DQB1		DPB1		DQA1		DPA1	
Specific	Donor 6	04:03	11:04	02:02		01:03		03:01	03:02	04:01	15:01	03:01	05:05	01:03	01:04
	HC318	04:01	11:02	02:02		01:03		03:01		04:01	15:01	03:03	05:05	01:03	01:03
	HC319	03:01	11:04	02:02				02:01	03:01	04:01	15:01	05:01	05:05	01:03	01:04
Negative	Donor 1	01:01	13:01	02:02				05:01	06:03	02:01	04:01	01	01	01:03	
	JY	04:04	13:01	01:01		01:03		03:02	06:03	02:01	04:01	01:03	03:01	01:03	
	OZB	11:04		02:02				03:01		04:02	10:01	05:05		01:03	02:01
	TUBO	01:01	13:01	02:02				05:01	06:03	02:01	04:01	01:01	01:03	01:03	01:03
	HD5	11:04	16:01	02:02			02:02	03:01	05:02	04:01	10:01	01:02	05:05	01:03	02:01
	HD6	04:02	11:01	02:02		01:03		03:01	03:02	02:01		03:01	05:05	01:03	
	HD7	03:01	04:01	01:01		01:03		02:01	03:01	01:01	16:01	03:03	05:01	01:03	02:01
	HD8	03:01	04:03	02:02		01:03		02:01	03:05	04:01	26:01	03:01	05:01	01:03	02:01
	HD9	01:01	11:04	02:02				03:01	05:01	04:01	04:02	01:01	05:05	01:03	
	HC120	13:01	13:02	01:01	03:01			06:03	06:04	03:01		01:02	01:03	01:03	
	HC196	04:04				01:03		03:02	04:02	03:01	06:01	03:01	03:03	01:03	
	HC242	15:01					01:01	06:02	06:03	04:01		01:02		01:03	02:02
	HC243	13:01	13:02	01:01	03:01			06:03	06:04	03:01	11:01	01:02	01:03	01:03	02:01
	HC244	03:01	07:01	01:01		01:03		02:01	02:02	04:01	10:01	02:01	05:01	01:03	02:01
	HC245	13:02	15:01	03:01			01:01	06:02	06:04	02:01	04:01	01:02		01:03	
	HC247	15:01					01:01	06:02		02:01	04:02	01:02		01:03	
	HC299	04:03	09:01			01:03		03:02	03:03	05:01	13:01	03:01	03:02	02:06	04:01
	HC303	04:04	13:03	01:01		01:03		03:01	03:02	04:01		03:01	05:05	01:03	01:03
	HC306	11:01	13:01	02:02				03:01	06:03	04:01	13:01	01:03	05:05	01:03	02:01

(Table S2 continued)

Table S3: Characteristics of the specific peptide:MHC complex identified for each TCR. Left side: Overview of MHC restrictions for each TCR as identified in Figure 3, Figure S2 and Table S2. Right side: Binding affinities of HBV peptides to their restricting MHC allele predicted via the IEDB MHC-II binding prediction tool (http://tools.iedb.org/mhcii/), with IC_{50} values from recommended algorithms NetMHCIIpan or NN-align. In addition, binding affinities were measured by an MHC-ligand binding assay.[12]

<table>
<tr><th rowspan="2">TCR</th><th rowspan="2">Peptide</th><th colspan="2">Restricting MHC molecule</th><th colspan="2">Binding affinity pMHC IC_{50} (nM)</th></tr>
<tr><th>α-chain</th><th>β-chain</th><th>Predicted</th><th>Measured</th></tr>
<tr><td>1C11</td><td rowspan="5">C61</td><td>DRA</td><td>DRB3*02:02</td><td>3700.0</td><td>4880.0</td></tr>
<tr><td>2F7</td><td rowspan="3">DRA</td><td rowspan="3">DRB1*01:01</td><td rowspan="3">32.4</td><td rowspan="3">7.8</td></tr>
<tr><td>3A6</td></tr>
<tr><td>P74</td></tr>
<tr><td>1F6</td><td>DQA1*01:01</td><td>DQB1*06:03</td><td>232.1</td><td>382.0</td></tr>
<tr><td>3H6</td><td rowspan="3">C91</td><td rowspan="3">DRA</td><td rowspan="3">DRB1*13:01</td><td rowspan="3">193.4</td><td rowspan="3">3222.0</td></tr>
<tr><td>1G11</td></tr>
<tr><td>2F2</td></tr>
<tr><td rowspan="2">1D12</td><td rowspan="3">C113</td><td>DRA</td><td>DRB1*01:01</td><td>12.1</td><td>27.8</td></tr>
<tr><td>DQA1*01:01</td><td>DQB1*05:01</td><td>274.2</td><td>17.9</td></tr>
<tr><td>CP11</td><td>DRA</td><td>DRB3*02:02</td><td>1566.0</td><td>3030.0</td></tr>
<tr><td>3G3</td><td>preS9</td><td>DQA1*01:01</td><td>DQB1*06:03</td><td>391.0</td><td>3240.0</td></tr>
<tr><td>1F1</td><td rowspan="3">S17</td><td>DPA1*01:03</td><td>DPB1*02:01</td><td>2.2</td><td>1.0</td></tr>
<tr><td>1A2</td><td rowspan="2">DRA</td><td rowspan="2">DRB1*07:01</td><td rowspan="2">44.3</td><td rowspan="2">1.3</td></tr>
<tr><td>1C1</td></tr>
<tr><td>S123</td><td>S21</td><td>DPA1*01:03</td><td>DPB1*15:01</td><td>71.2</td><td>n.a.</td></tr>
<tr><td>1E1</td><td rowspan="4">S36</td><td rowspan="4"></td><td rowspan="4">DRB1*01:01</td><td rowspan="4">5.7</td><td rowspan="4">3.1</td></tr>
<tr><td>1B9</td></tr>
<tr><td>2E7</td></tr>
<tr><td>2H12</td></tr>
<tr><td>1D4</td><td>P774</td><td>DPA1*01:03</td><td>DPB1*04:01</td><td>382.2</td><td>0.9</td></tr>
</table>

Table S4: Summary table of TCR characterization. Transduction (transd.) rates in % of $CD4^{+}$ T cells and vector copy number (VCN) as an average number of integrates per cell (avg./cell) are indicated for a representative cell batch. MFI of TCR^{+} populations in flow cytometry from four independent transductions is normalized (norm.) to mean of each experiment. The recognition of processed (proc.) antigen is scored according to TNF-α secretion in co-culture with B-LCLs: low (<100 pg/ml), medium (100-200 pg/ml) and high (>200 pg/ml). The number of recognized HBV genotypes (Gt) by each TCR is given as a number of four (x/4) genotypes tested: A/B/C/D. Functional avidity is specified as EC_{50} in nM calculated from proliferation assays with peptide titration. $CD4^{+}IL\text{-}2^{+}$ cells and $CD8^{+}GrzB^{+}$ in % of TCR^{+} T cells are listed representative for cytokine secretion. Cytotoxicity endpoint values for $CD4^{+}$ and $CD8^{+}$ T cells after 24 hours of co-culture are indicated in % relative (rel.) to the unloaded control. n.d.= none determined; EC_{50} could not be calculated when a plateau of maximum response was not reached and cytotoxicity could not be determined when matching single MHCII-transfectant adherent target cell lines were not available.

TCR	Peptide	MHC restriction β-chain	Transd. rate (%)	VCN (avg./cell)	MFI (norm.)	Proc. antigen (score)	HBV Gt (x/4)	EC_{50} (nM)	Cytokine secretion (% of TCR^{+}T cells)		Cytotoxicity 24 h (% rel. to control)	
									CD4 (IL-2)	CD8 (GrzB)	CD4	CD8
1C11	C61	DRB1*01:01	87.6	4.7	0.70	low	4	82	89.3	92.6	2.4	2.8
2F7			67.3	2.0	1.24	low	4	83	87.1	82.7	4.6	2.5
3A6			70.6	1.9	0.89	low	4	94	87.0	83.5	4.0	2.7
P74			84.5	3.1	0.65	low	4	47	81.0	69.9	4.7	3.2
1F6		DQB1*06:03	76.7	1.8	1.57	low	1	92	78.5	73.2	n.d.	
3H6	C91	DRB1*13:01	73.2	2.1	1.09	high	3	n.d.	61.9	46.5	30.5	3.7
1G11			59.3	1.2	1.28	high	3	n.d.	69.2	47.7	34.3	8.0
2F2			84.6	2.8	0.89	low	4	n.d.	25.8	11.3	n.d.	
1D12	C113	DRB1*01:01	59.2	3.9	0.26	high	4	n.d.	70.6	70.0	10.9	5.3
CP11		DRB3*02:02	75.1	2.6	0.78	high	4	n.d.	76.4	72.3	n.d.	
3G3	preS9	DQB1*06:03	86.0	2.6	1.61	n.d.	3	42	76.4	82.3	n.d.	
1F1	S17	DPB1*02:01	78.0	2.6	1.06	low	3	3.3	88.8	86.1	39.3	4.9
1A2		DRB1*07:01	75.9	4.4	0.48	low	3	8	89.8	86.0	21.1	22.5
1C1			71.5	2.7	0.62	high	3	4.7	88.3	75.2	24.1	16.2
S123	S21	DPB1*15:01	75.7	2.6	0.71	low	4	9.9	66.8	65.7	n.d.	
1E1	S36	DRB1*01:01	65.1	2.5	0.32	high	1	3.4	92.3	90.3	2.7	2.1
1B9			75.6	2.5	0.59	high	1	8.5	89.3	84.9	3.3	2.0
2E7			72.4	1.7	1.30	med	1	3.2	86.8	75.7	2.9	1.8
2H12			85.5	2.4	2.56	med	1	8.8	81.2	73.1	3.1	2.5
1D4	P774	DPB1*04:01	82.7	2.8	1.20	n.d.	4	1.6	85.2	78.5	n.d.	

Supplemental methods

Stimulation of PBMC

PBMC were stimulated as described in the main manuscript. For stimulation of PBMC of donors 1 and 2, which resulted in identification of 17 out of the 20 TCRs, single peptides from Table S1 were used. The C61-specific T cell clone from donor 3 was obtained from the group of N. Gruener[10] and restimulated once with 1 µM of peptide C61, which resulted in identification of TCR $P74_{C61}$. For stimulation of donor 4, a pool of HBV core peptides was used at a final concentration of 2.7 µg/ml of each peptide (MDIDPYKEFGATVEL, LSFLPSDFFPSVRDL, FLPSDFFPSVRDLLD, RDLLDTASALYREAL, PHHTALRQAILCWGE, GRETVLEYLVSFGVW, EYLVSFGVWIR-TPPA, VSFGVWIRTPPAYRP, TVVRRRDRGRSPRRR), which resulted in identification of TCR $CP11_{C113}$. For stimulation of donor 5, a pool of HBV S peptides was used at a final concentration of 11 µg/ml of each peptide (LVLQAGFFLLTRILT, AGFFLLTRILTIPKS, LLTRILTIPKSLDSW, FFLLTRILTIPQSLD), which resulted in identification of TCR $S123_{S17}$.

Identification of HBV-specific T cells clones

96-well plates were microscopically screened for growing T cell clones. Donor-derived B-LCLs were irradiated (50 Gy), loaded with 1 µM of the respective peptide for 2 hours at 37 C°C and then washed twice with PBS. 20 µl/well of each visually outgrown T cell clone was co-cultured with $5x10^4$ peptide-pulsed or unloaded B-LCLs at 37 °C. TNF-α secretion via ELISA (BD) was measured after 16 hours from supernatants to determine HBV specificity.

Analysis of TCR repertoire

For RNA extraction from T cell clones, Trizol (Thermo Fisher Scientific) was used according to the manufacturer's instructions including 1-bromo-3-chloropropane (Sigma-Aldrich) and 20 µl Linear Acrylamide (Thermo Fisher Scientific). RNA was reverse transcribed to cccDNA using Superscript II (Thermo Fisher Scientific). TCR chains were amplified from cDNA with Illustra PureTaq PCR Beads (GE) using degenerated primers, VPANHUM (5'-TGAGTGTCCCPGAPGG2P-3') and CA2 (5'-GTGACACATTTGTTTGAGAATC-3') for α-chains, VP1 (5'-GCIITKTIYTGGTAYMGACA-3') or VP2 (5'-CTITKTWTTGGTAYCIKCAG-3') and CP1 (5'-GCACCTCCTTCCCATTCAC-3') for β-chains. The sequencing results were blasted with IMGT/V-QUEST to identify TCR chains. When degenerated primer PCR did not give a conclusive result, PCRs were repeated with primers specific for the individual α- or β-variable chain as described elsewhere.[13]

Cloning of TCR chains

5' Primers including a Kozak sequence and a NotI restriction site were designed according to the variable region identified for each TCR. An EcoRI or BsrGI restriction site was added to the 3' primers for the constant regions: TRAC (TRAC-EcoRI 5'-GGAATTCTCAGCTGGACCACAGCCGCAGC-3'and TRAC1-BsrGI 5'-CTTGTACATCAGCTGGACCACAGCCGCAGC-3') or TRBC (TRBC1-EcoRI 5'-TGGAATTCTCAGAAATCCTTTCTCTTGACC-3' and TRBC2-EcoRI 5'-TGGAATTCCTAGCCTCTGGAATCCTTTCTC-3'). TCR chains were amplified from cDNA with Phusion Hot Start II (New England Biolabs) and cloned separately into the retroviral vector MP71.[14] Variable domains of TCRs with confirmed HBV-specificity were codon-optimized and synthesized at GeneArt (Regensburg), fused by a P2A element and substituted with murine constant domains as described previously.[15]

Generation of stable producer cells

Stable 293GP-R30 (RD114-pseudotype) producer cells were generated by transduction with cell culture supernatant from 293GP-GLV9 cells[16] that had been transfected with TCR plasmids as described earlier.[17] Producer cell lines were transiently transfected with the murine CD3 δγεζ chains cloned into the vector pcDNA3.1 from the vector pMIG II Murine CD3 WTdelta-F2A-gamma-T2A-epsilon-P2A-zeta (Addgene) using the Lipofectamine 2000 transfection reagent (Thermo Fisher Scientific) prior to enrichment of TCR^+ cells with a FACS Aria II (BD) or a MoFlo II cell sorter (Beckmann Coulter).

Prediction of peptide to MHC binding affinity

Binding affinities of HBV peptides to the respective MHC allele were predicted using the NetMHCIIpan 3.2 algorithm based on 18-mers[1] or the IEDB MHC-II binding prediction tool (http://tools.iedb.org/mhcii/) using the recommended algorithms based on 15-mers.

MHC-ligand binding assay

Binding affinities of HBV peptides to their restricting MHC allele were measured based on their ability to inhibit the binding of a radiolabeled probe peptide to the purified MHC molecule as described previously.[12]

Supplemental references

1. Jensen, K.K., Andreatta, M., Marcatili, P., Buus, S., Greenbaum, J.A., Yan, Z., Sette, A., Peters, B., Nielsen, M. (2018). Improved methods for predicting peptide binding affinity to MHC class II molecules. Immunology 154, 394-406.

2. Barnaba, V., Franco, A., Alberti, A., Balsano, C., Benvenuto, R., Balsano, F. (1989). Recognition of hepatitis B virus envelope proteins by liver-infiltrating T lymphocytes in chronic HBV infection. J Immunol 143, 2650-2655.

3. Barnaba, V., Franco, A., Paroli, M., Benvenuto, R., De Petrillo, G., Burgio, V.L., Santilio, I., Balsano, C., Bonavita, M.S., Cappelli, G., et al. (1994). Selective expansion of cytotoxic T lymphocytes with a CD4+CD56+ surface phenotype and a T helper type 1 profile of cytokine secretion in the liver of patients chronically infected with Hepatitis B virus. J Immunol 152, 3074-3087.

4. Chisari, F.V., Ferrari, C. (1995). Hepatitis B virus immunopathogenesis. Annu Rev Immunol 13, 29-60.

5. Desombere, I., Gijbels, Y., Verwulgen, A., Leroux-Roels, G. (2000). Characterization of the T cell recognition of hepatitis B surface antigen (HBsAg) by good and poor responders to hepatitis B vaccines. Clin Exp Immunol 122, 390-399.

6. Ferrari, C., Bertoletti, A., Penna, A., Cavalli, A., Valli, A., Missale, G., Pilli, M., Fowler, P., Giuberti, T., Chisari, F.V., et al. (1991). Identification of immunodominant T cell epitopes of the hepatitis B virus nucleocapsid antigen. J Clin Invest 88, 214-222.

7. Honorati, M.C., Dolzani, P., Mariani, E., Piacentini, A., Lisignoli, G., Ferrari, C., Facchini, A. (1997). Epitope specificity of Th0/Th2 CD4+ T-lymphocyte clones induced by vaccination with rHBsAg vaccine. Gastroenterology 112, 2017-2027.

8. Mizukoshi, E., Sidney, J., Livingston, B., Ghany, M., Hoofnagle, J.H., Sette, A., Rehermann, B. (2004). Cellular immune responses to the hepatitis B virus polymerase. J Immunol 173, 5863-5871.

9. Pajot, A., Michel, M.L., Mancini-Bourgine, M., Ungeheuer, M.N., Ojcius, D.M., Deng, Q., Lemonnier, F.A., Lone, Y.C. (2006). Identification of novel HLA-DR1-restricted epitopes from the hepatitis B virus envelope protein in mice expressing HLA-DR1 and vaccinated human subjects. Microbes Infect 8, 2783-2790.

10. Raziorrouh, B., Heeg, M., Kurktschiev, P., Schraut, W., Zachoval, R., Wendtner, C., Wächtler, M., Spannagl, M., Denk, G., Ulsenheimer, A., et al. (2014). Inhibitory phenotype of HBV-specific CD4+ T-cells is characterized by high PD-1 expression but absent coregulation of multiple inhibitory molecules. PLoS One 9, e105703.

11. Ru, Z., Xiao, W., Pajot, A., Kou, Z., Sun, S., Maillere, B., Zhao, G., Ojcius, D.M., Lone, Y.C., Zhou, Y. (2012). Development of a humanized HLA-A2.1/DP4 transgenic mouse model and the use of this model to map HLA-DP4-restricted epitopes of HBV envelope protein. PLoS One 7, e32247.

12. Sidney, J., Southwood, S., Moore, C., Oseroff, C., Pinilla, C., Grey, H.M., Sette, A. (2013). Measurement of MHC/peptide interactions by gel filtration or monoclonal antibody capture. Curr Protoc Immunol Chapter 18, Unit 18.13.

13. Steinle, A., Reinhardt, C., Jantzer, P., Schendel, D.J. (1995). In vivo expansion of HLA-B35 alloreactive T cells sharing homologous T cell receptors: evidence for maintenance of an oligoclonally dominated allospecificity by persistent stimulation with an autologous MHC/peptide complex. J Exp Med 181, 503-513.

14. Engels, B., Cam, H., Schüler, T., Indraccolo, S., Gladow, M., Baum, C., Blankenstein, T., Uckert, W. (2003). Retroviral vectors for high-level transgene expression in T lymphocytes. Hum Gene Ther 14, 1155-1168.

15. Sommermeyer, D., Uckert, W. (2010). Minimal amino acid exchange in human TCR constant regions fosters improved function of TCR gene-modified T cells. J Immunol 184, 6223-6231.

16. Ghani, K., Wang, X., de Campos-Lima, P.O., Olszewska, M., Kamen, A., Riviere, I., Caruso, M. (2009). Efficient human hematopoietic cell transduction using RD114- and GALV-pseudotyped retroviral vectors produced in suspension and serum-free media. Hum Gene Ther 20, 966-974.

17. Wisskirchen, K., Metzger, K., Schreiber, S., Asen, T., Weigand, L., Dargel, C., Witter, K., Kieback, E., Sprinzl, M.F., Uckert, W., et al. (2017). Isolation and functional characterization of hepatitis B virus-specific T-cell receptors as new tools for experimental and clinical use. PLoS One 12, e0182936.
